# Supplementary figures and images for: Suggestion of creatine as a new neurotransmitter by approaches ranging from chemical analysis and biochemistry to electrophysiology (part 1 of 2)
Source: eLife. 2023 Dec 21;12:RP89317. doi: 10.7554/eLife.89317 (PMC10735228; doi:10.7554/eLife.89317)

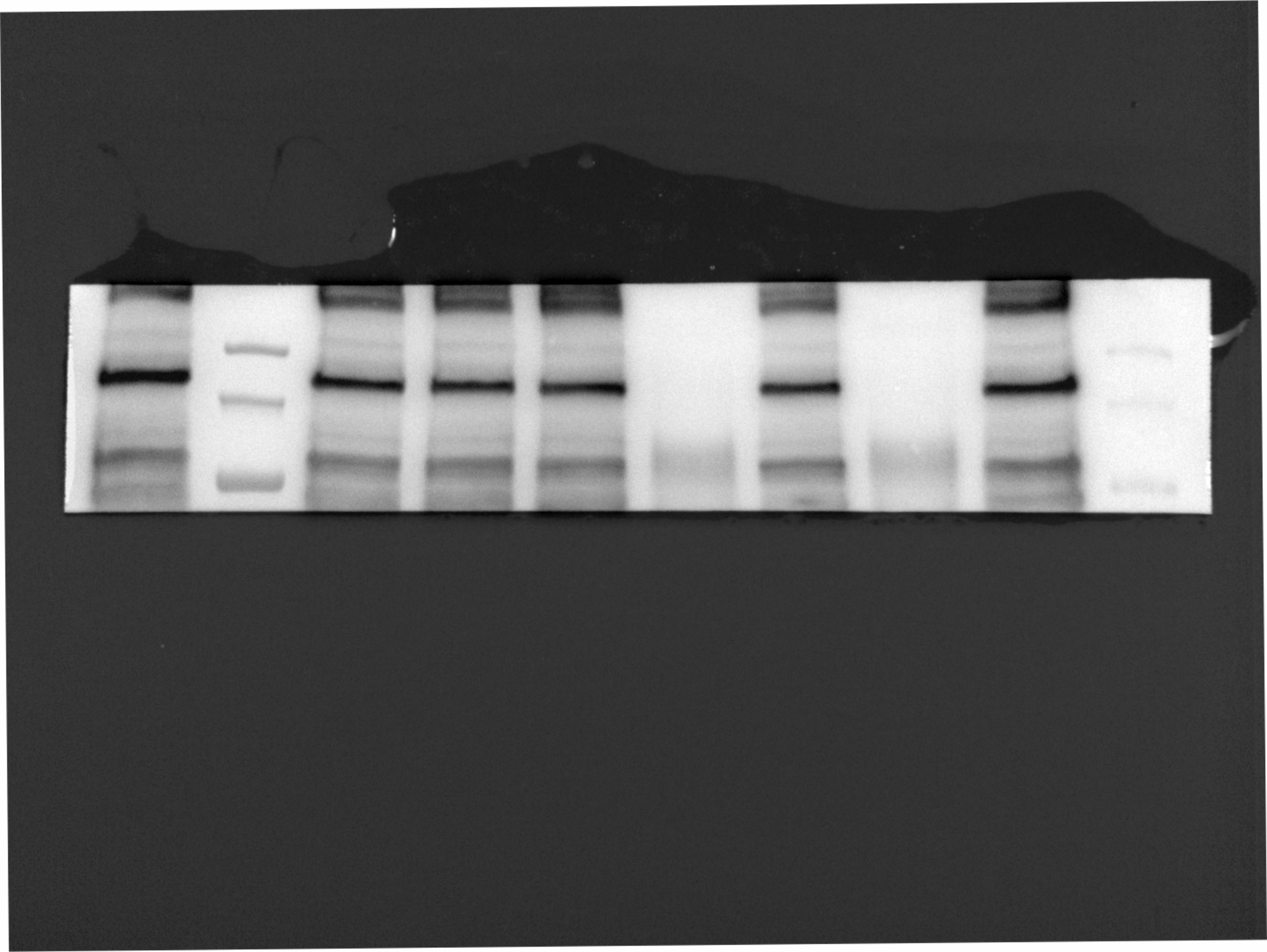

Supplement: Figure 1—figure supplement 1—source data 2. [file elife-89317-fig1-figsupp1-data2.zip › Original figures/CACNA1A Original.jpg]

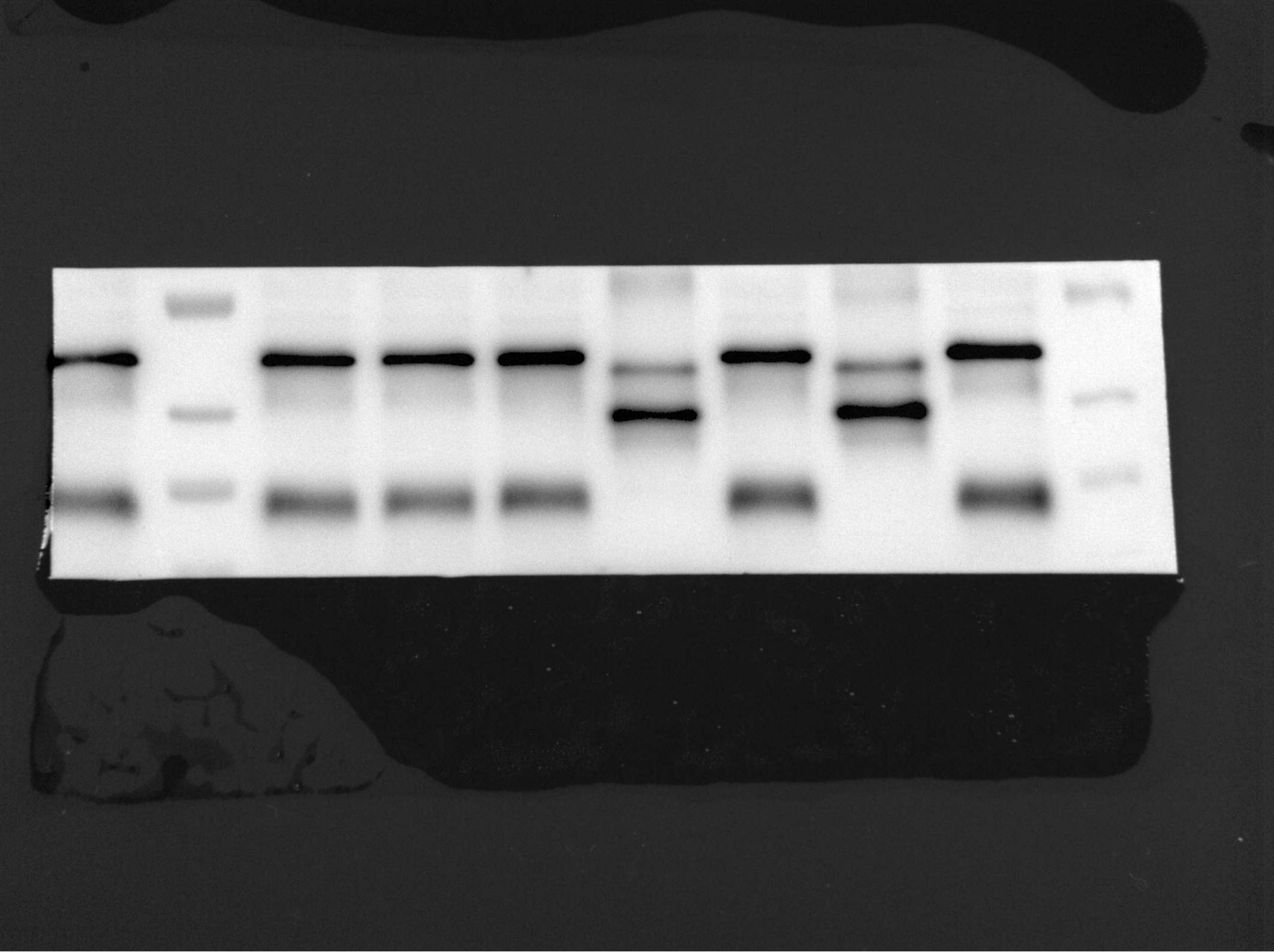

Supplement: Figure 1—figure supplement 1—source data 2. [file elife-89317-fig1-figsupp1-data2.zip › Original figures/cathepsinB Original.jpg]

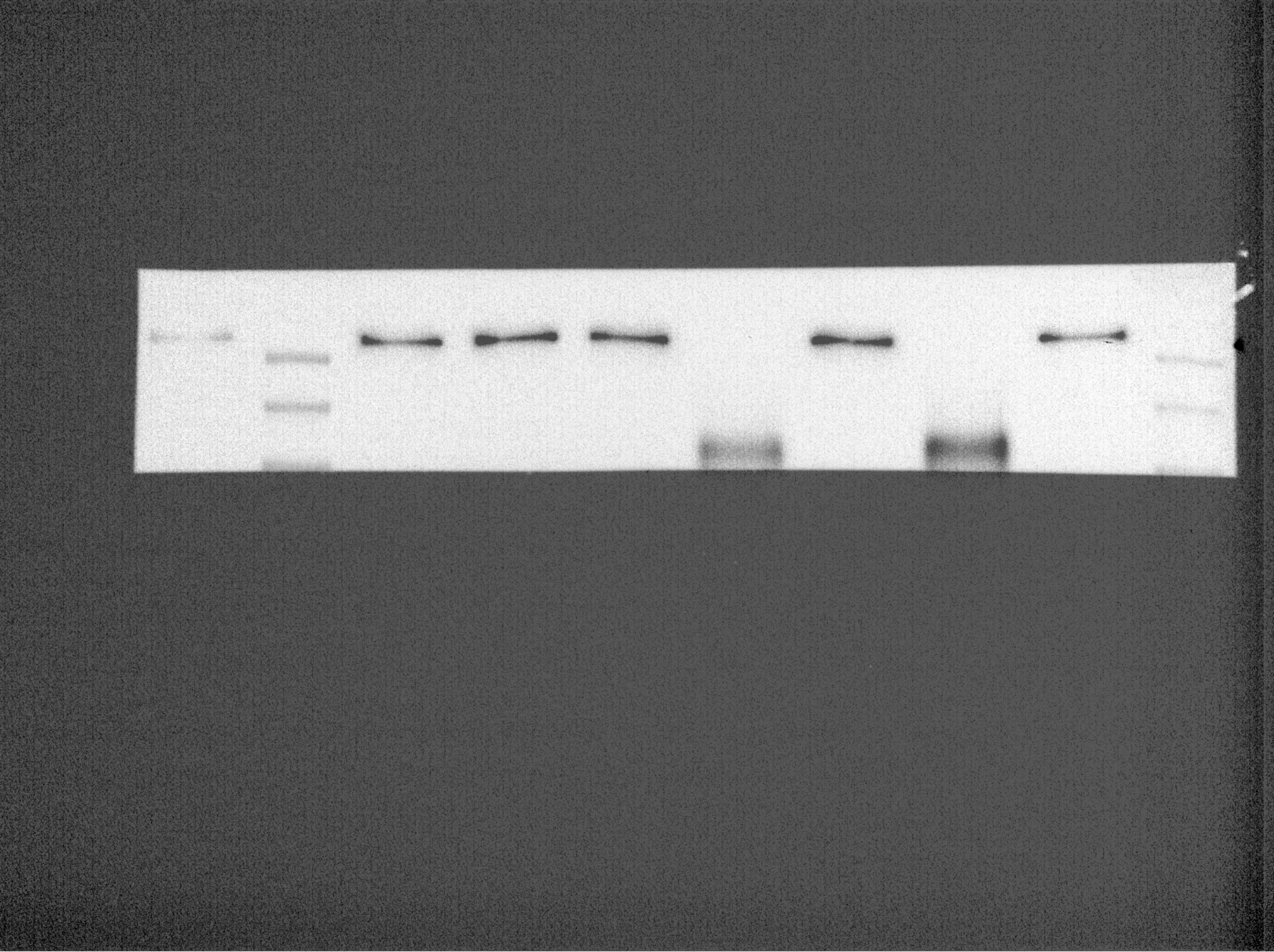

Supplement: Figure 1—figure supplement 1—source data 2. [file elife-89317-fig1-figsupp1-data2.zip › Original figures/EEA1 Original.jpg]

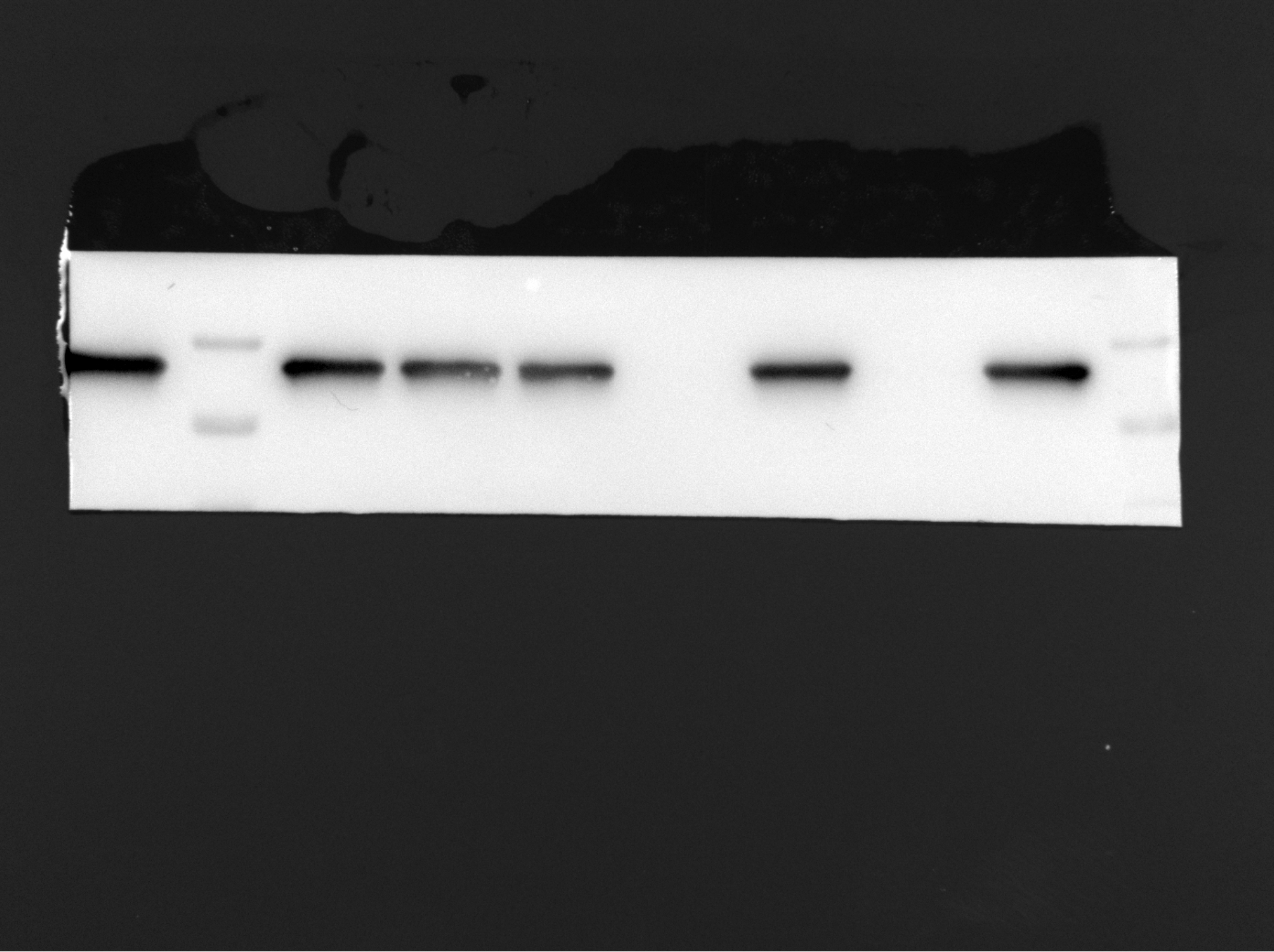

Supplement: Figure 1—figure supplement 1—source data 2. [file elife-89317-fig1-figsupp1-data2.zip › Original figures/GAPDH Original.jpg]

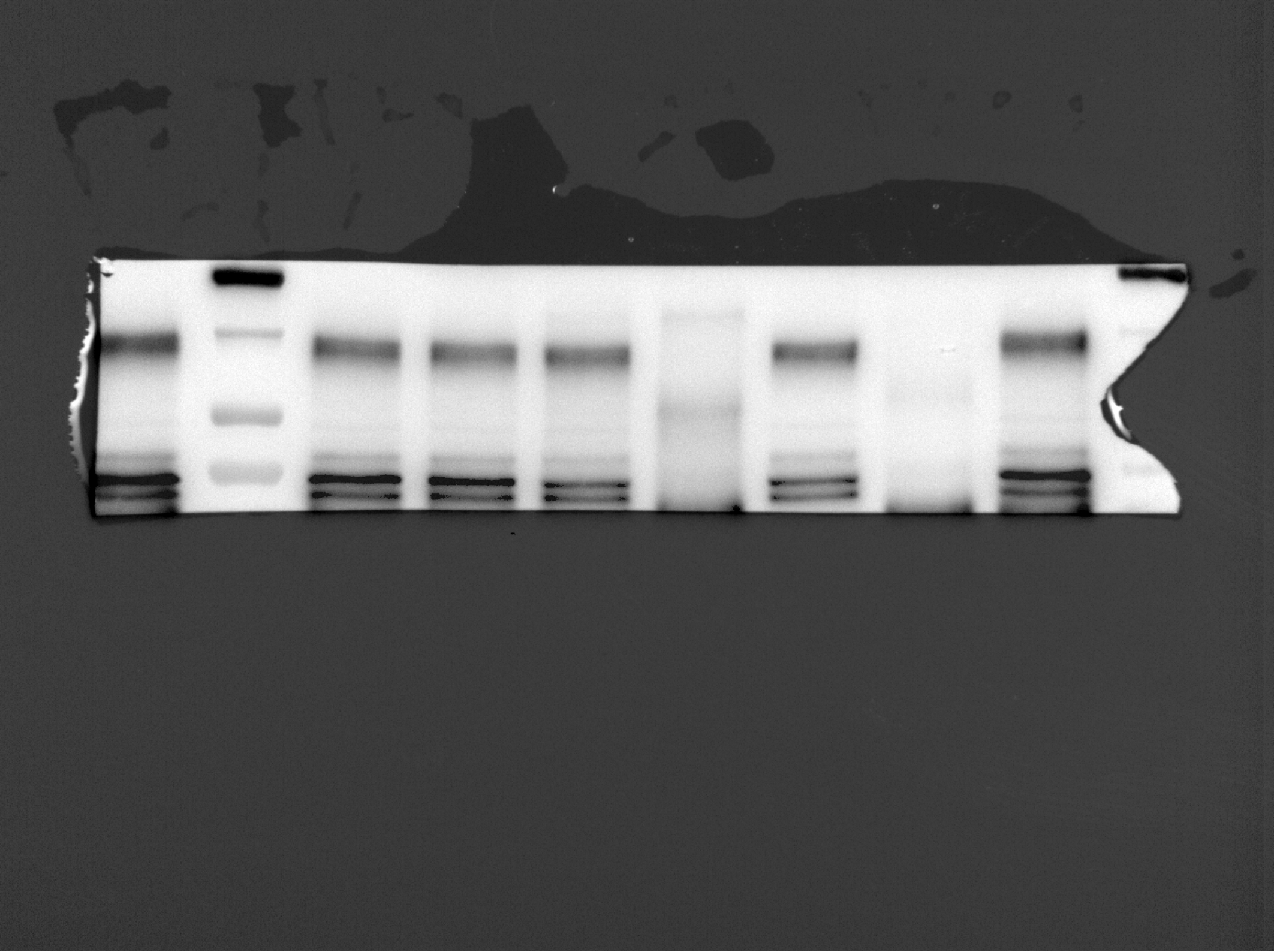

Supplement: Figure 1—figure supplement 1—source data 2. [file elife-89317-fig1-figsupp1-data2.zip › Original figures/Glu N1 Original.jpg]

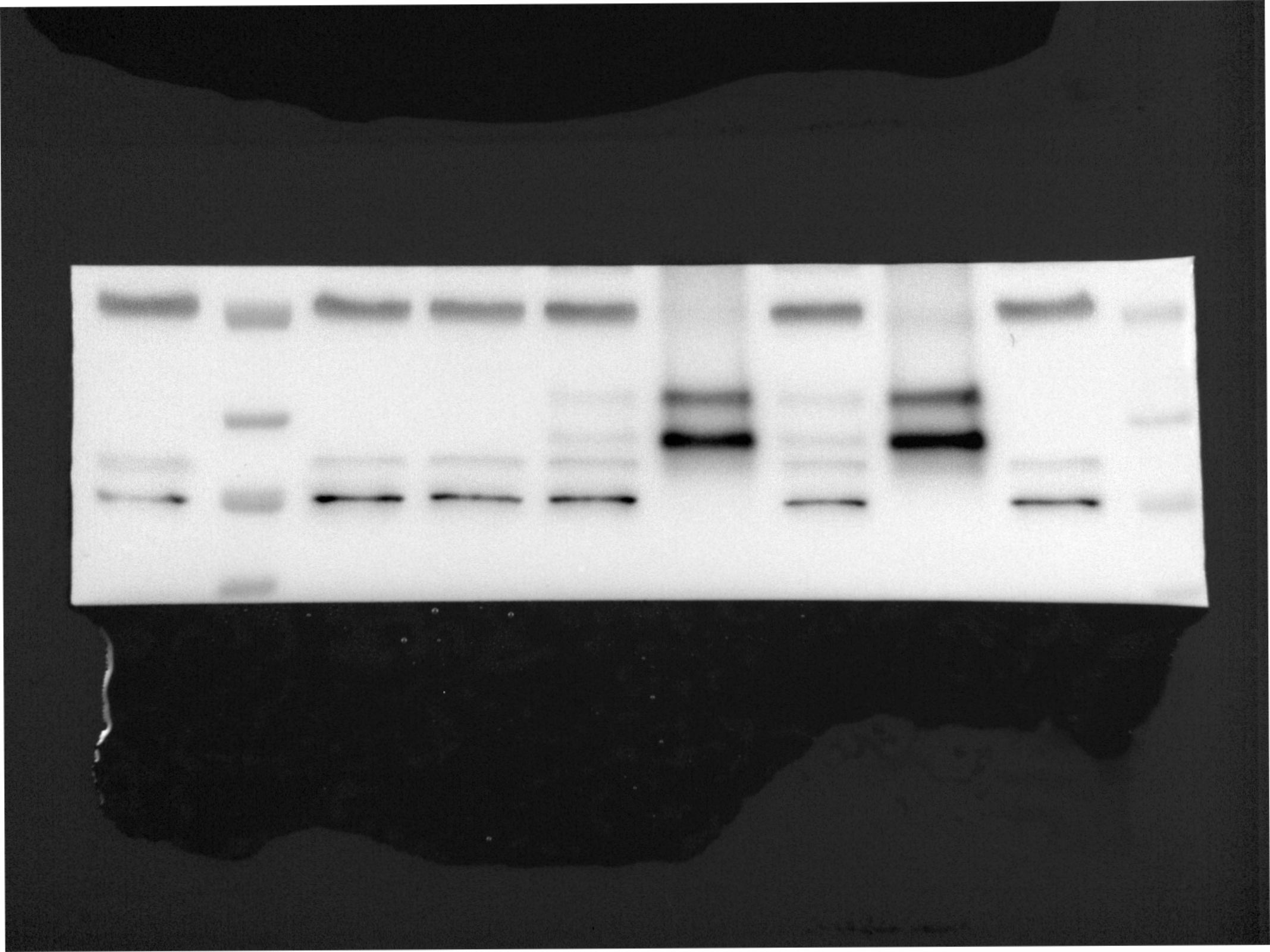

Supplement: Figure 1—figure supplement 1—source data 2. [file elife-89317-fig1-figsupp1-data2.zip › Original figures/GluT4 Original.jpg]

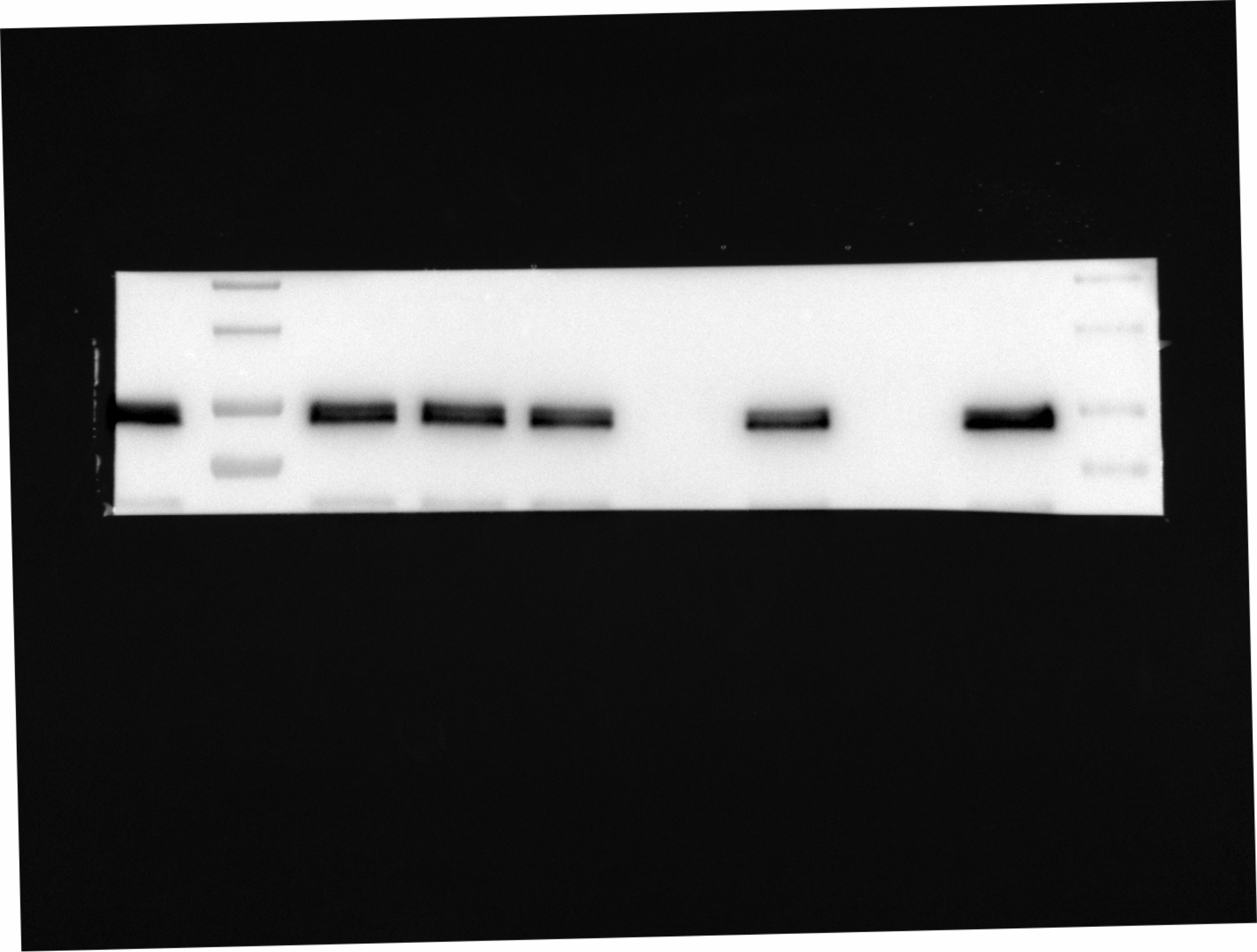

Supplement: Figure 1—figure supplement 1—source data 2. [file elife-89317-fig1-figsupp1-data2.zip › Original figures/GM130 Original.jpg]

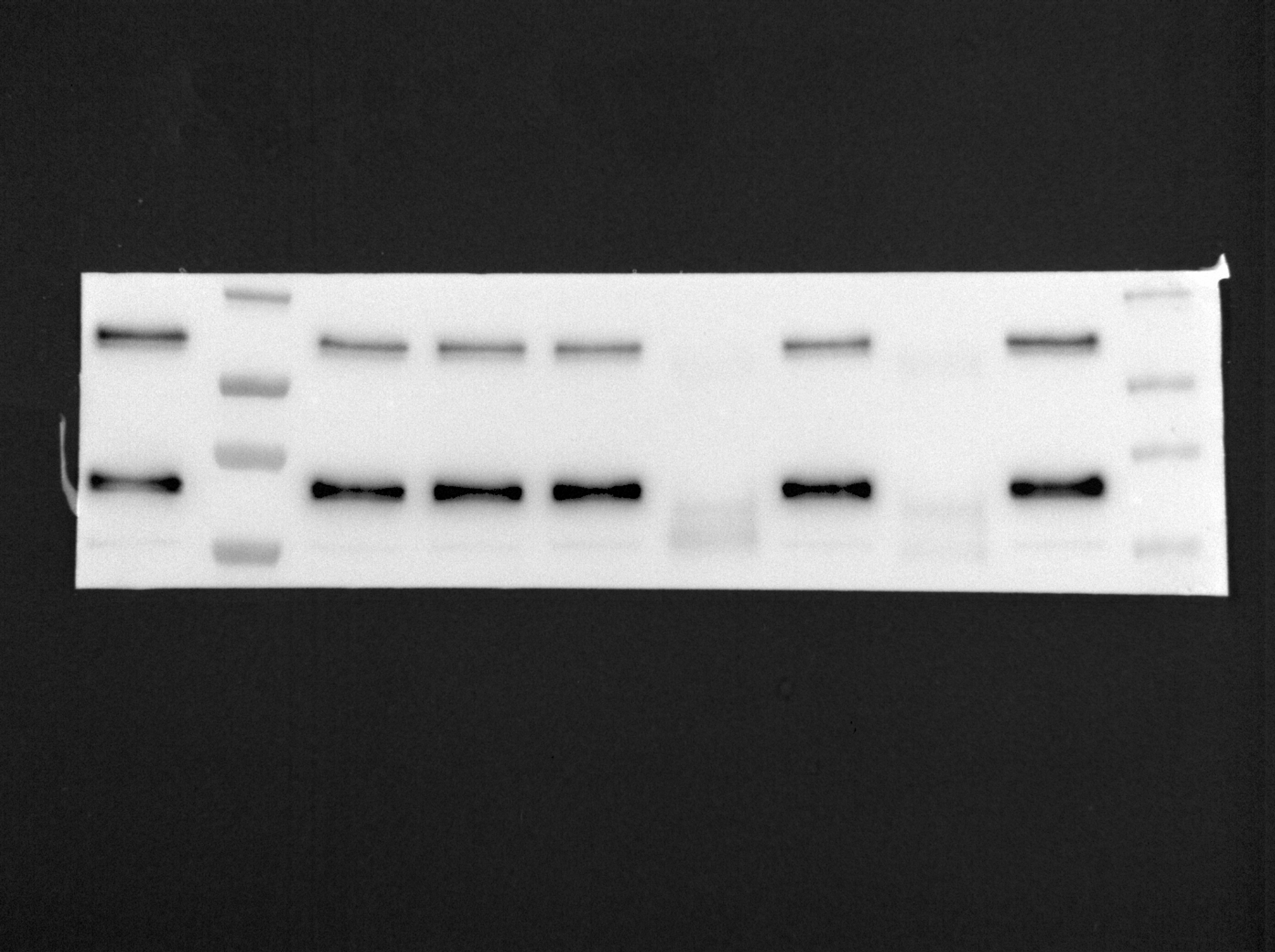

Supplement: Figure 1—figure supplement 1—source data 2. [file elife-89317-fig1-figsupp1-data2.zip › Original figures/golgi 97 Original.tif]

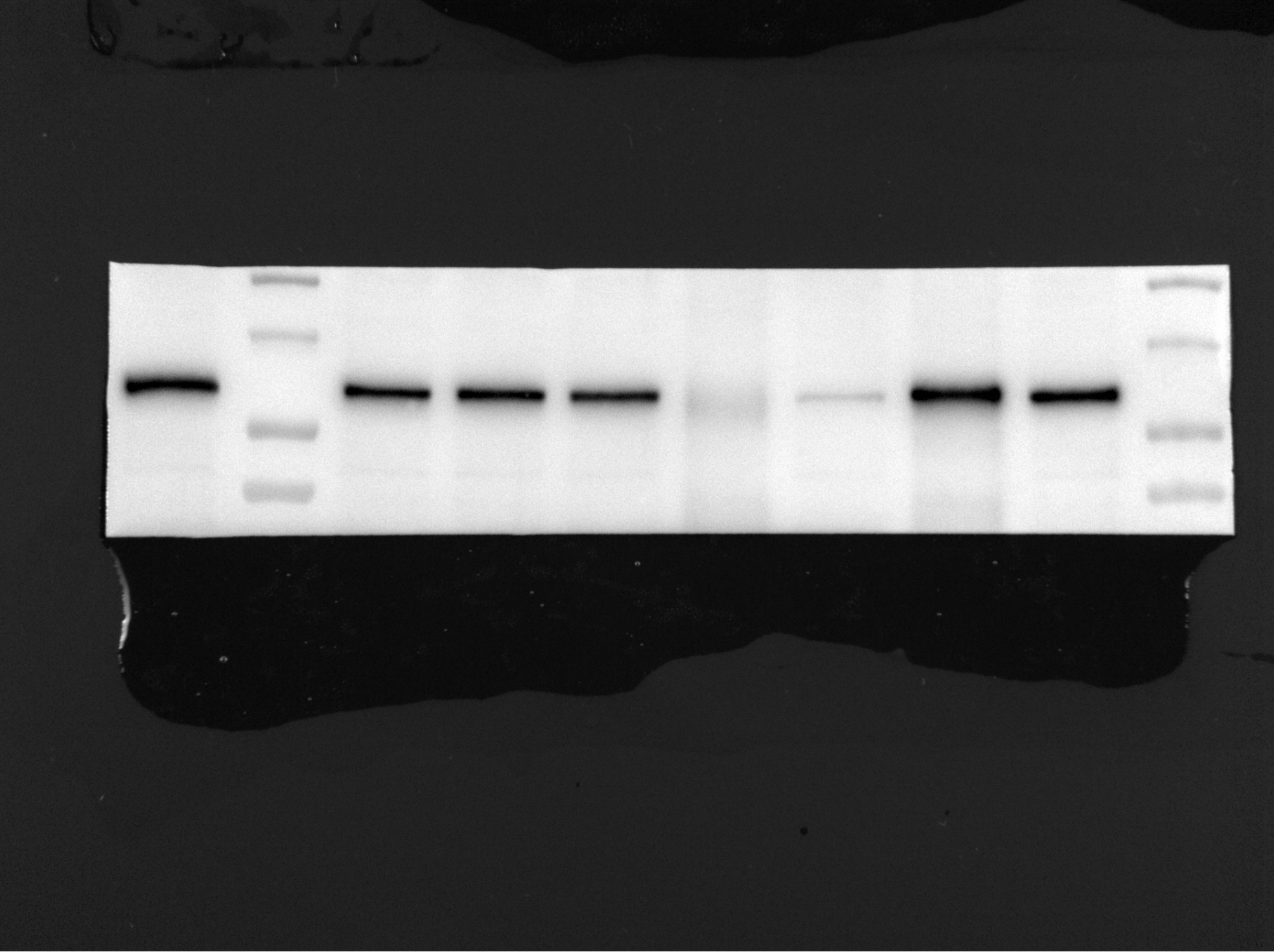

Supplement: Figure 1—figure supplement 1—source data 2. [file elife-89317-fig1-figsupp1-data2.zip › Original figures/H-ATPase Original.jpg]

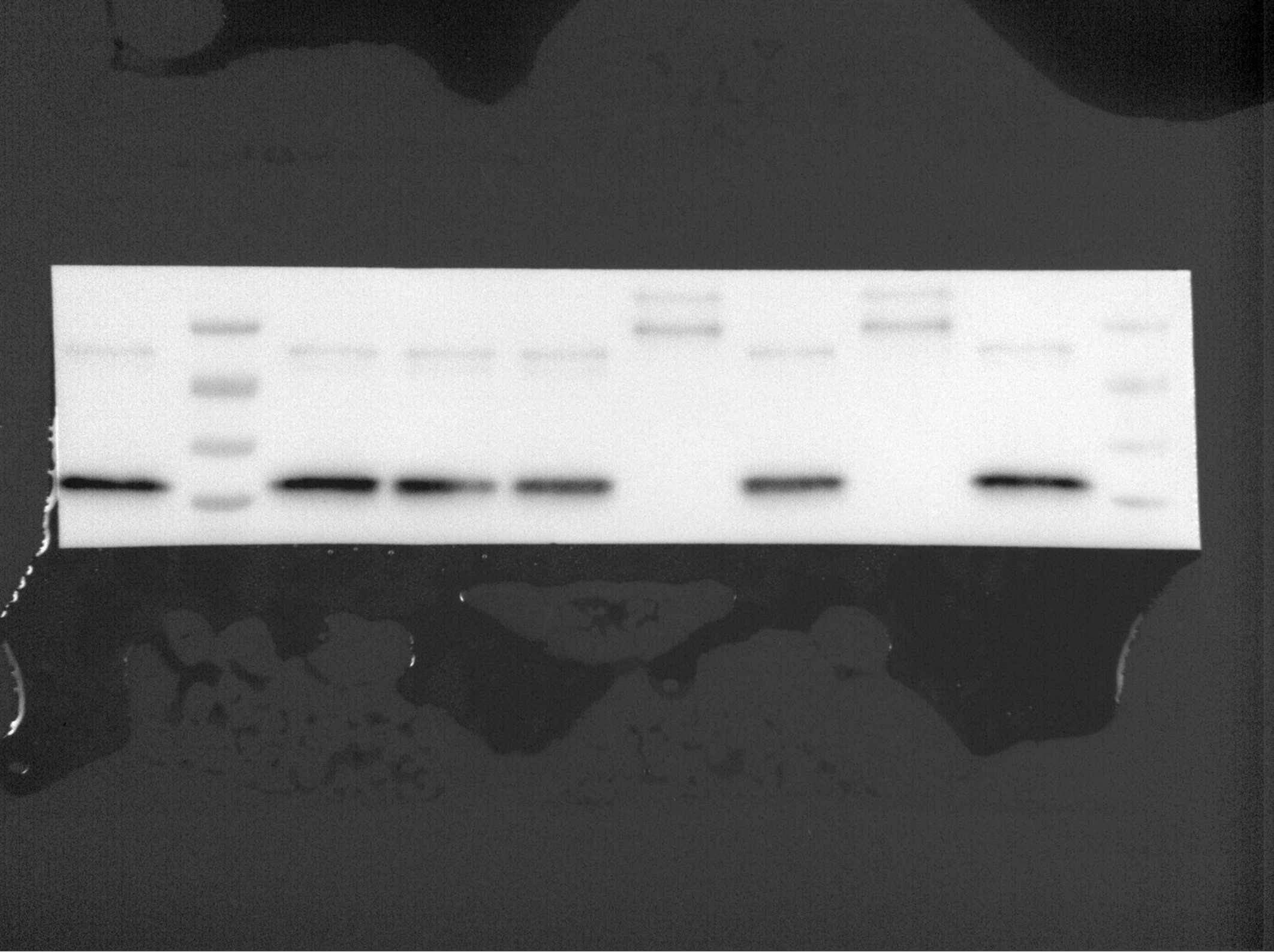

Supplement: Figure 1—figure supplement 1—source data 2. [file elife-89317-fig1-figsupp1-data2.zip › Original figures/LC3B Original.jpg]

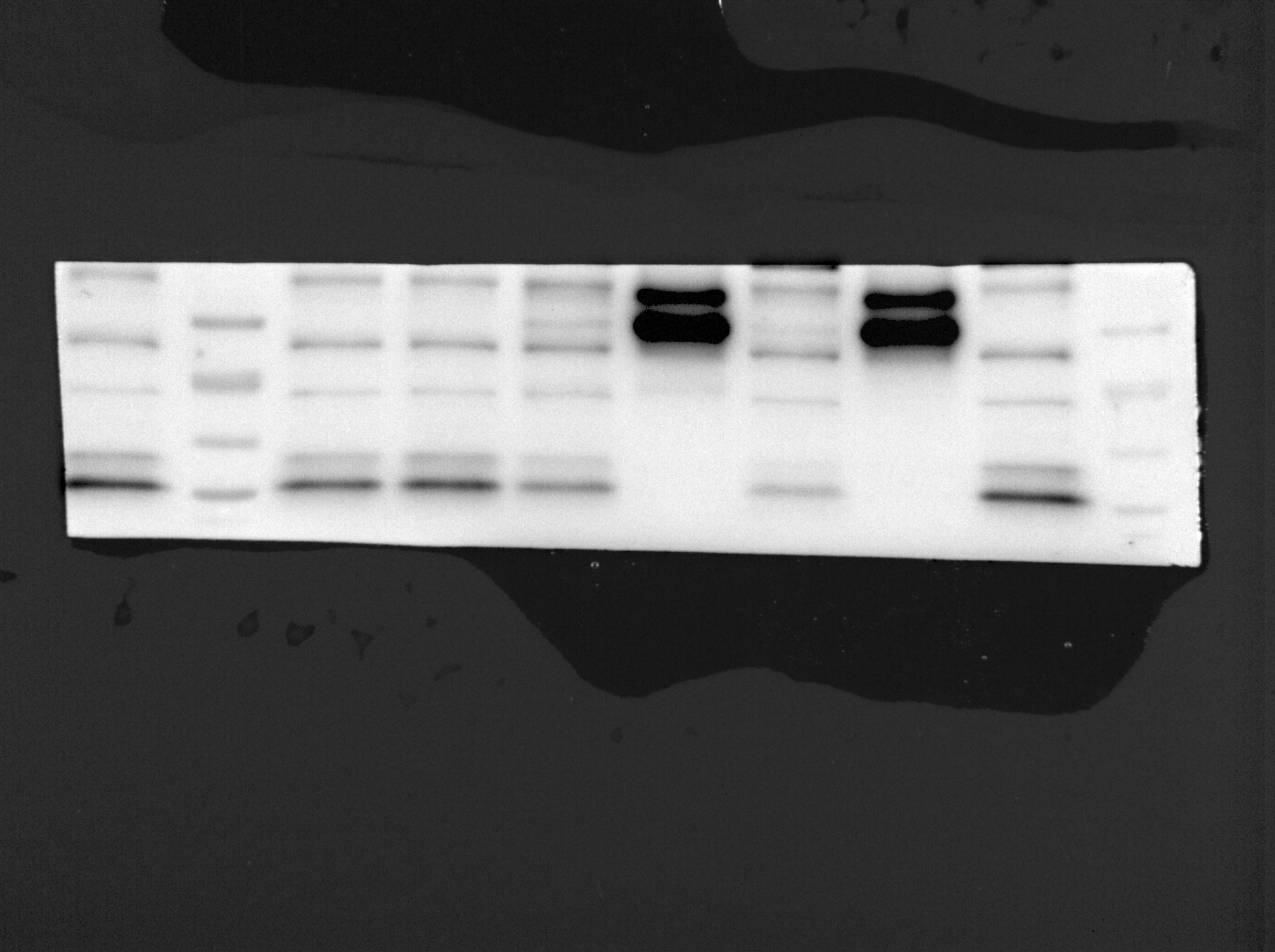

Supplement: Figure 1—figure supplement 1—source data 2. [file elife-89317-fig1-figsupp1-data2.zip › Original figures/MBP Original.tif]

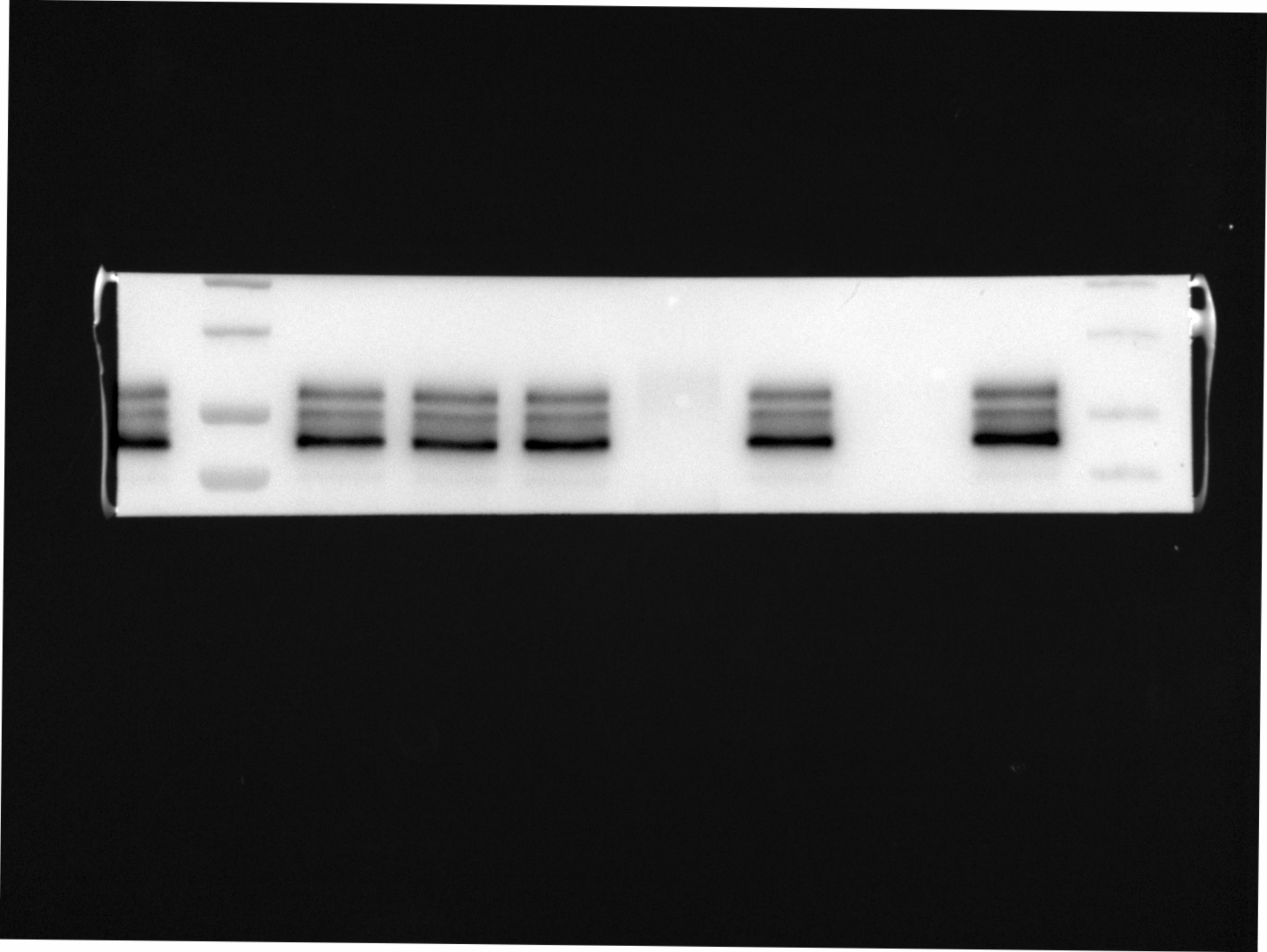

Supplement: Figure 1—figure supplement 1—source data 2. [file elife-89317-fig1-figsupp1-data2.zip › Original figures/PSD95 Original.jpg]

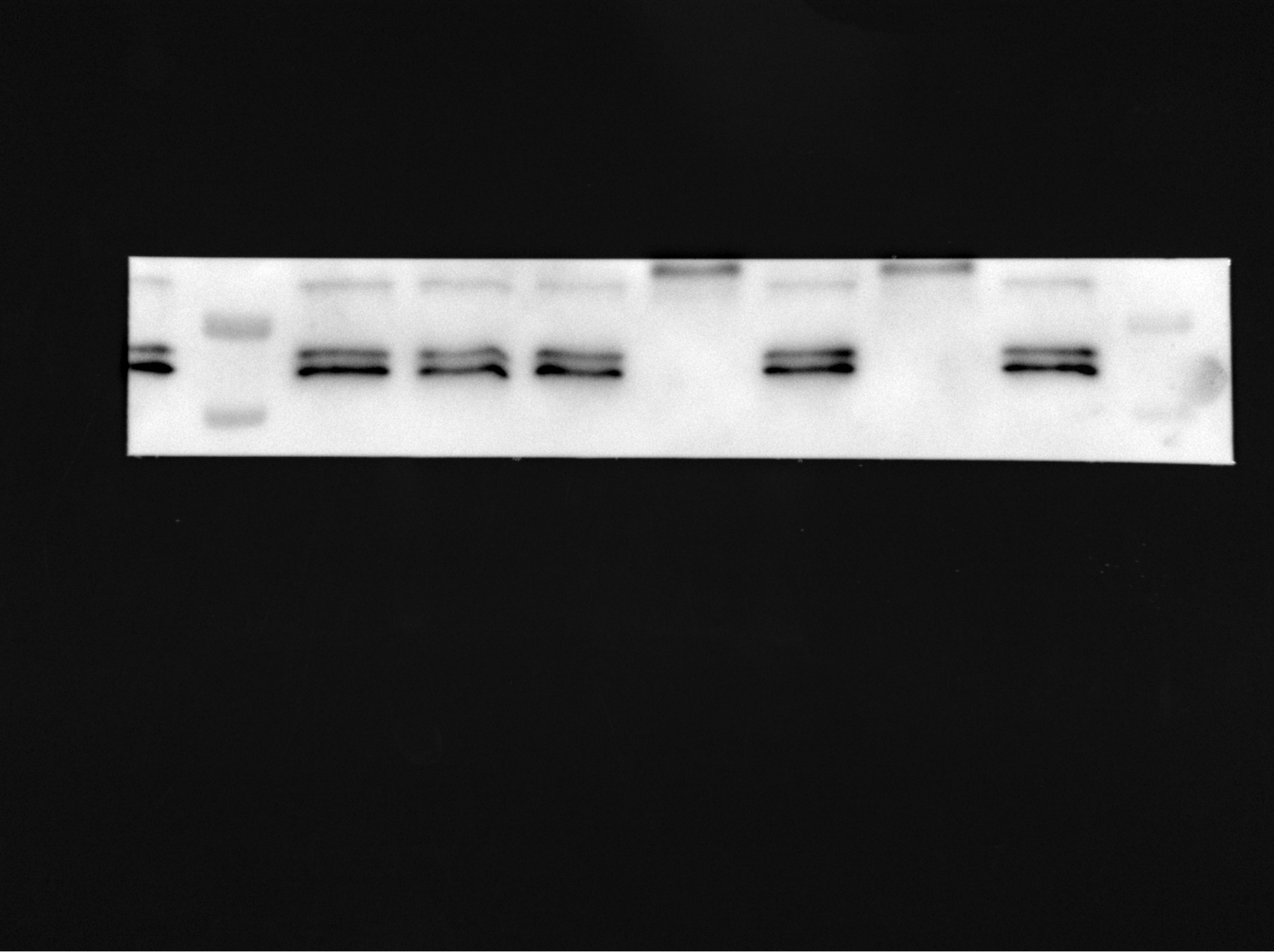

Supplement: Figure 1—figure supplement 1—source data 2. [file elife-89317-fig1-figsupp1-data2.zip › Original figures/SNAP23 Original.jpg]

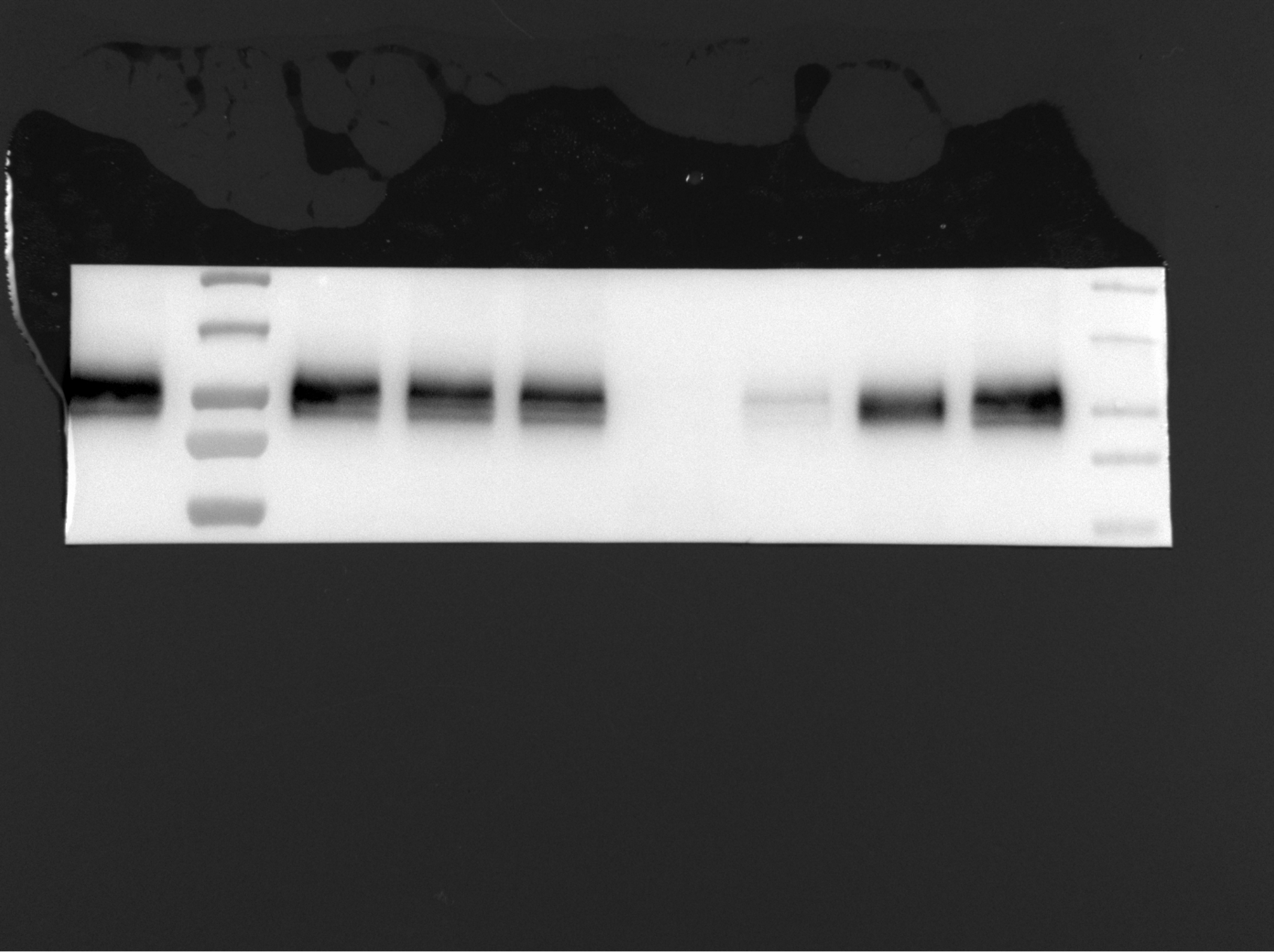

Supplement: Figure 1—figure supplement 1—source data 2. [file elife-89317-fig1-figsupp1-data2.zip › Original figures/SV2A original.jpg]

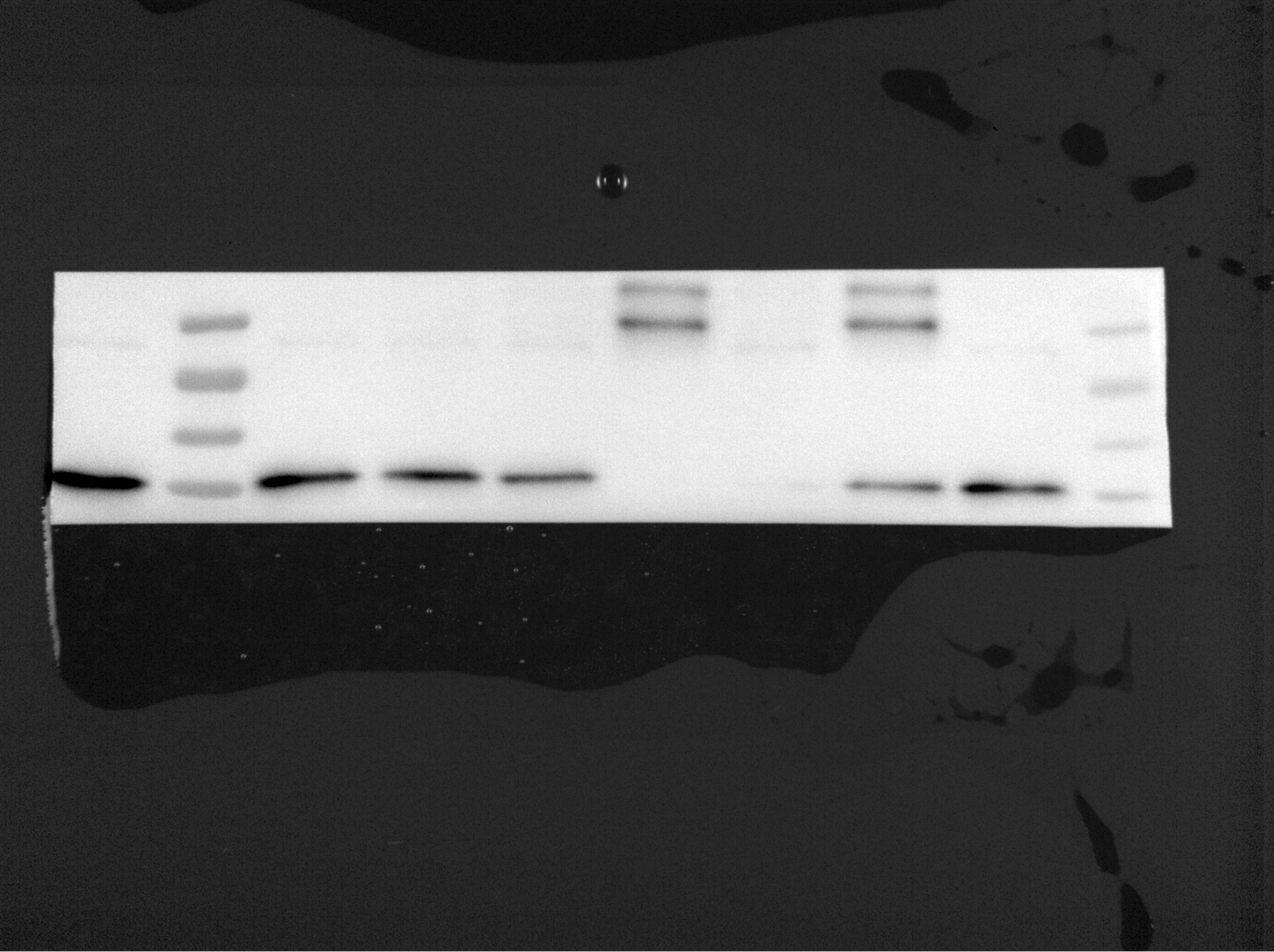

Supplement: Figure 1—figure supplement 1—source data 2. [file elife-89317-fig1-figsupp1-data2.zip › Original figures/SYB2 Original.jpg]

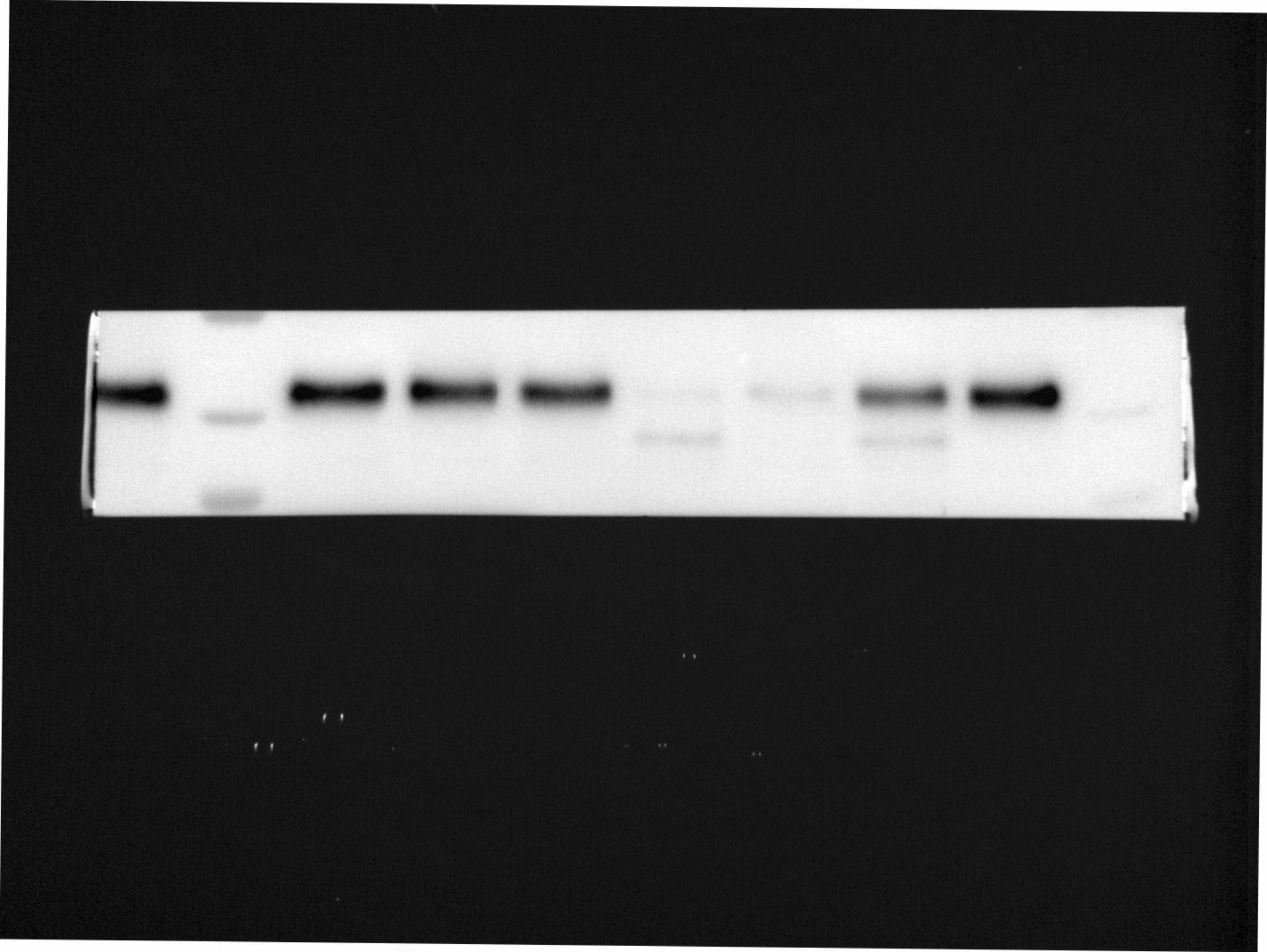

Supplement: Figure 1—figure supplement 1—source data 2. [file elife-89317-fig1-figsupp1-data2.zip › Original figures/Syp original.jpg]

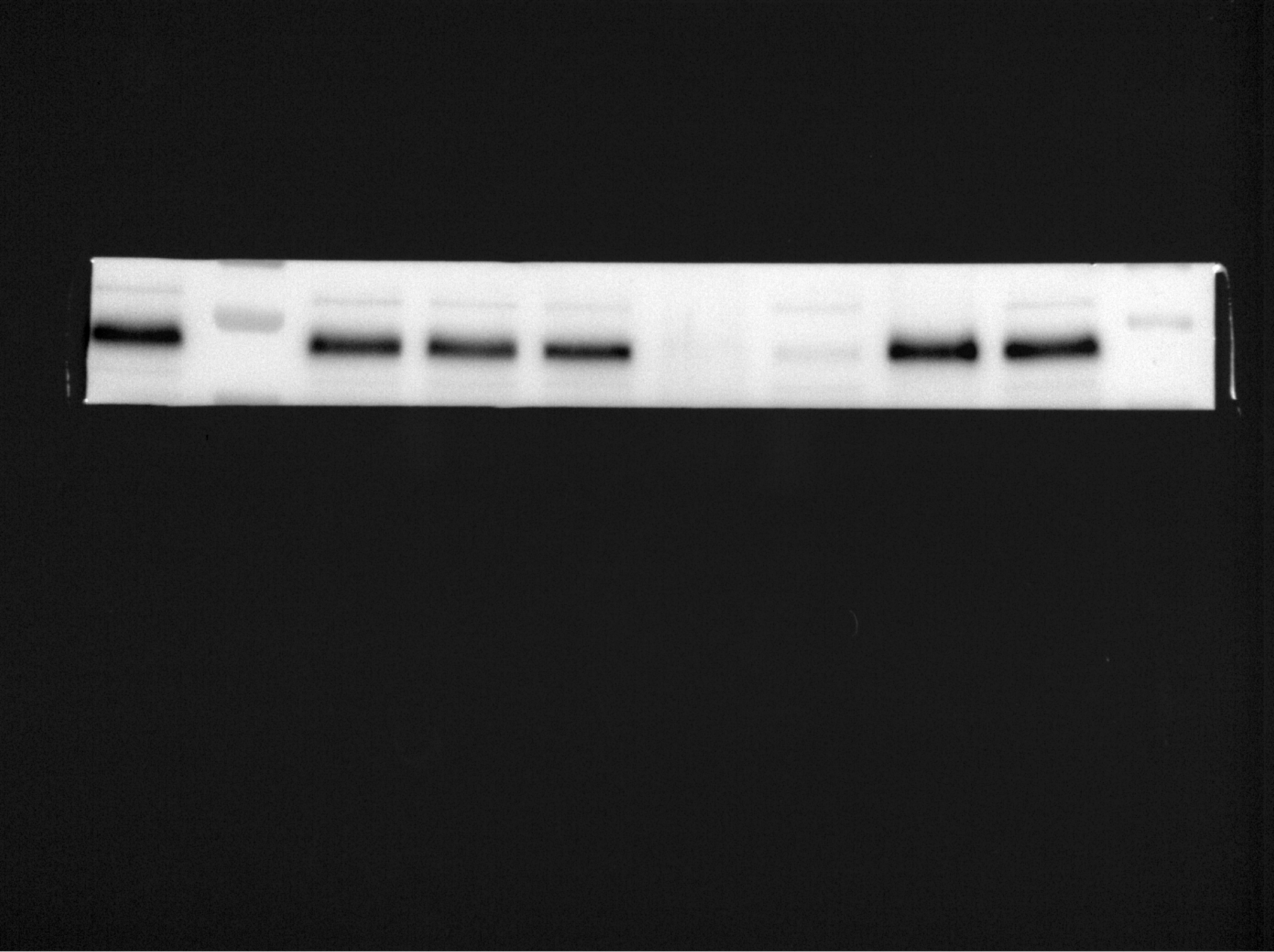

Supplement: Figure 1—figure supplement 1—source data 2. [file elife-89317-fig1-figsupp1-data2.zip › Original figures/Syt original.jpg]

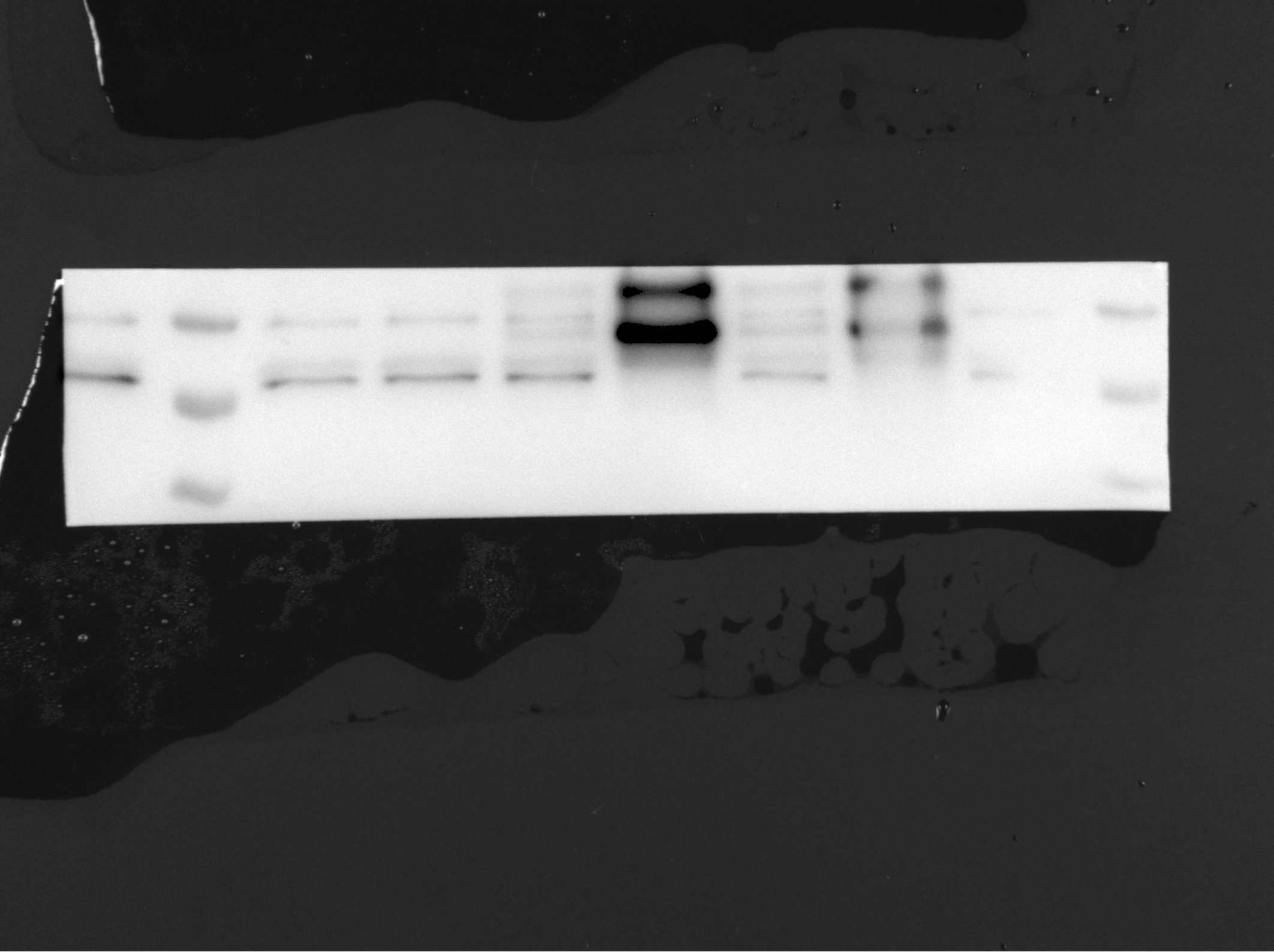

Supplement: Figure 1—figure supplement 1—source data 2. [file elife-89317-fig1-figsupp1-data2.zip › Original figures/VDAC Original.jpg]

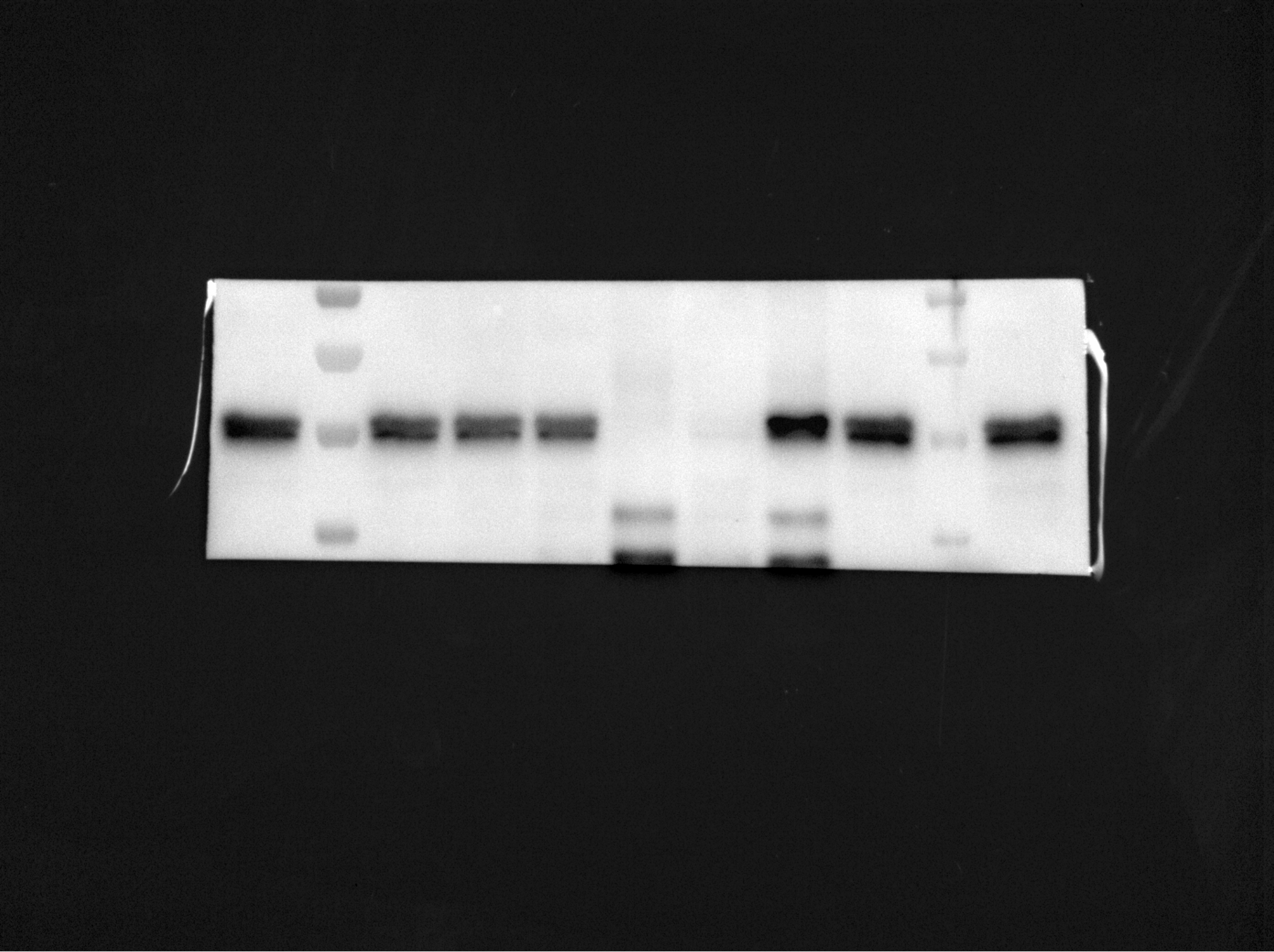

Supplement: Figure 1—figure supplement 1—source data 2. [file elife-89317-fig1-figsupp1-data2.zip › Original figures/VGAT Original.jpg]

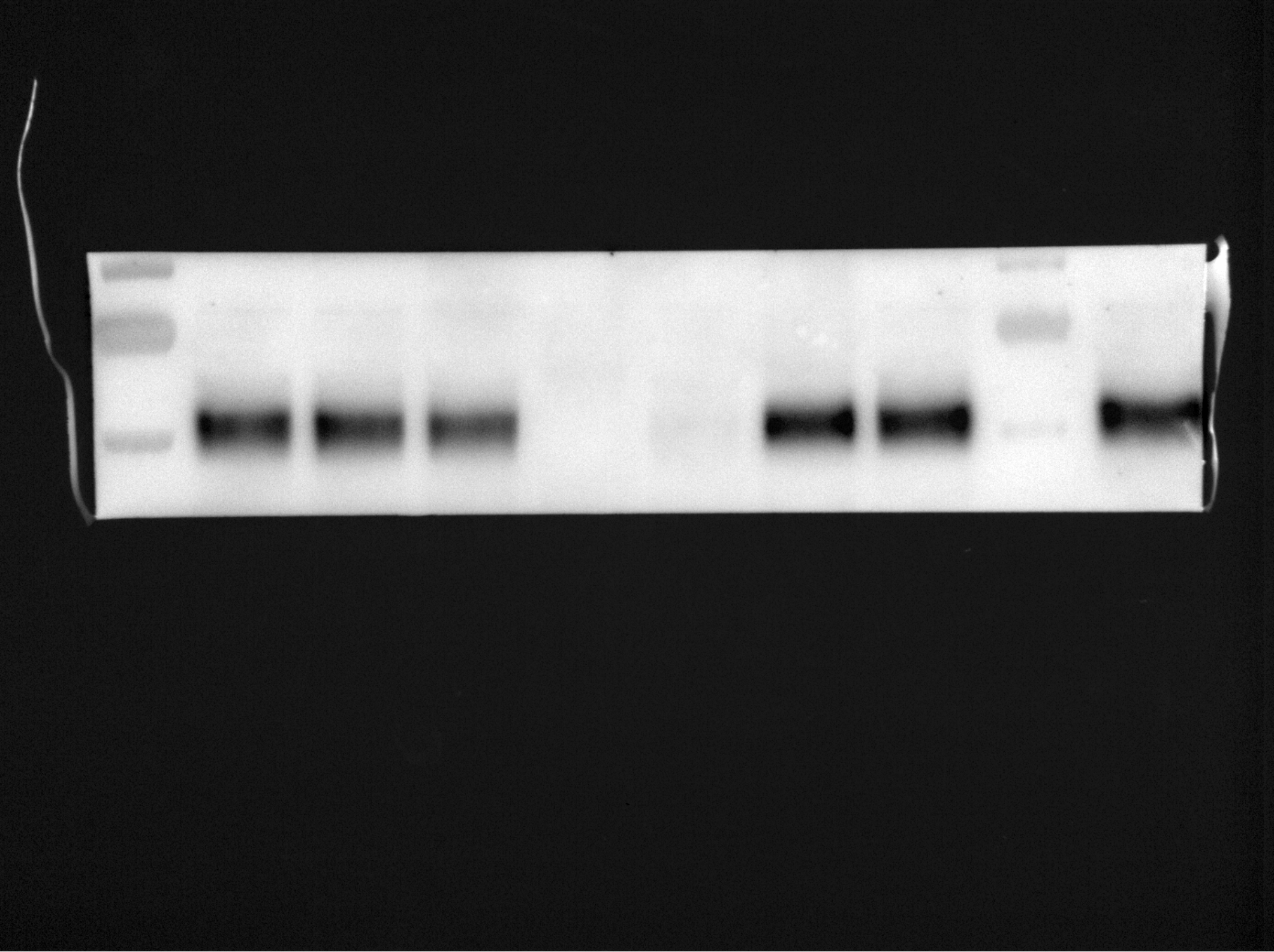

Supplement: Figure 1—figure supplement 1—source data 2. [file elife-89317-fig1-figsupp1-data2.zip › Original figures/VGlut1 Original.jpg]

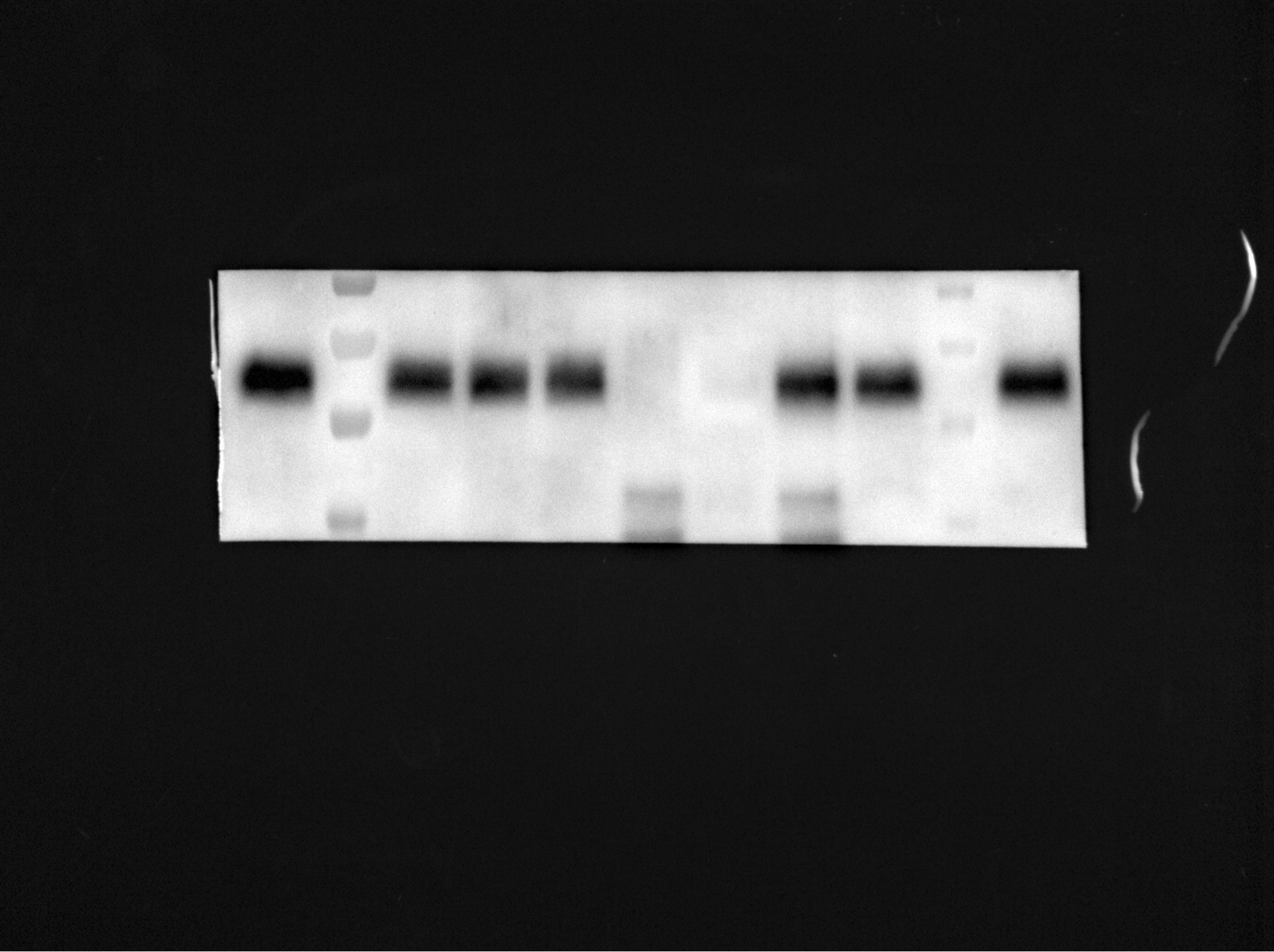

Supplement: Figure 1—figure supplement 1—source data 2. [file elife-89317-fig1-figsupp1-data2.zip › Original figures/vGLUT2 Original.jpg]

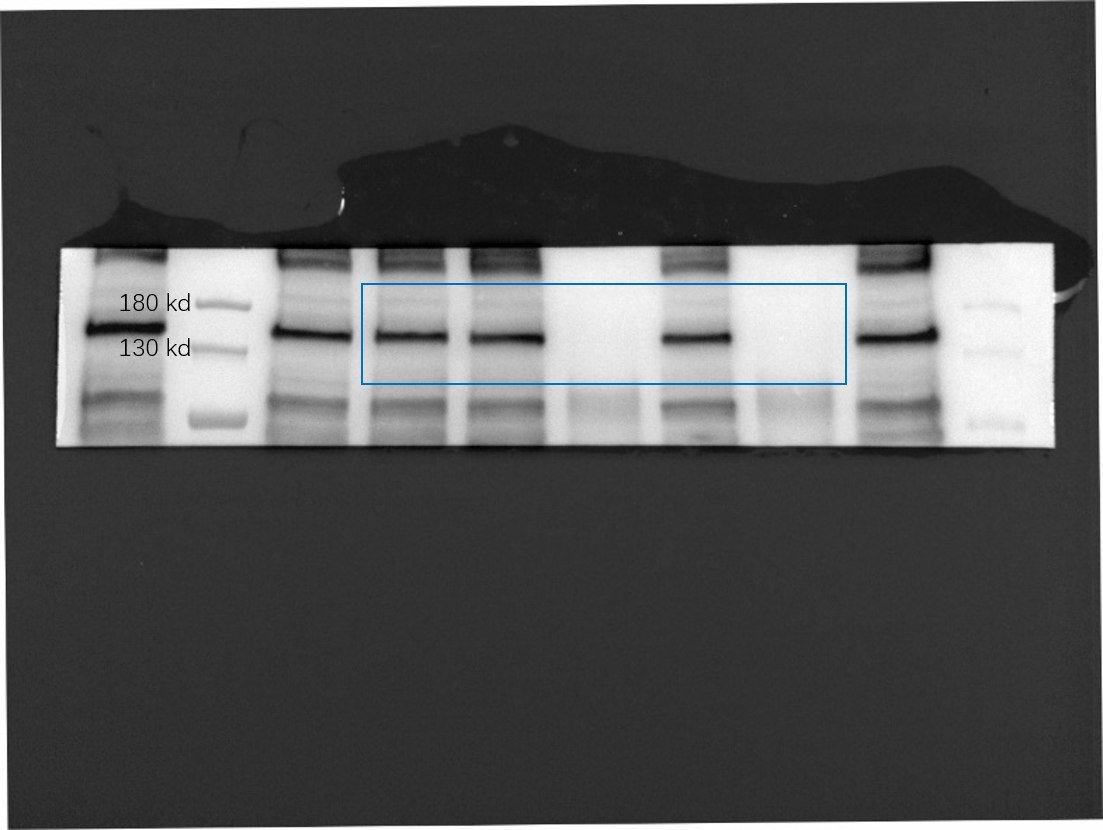

Supplement: Figure 1—figure supplement 1—source data 3. [file elife-89317-fig1-figsupp1-data3.zip › Labelled figures/CACNA1A labelled.png]

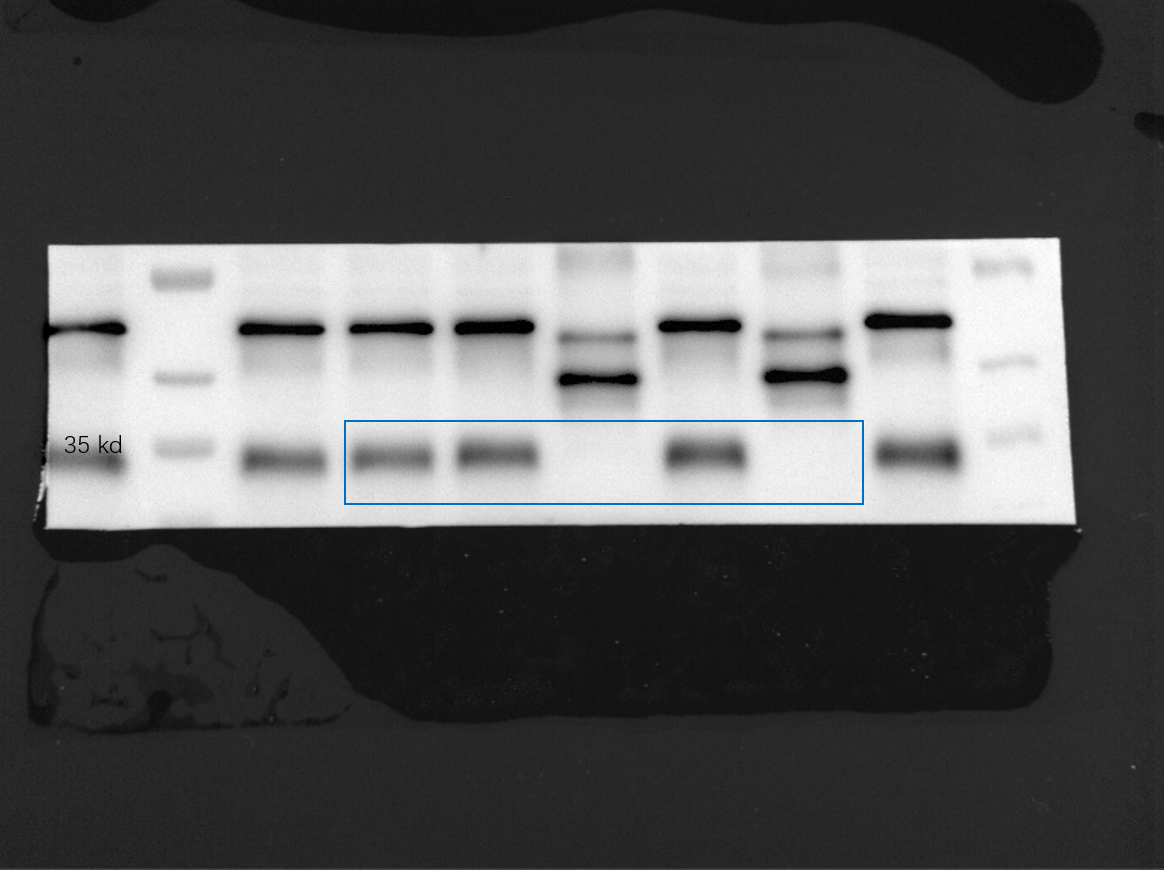

Supplement: Figure 1—figure supplement 1—source data 3. [file elife-89317-fig1-figsupp1-data3.zip › Labelled figures/CathepsinB labelled.png]

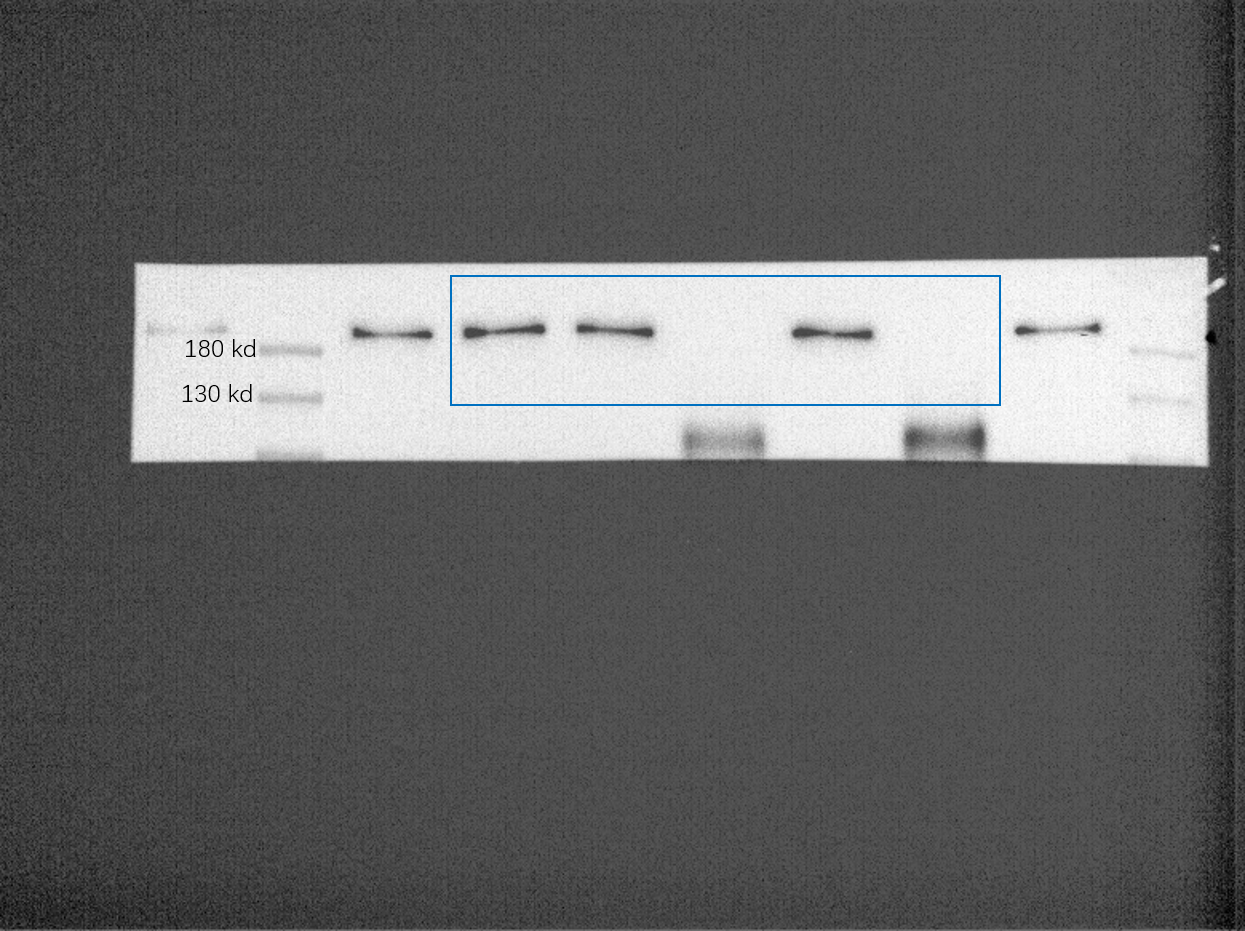

Supplement: Figure 1—figure supplement 1—source data 3. [file elife-89317-fig1-figsupp1-data3.zip › Labelled figures/EEA1 labelled.png]

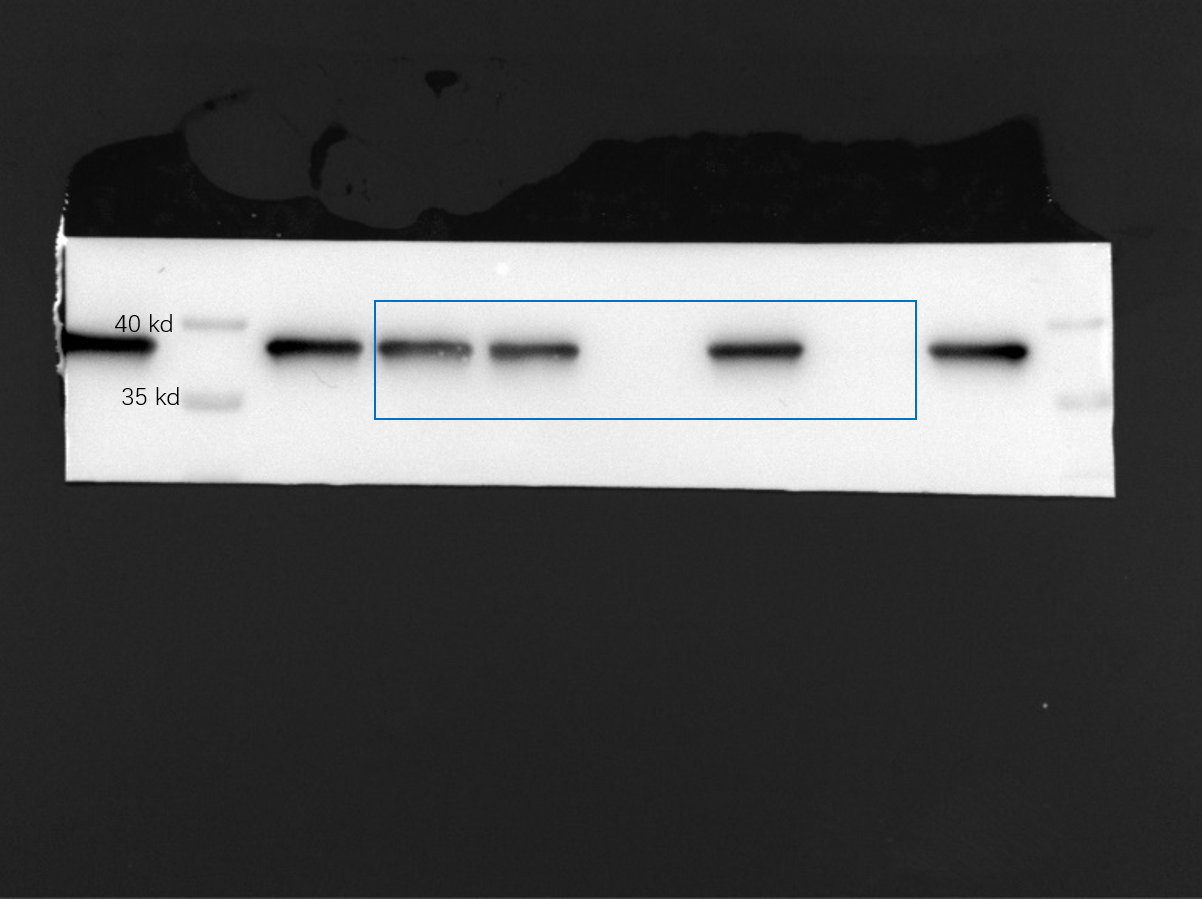

Supplement: Figure 1—figure supplement 1—source data 3. [file elife-89317-fig1-figsupp1-data3.zip › Labelled figures/GAPDH labelled.png]

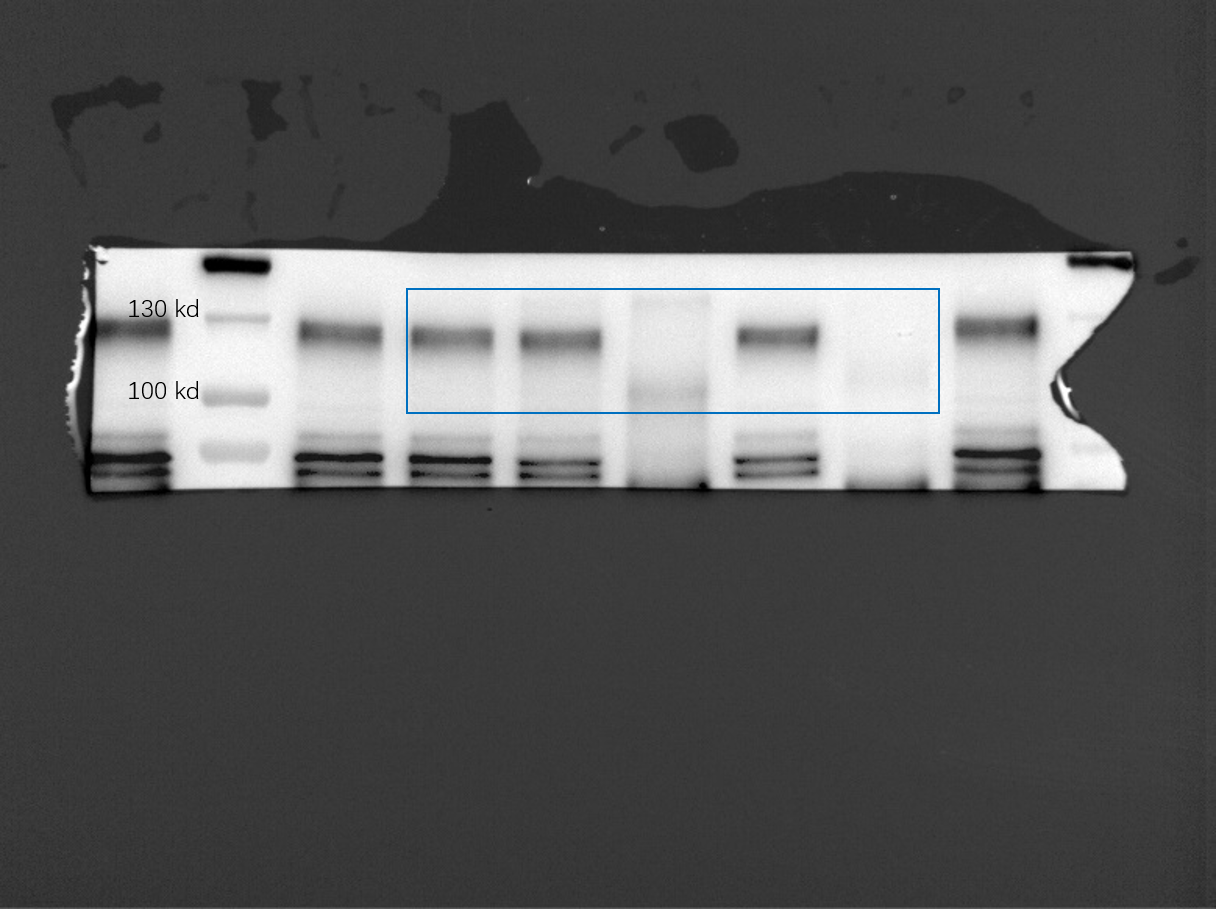

Supplement: Figure 1—figure supplement 1—source data 3. [file elife-89317-fig1-figsupp1-data3.zip › Labelled figures/GluN1 labelled.png]

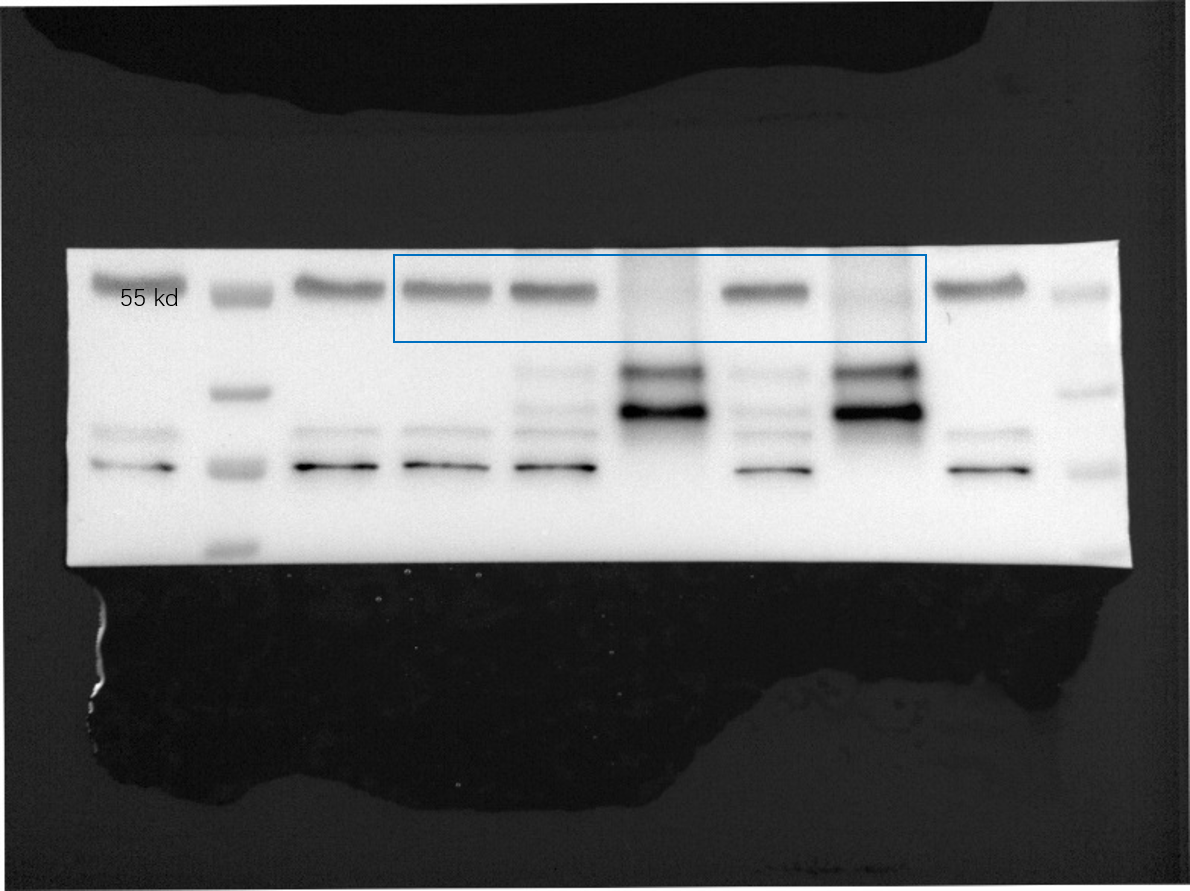

Supplement: Figure 1—figure supplement 1—source data 3. [file elife-89317-fig1-figsupp1-data3.zip › Labelled figures/GluT4 labelled.png]

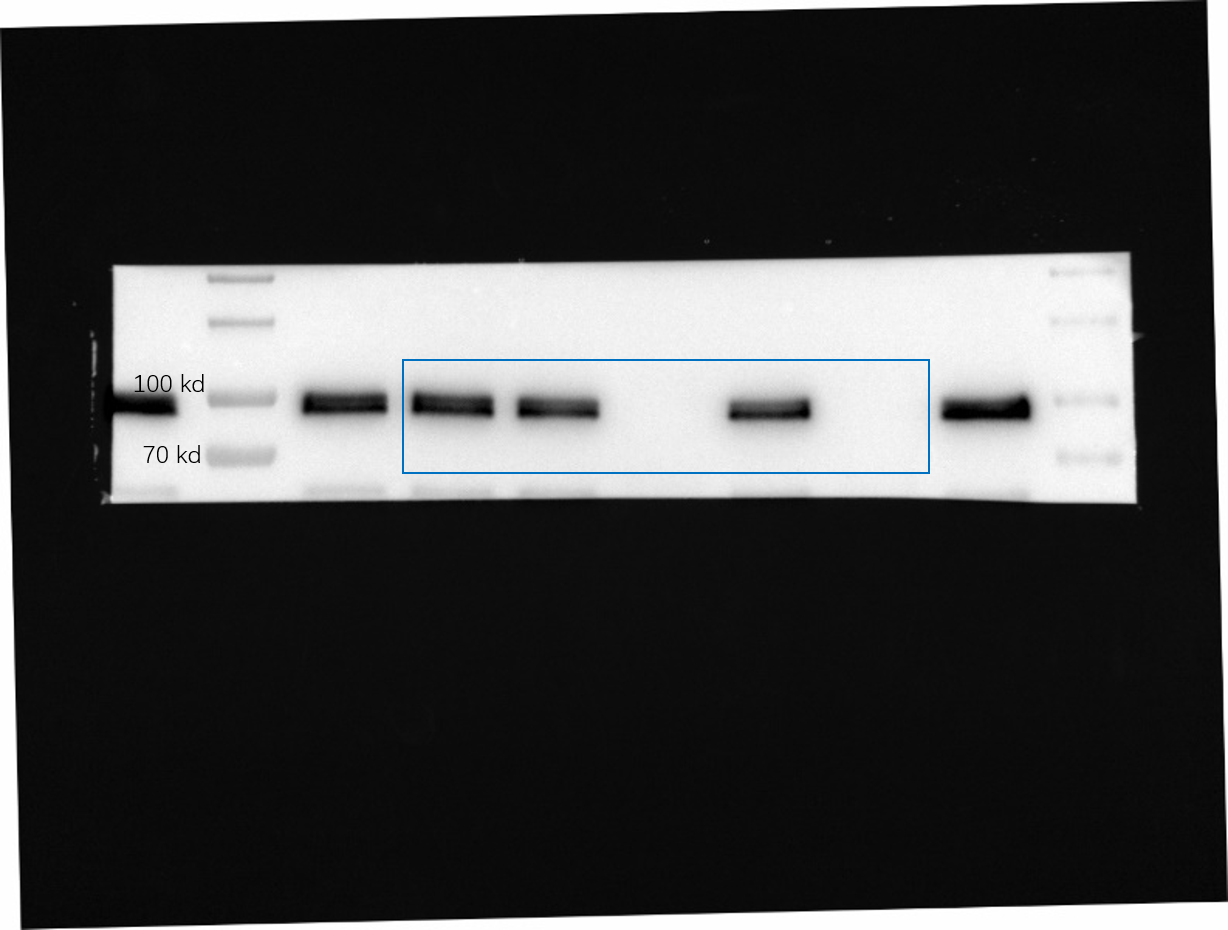

Supplement: Figure 1—figure supplement 1—source data 3. [file elife-89317-fig1-figsupp1-data3.zip › Labelled figures/GM130 labelled.png]

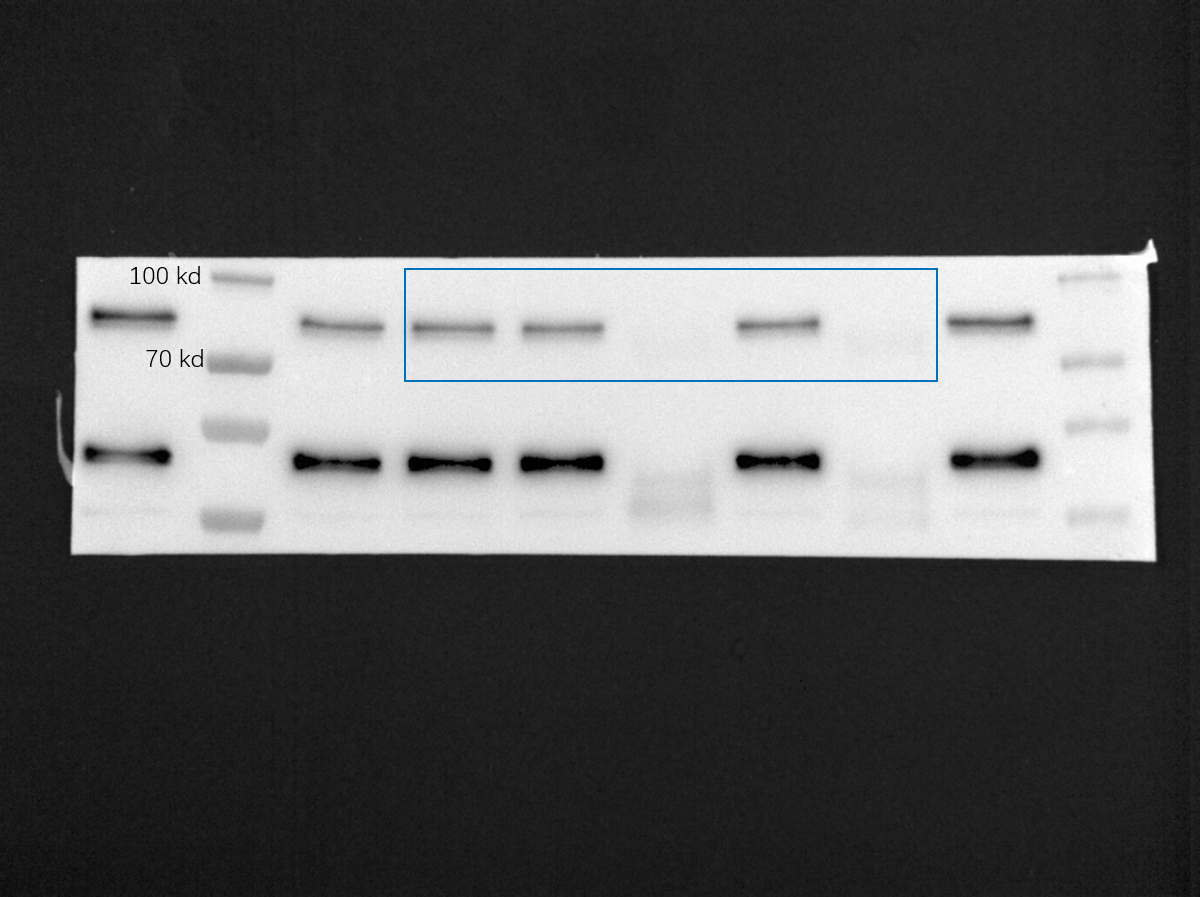

Supplement: Figure 1—figure supplement 1—source data 3. [file elife-89317-fig1-figsupp1-data3.zip › Labelled figures/Golgin97 labelled.png]

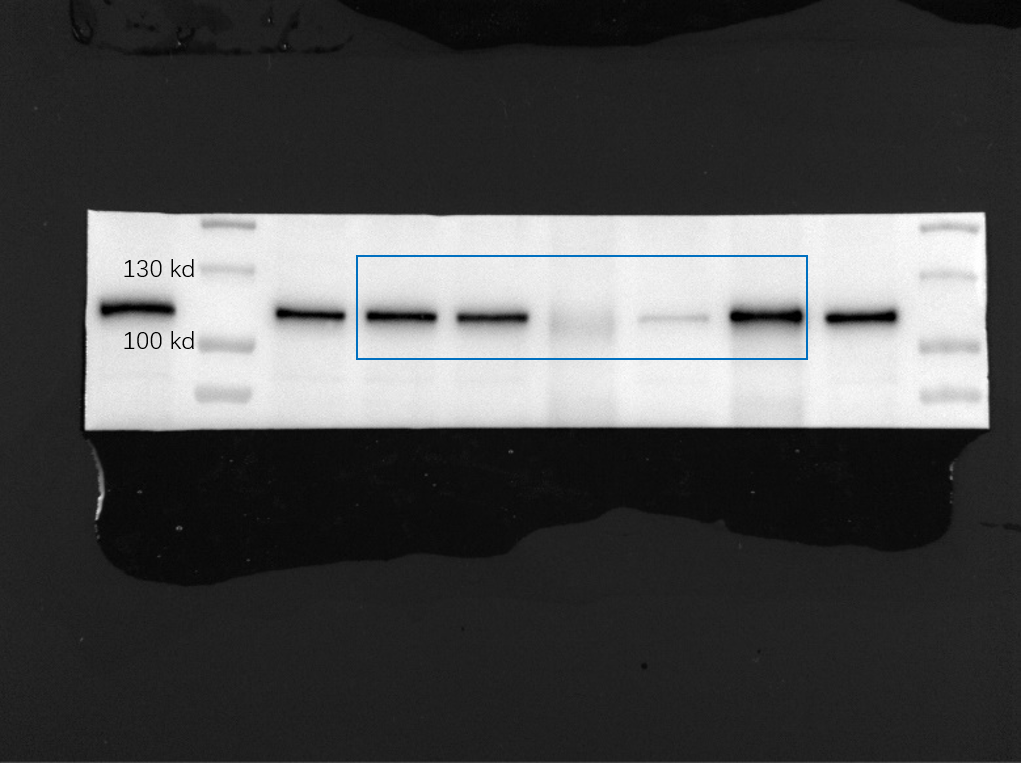

Supplement: Figure 1—figure supplement 1—source data 3. [file elife-89317-fig1-figsupp1-data3.zip › Labelled figures/H-ATPase labelled.png]

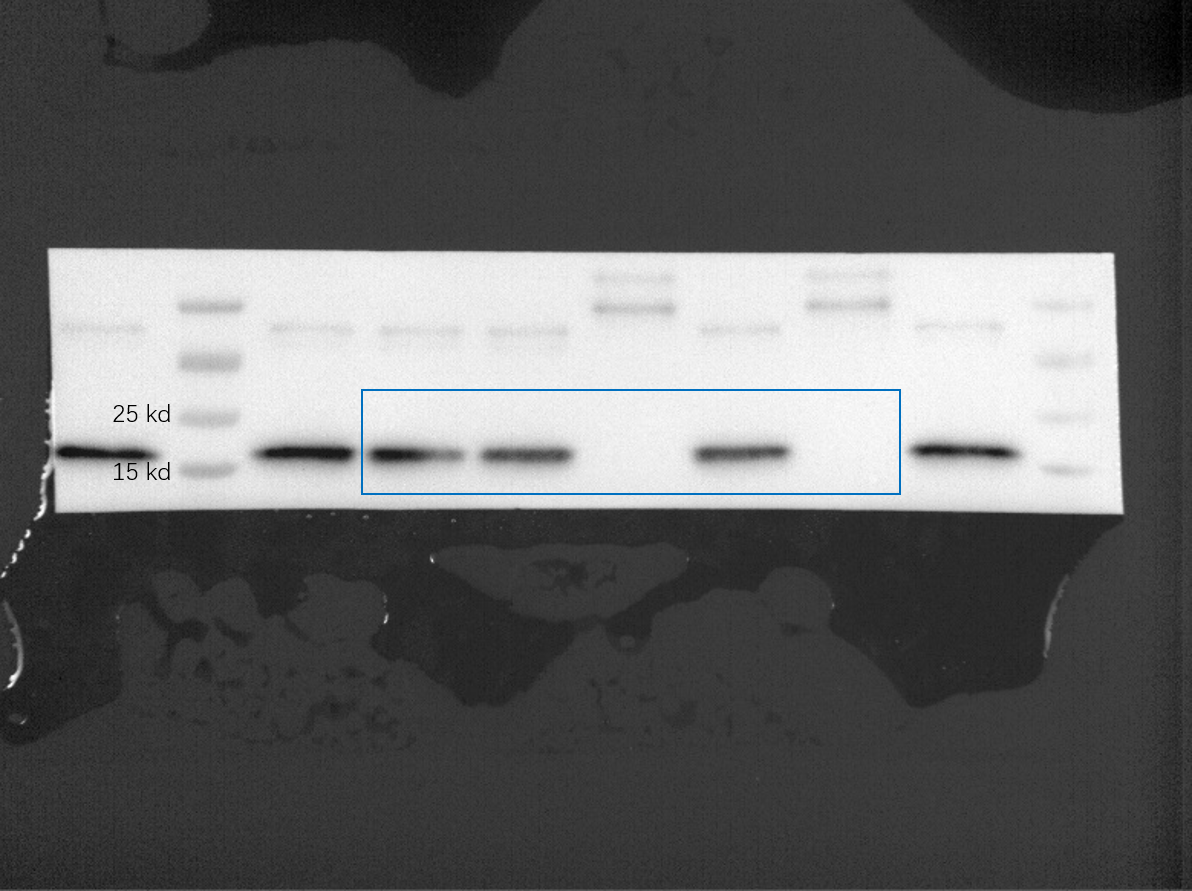

Supplement: Figure 1—figure supplement 1—source data 3. [file elife-89317-fig1-figsupp1-data3.zip › Labelled figures/LC3B labelled.png]

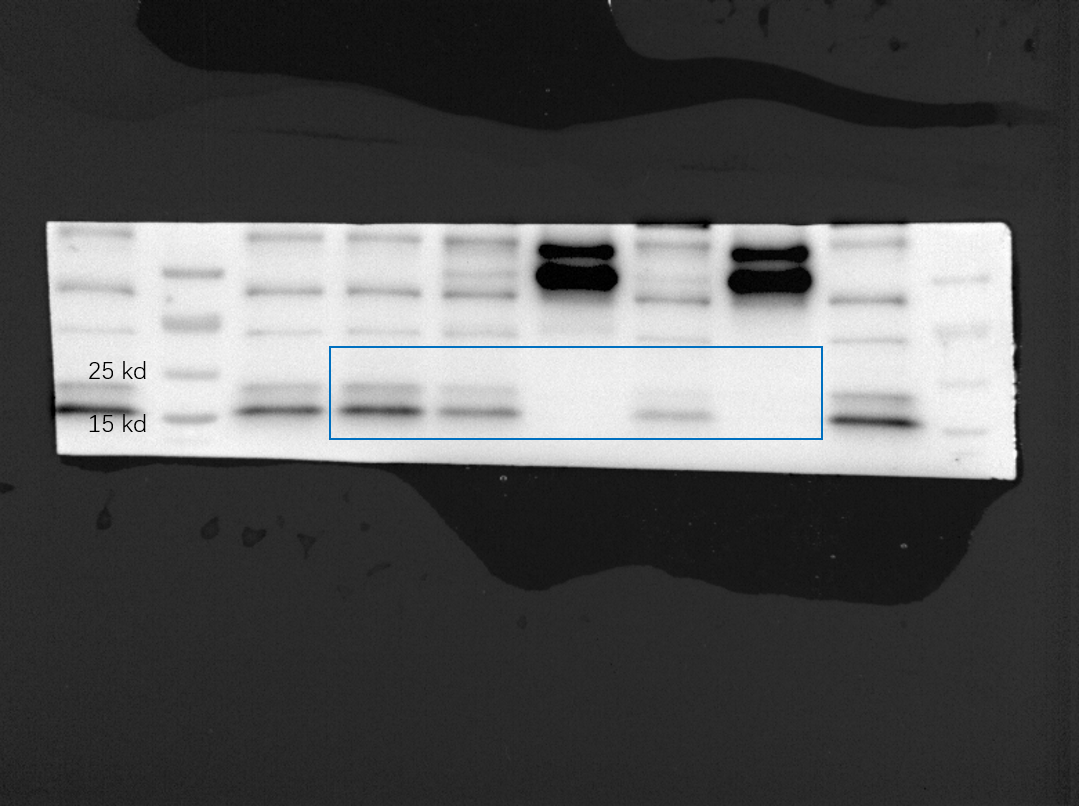

Supplement: Figure 1—figure supplement 1—source data 3. [file elife-89317-fig1-figsupp1-data3.zip › Labelled figures/MBP labelled.png]

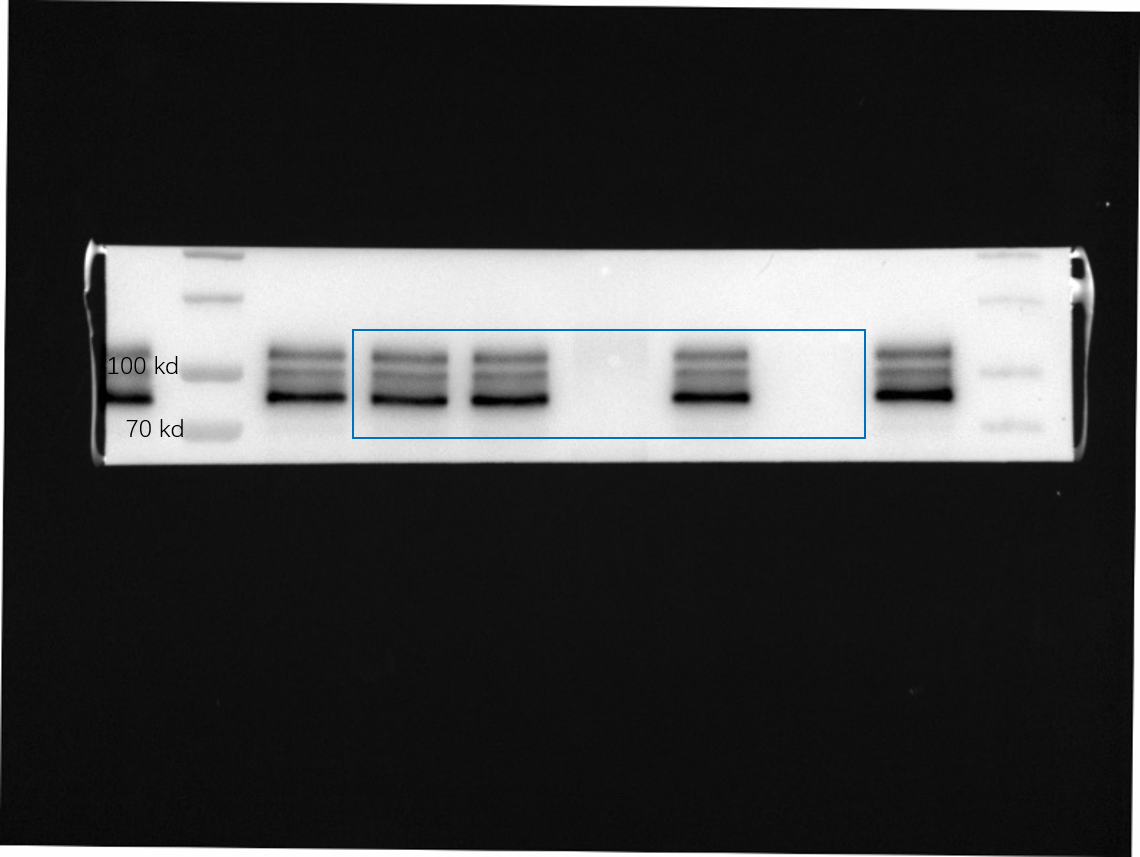

Supplement: Figure 1—figure supplement 1—source data 3. [file elife-89317-fig1-figsupp1-data3.zip › Labelled figures/PSD95 labelled.png]

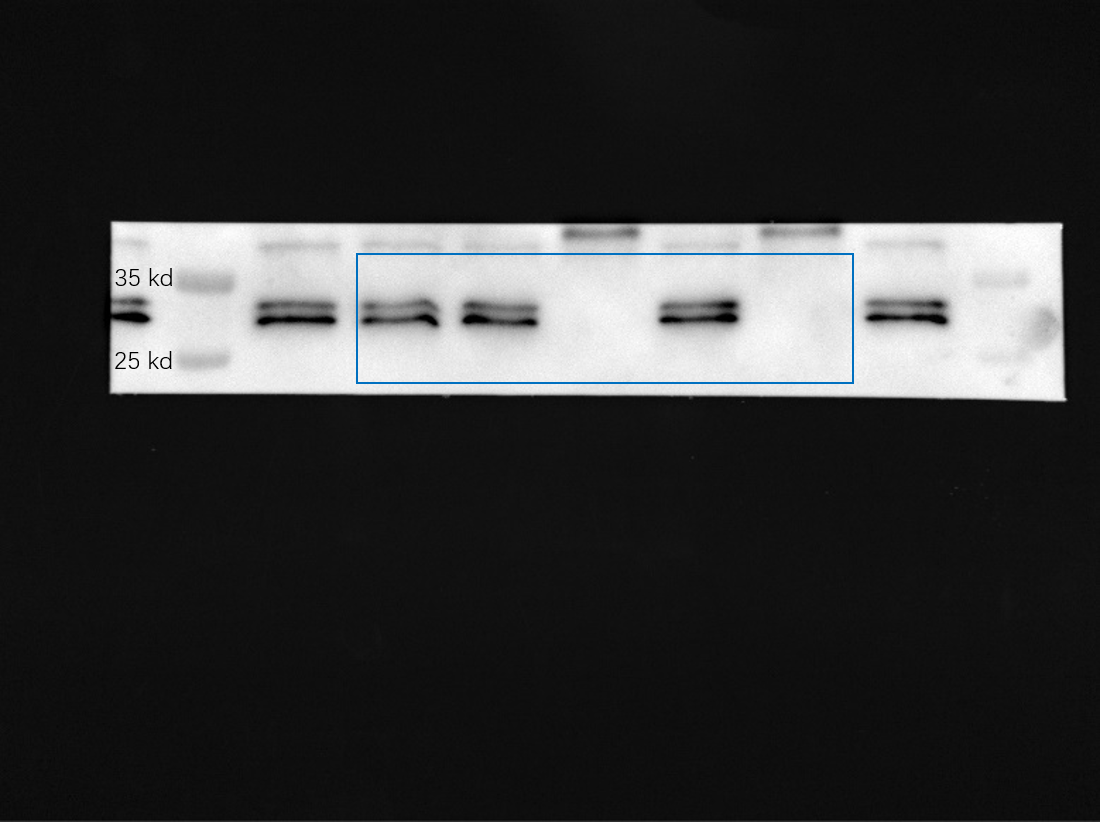

Supplement: Figure 1—figure supplement 1—source data 3. [file elife-89317-fig1-figsupp1-data3.zip › Labelled figures/SNAP23 labelled.png]

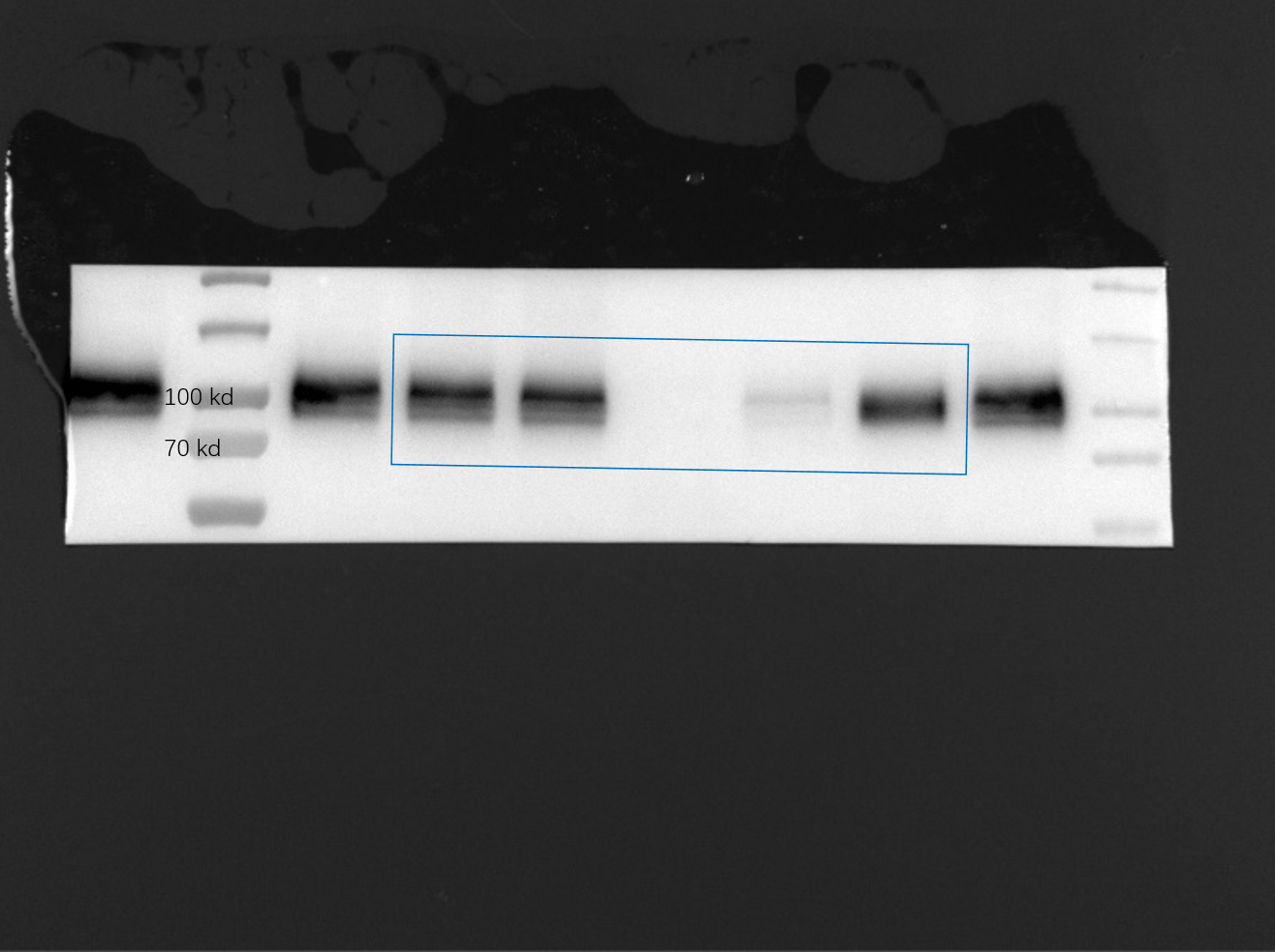

Supplement: Figure 1—figure supplement 1—source data 3. [file elife-89317-fig1-figsupp1-data3.zip › Labelled figures/SV2A labelled.png]

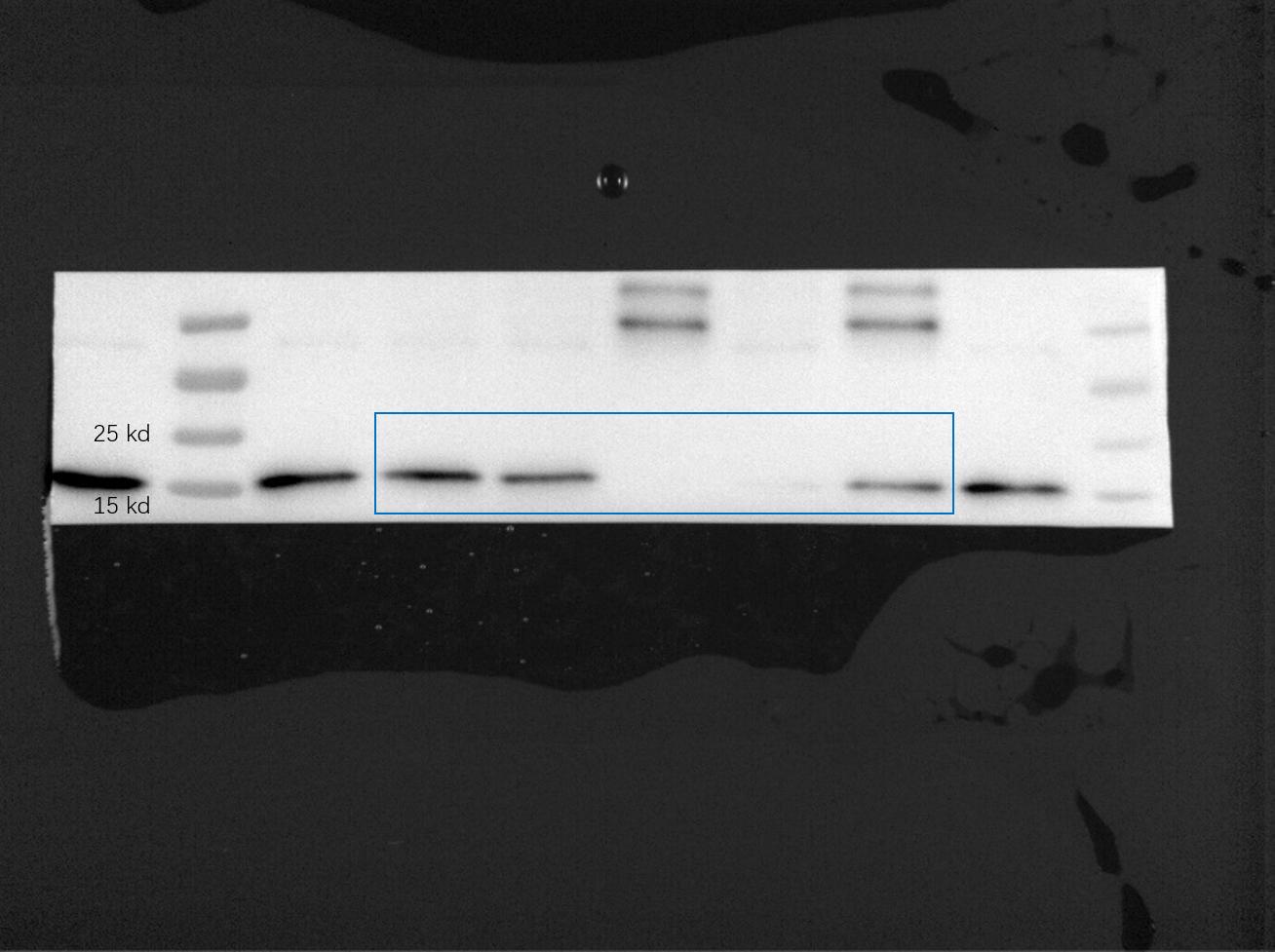

Supplement: Figure 1—figure supplement 1—source data 3. [file elife-89317-fig1-figsupp1-data3.zip › Labelled figures/syb2 labelled.png]

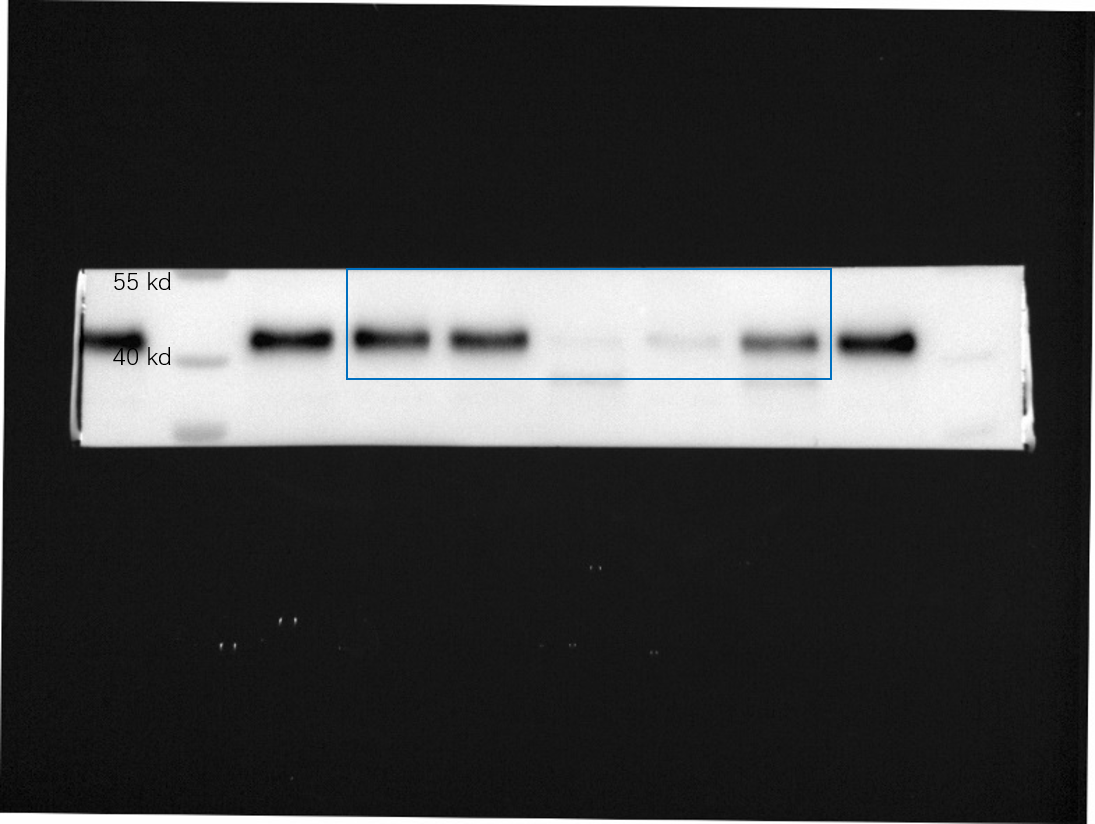

Supplement: Figure 1—figure supplement 1—source data 3. [file elife-89317-fig1-figsupp1-data3.zip › Labelled figures/Syp labelled.png]

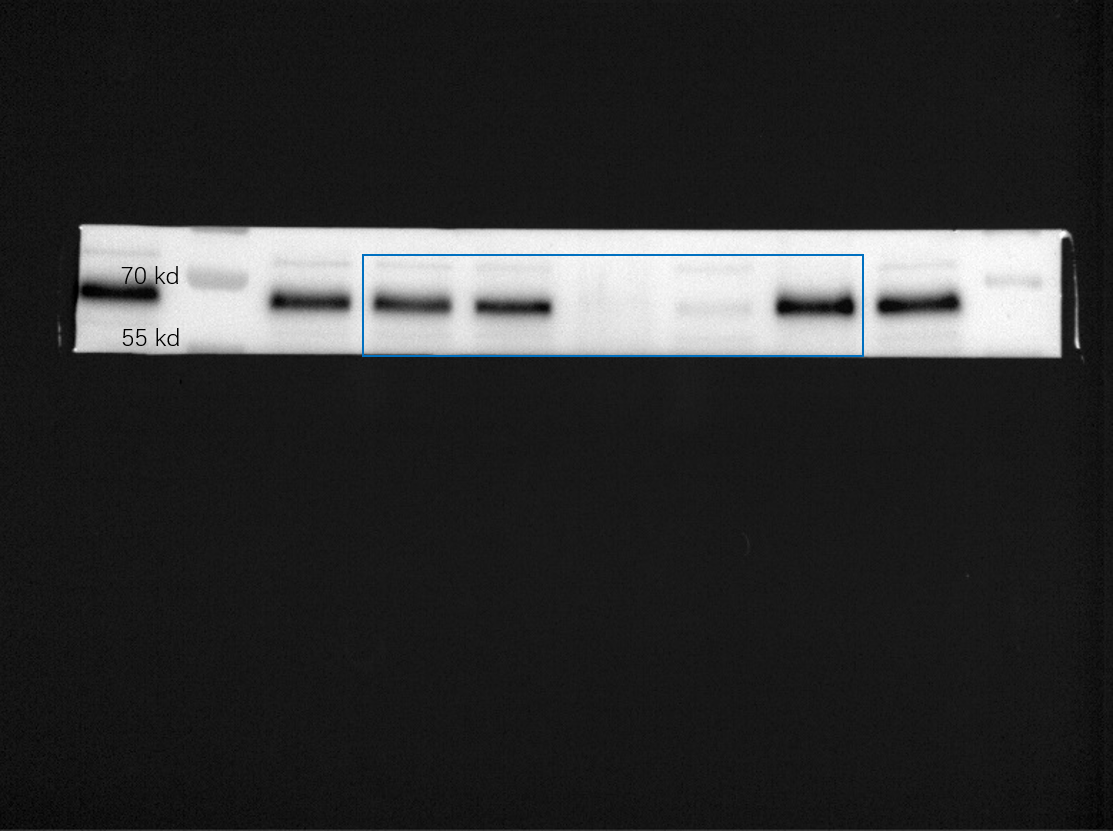

Supplement: Figure 1—figure supplement 1—source data 3. [file elife-89317-fig1-figsupp1-data3.zip › Labelled figures/syt labelled.png]

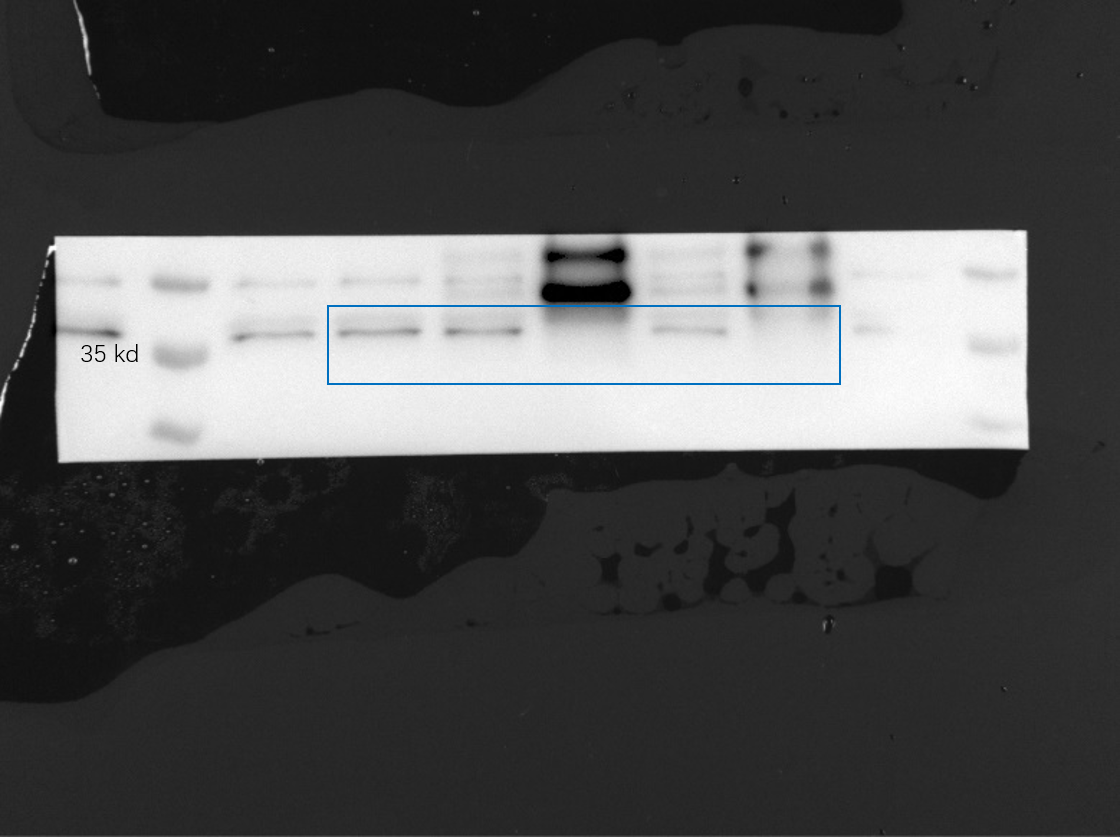

Supplement: Figure 1—figure supplement 1—source data 3. [file elife-89317-fig1-figsupp1-data3.zip › Labelled figures/VDAC labelled.png]

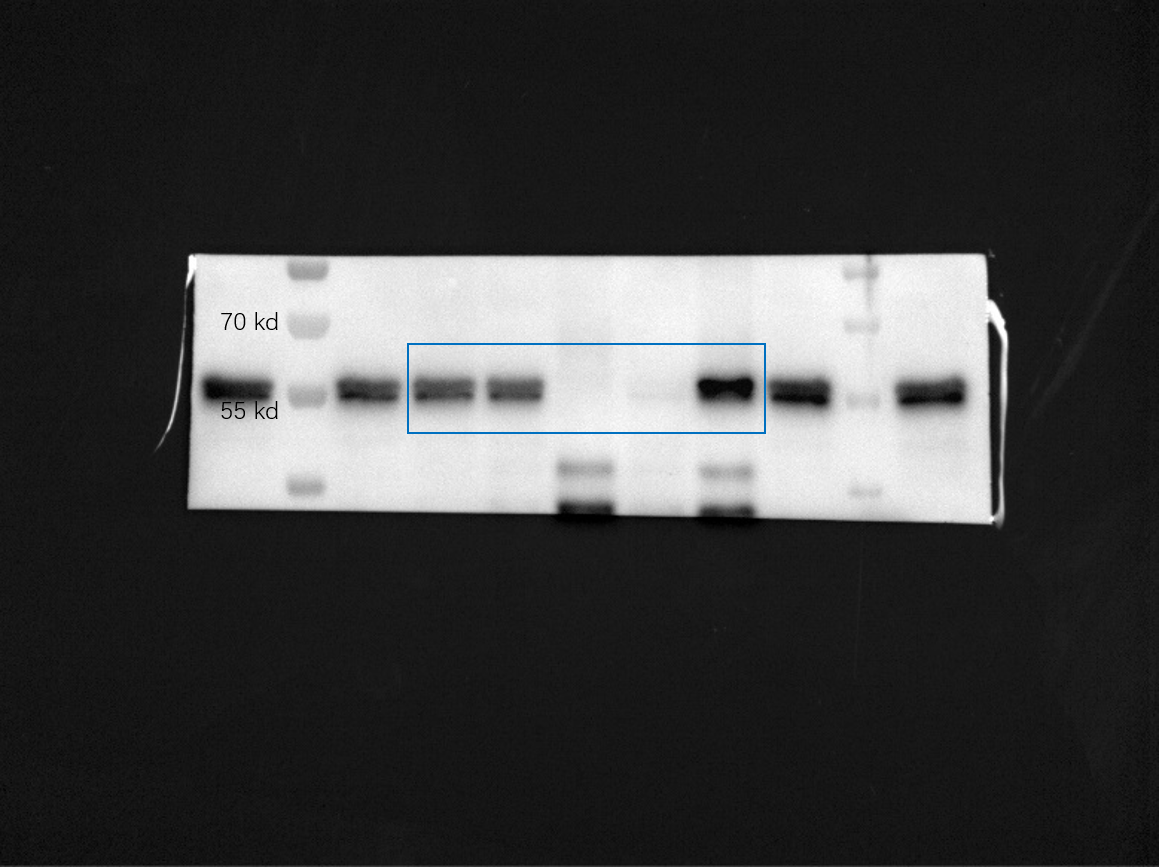

Supplement: Figure 1—figure supplement 1—source data 3. [file elife-89317-fig1-figsupp1-data3.zip › Labelled figures/VGAT labelled.png]

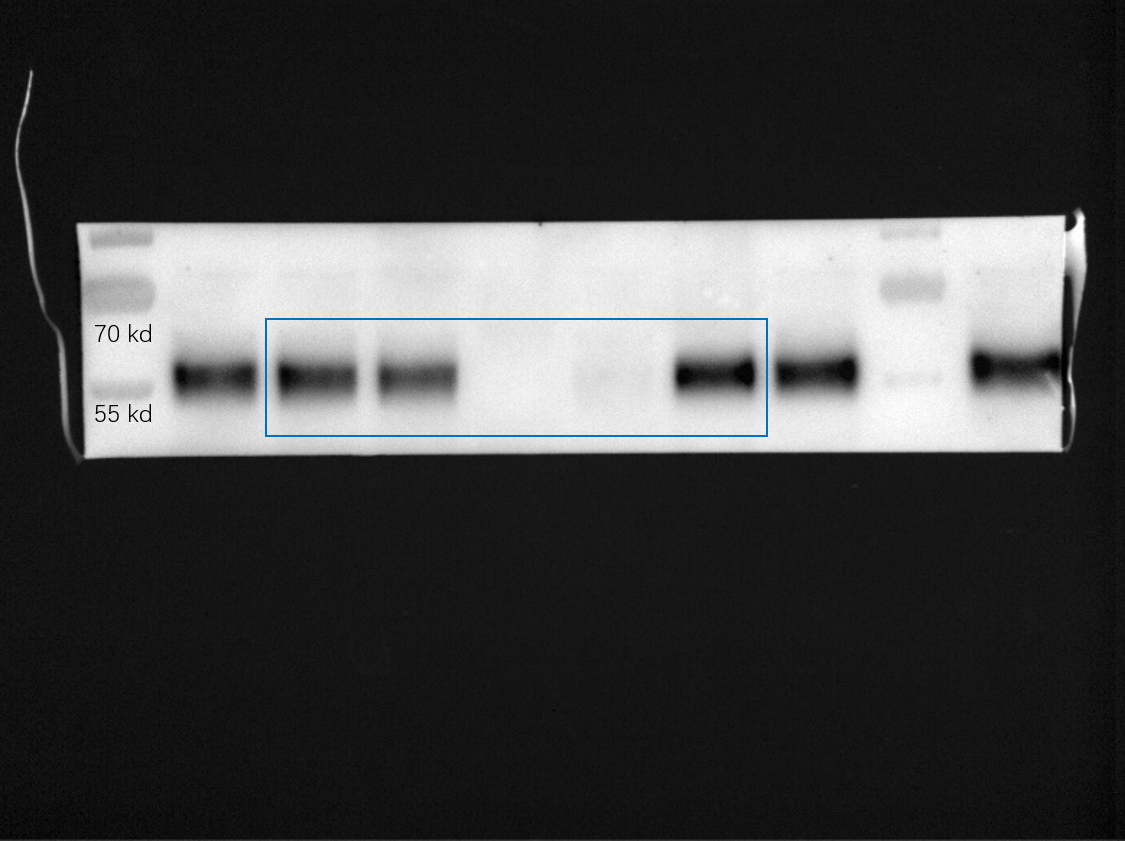

Supplement: Figure 1—figure supplement 1—source data 3. [file elife-89317-fig1-figsupp1-data3.zip › Labelled figures/VGlut1 labelled.png]

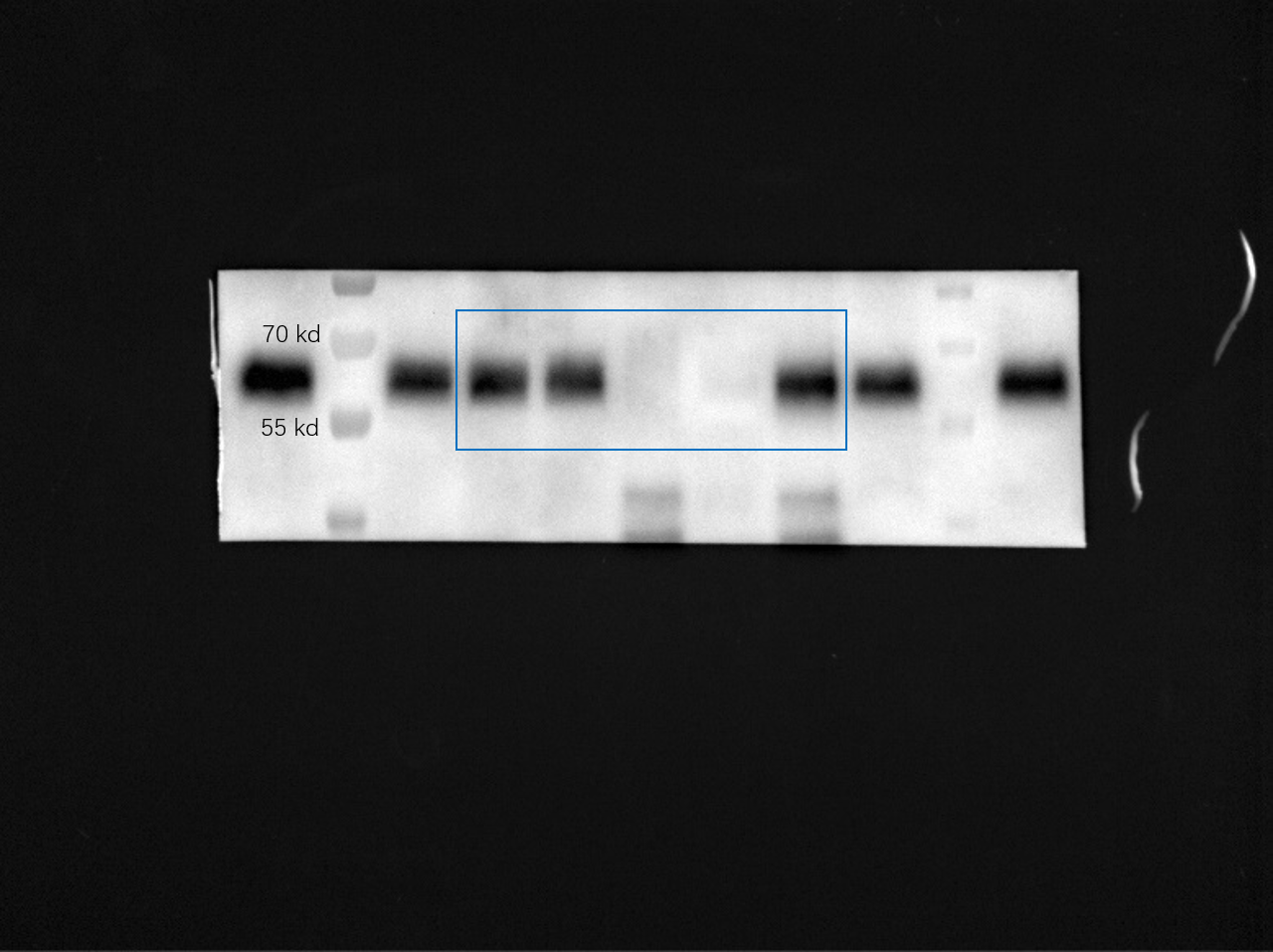

Supplement: Figure 1—figure supplement 1—source data 3. [file elife-89317-fig1-figsupp1-data3.zip › Labelled figures/VGlut2 labelled.png]

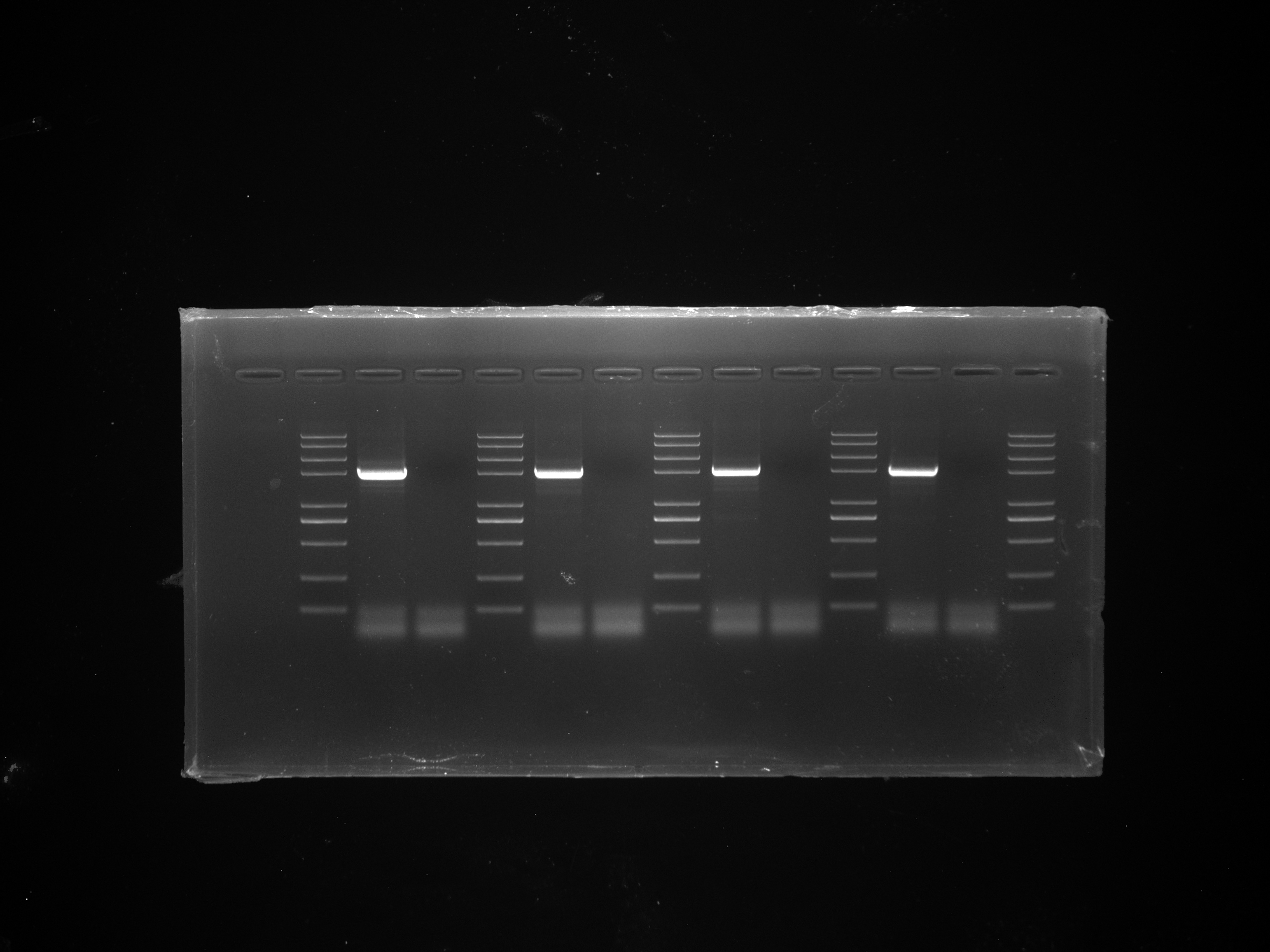

Supplement: Figure 2—figure supplement 1—source data 1. [file elife-89317-fig2-figsupp1-data1.zip › Figure 2-figure supplement 1-source data 1/Figure 2-Figure Supplement1A Slc6a8 original.Tif]

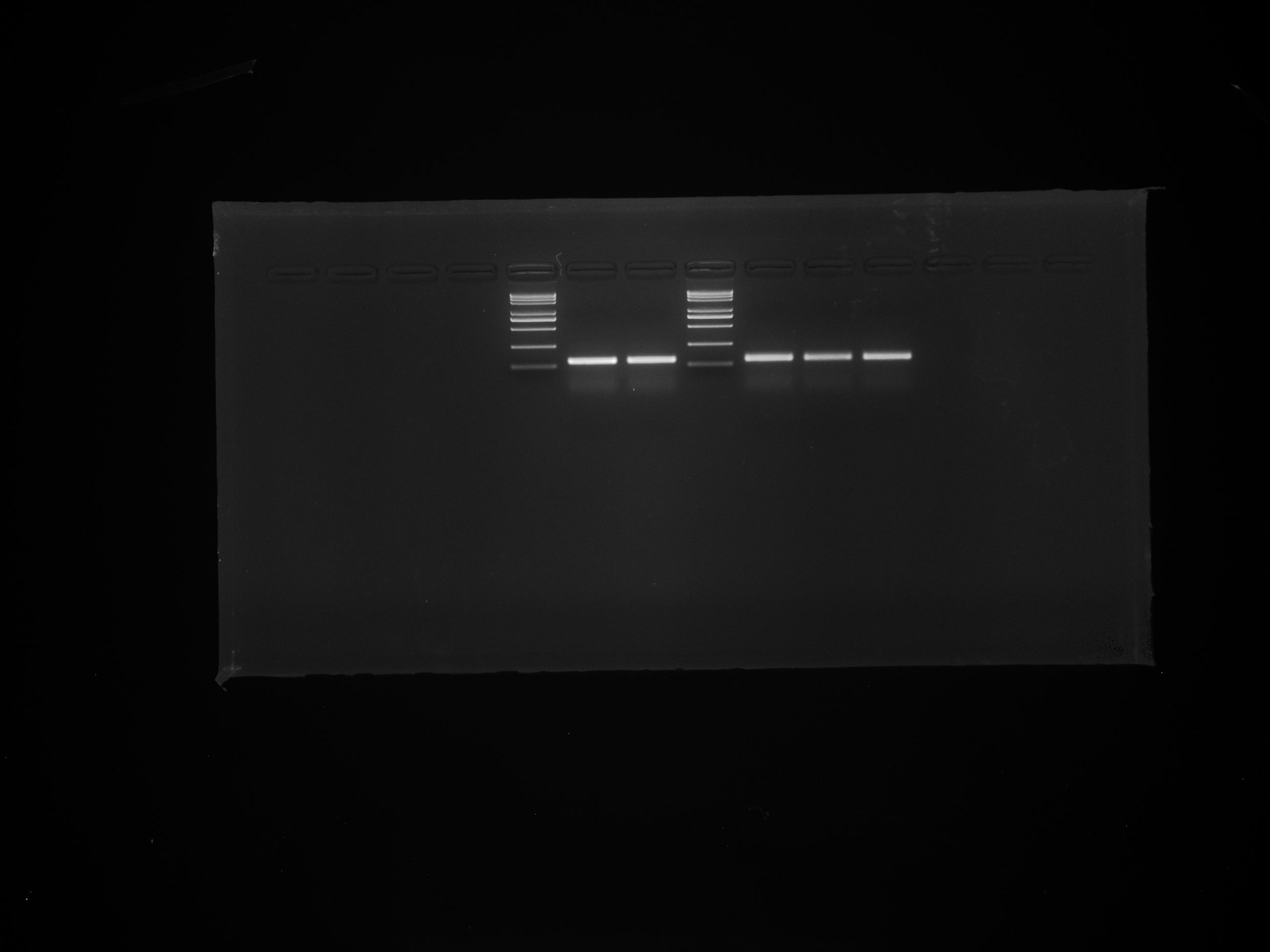

Supplement: Figure 2—figure supplement 1—source data 1. [file elife-89317-fig2-figsupp1-data1.zip › Figure 2-figure supplement 1-source data 1/Figure 2-Figure Supplement1B gapdh original.Tif]

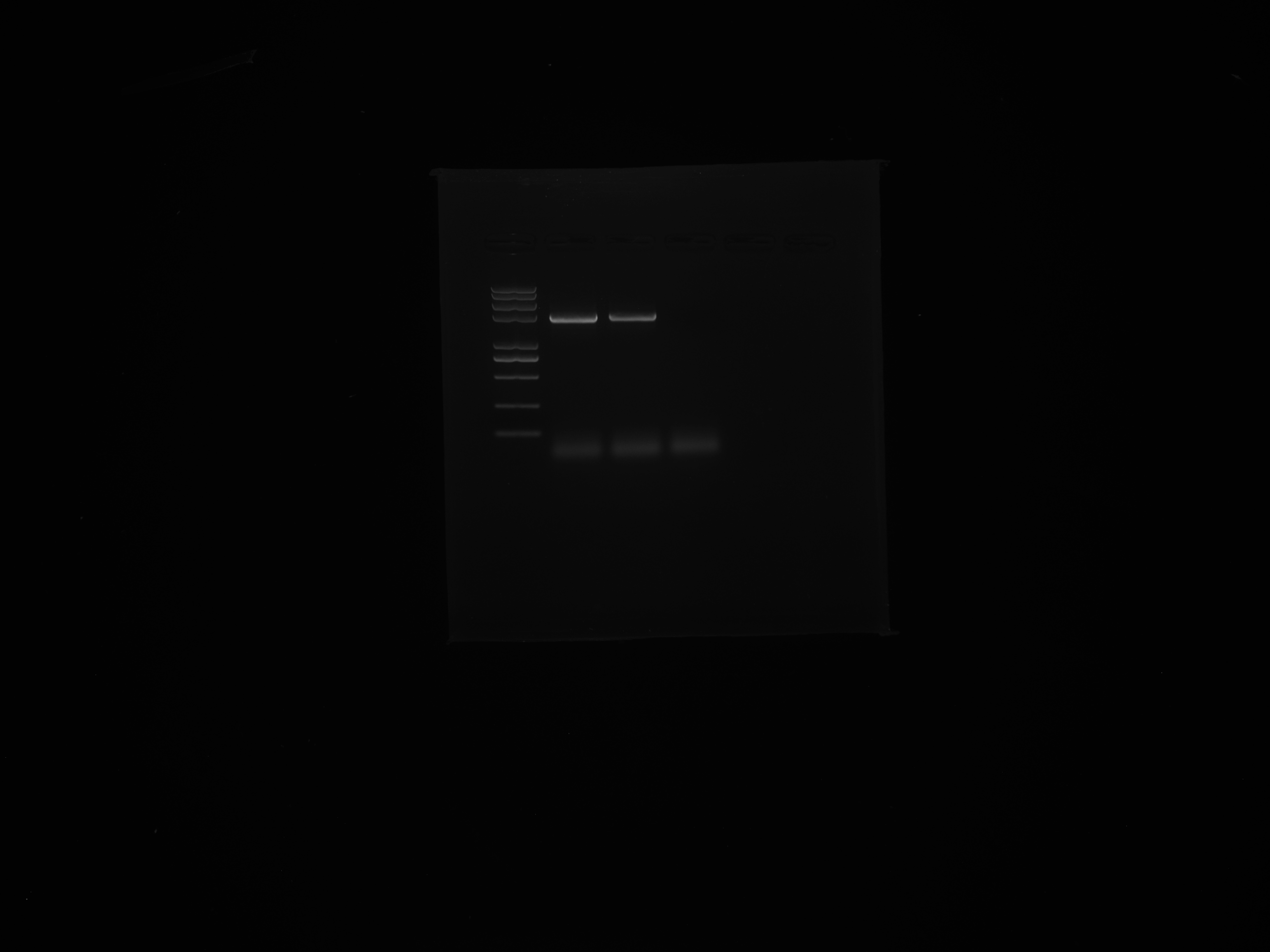

Supplement: Figure 2—figure supplement 1—source data 1. [file elife-89317-fig2-figsupp1-data1.zip › Figure 2-figure supplement 1-source data 1/Figure 2-Figure Supplement1B Slc6a8 original.Tif]

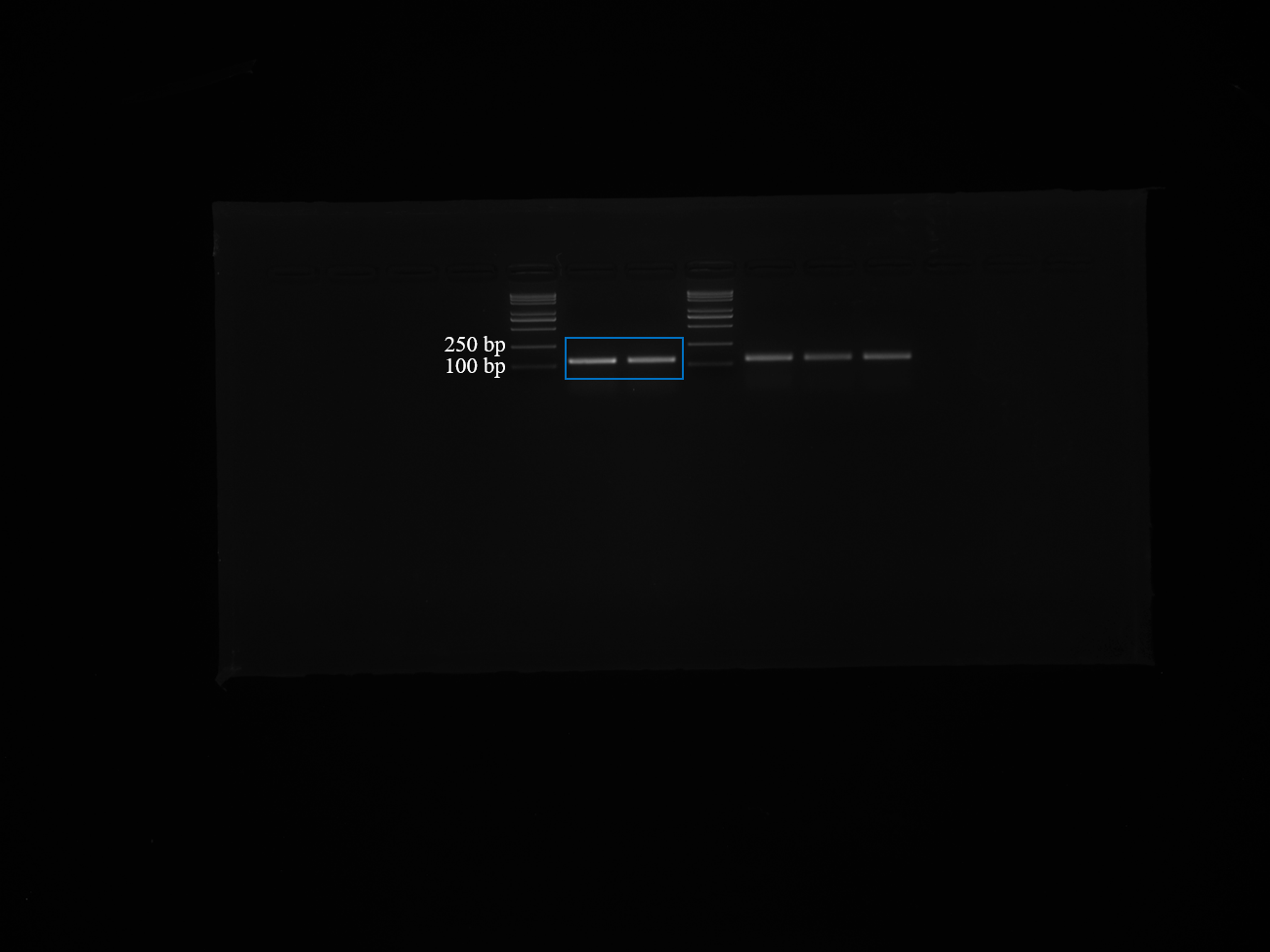

Supplement: Figure 2—figure supplement 1—source data 2. [file elife-89317-fig2-figsupp1-data2.zip › Figure 2-figure supplement 1-source data 2/Figure 2-Figure Supplement1A gapdh labelled.tif]

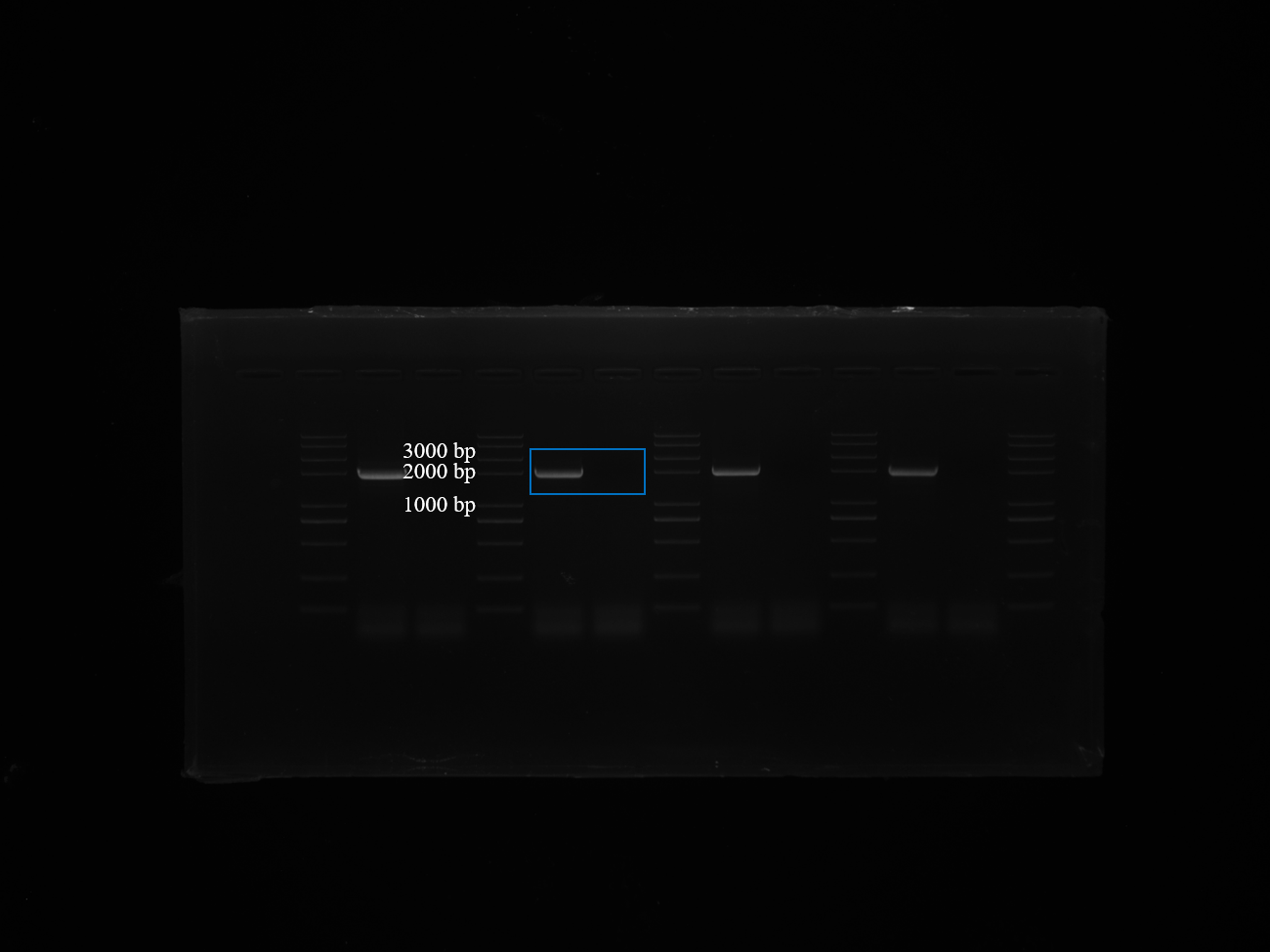

Supplement: Figure 2—figure supplement 1—source data 2. [file elife-89317-fig2-figsupp1-data2.zip › Figure 2-figure supplement 1-source data 2/Figure 2-Figure Supplement1A Slc6a8 labelled.tif]

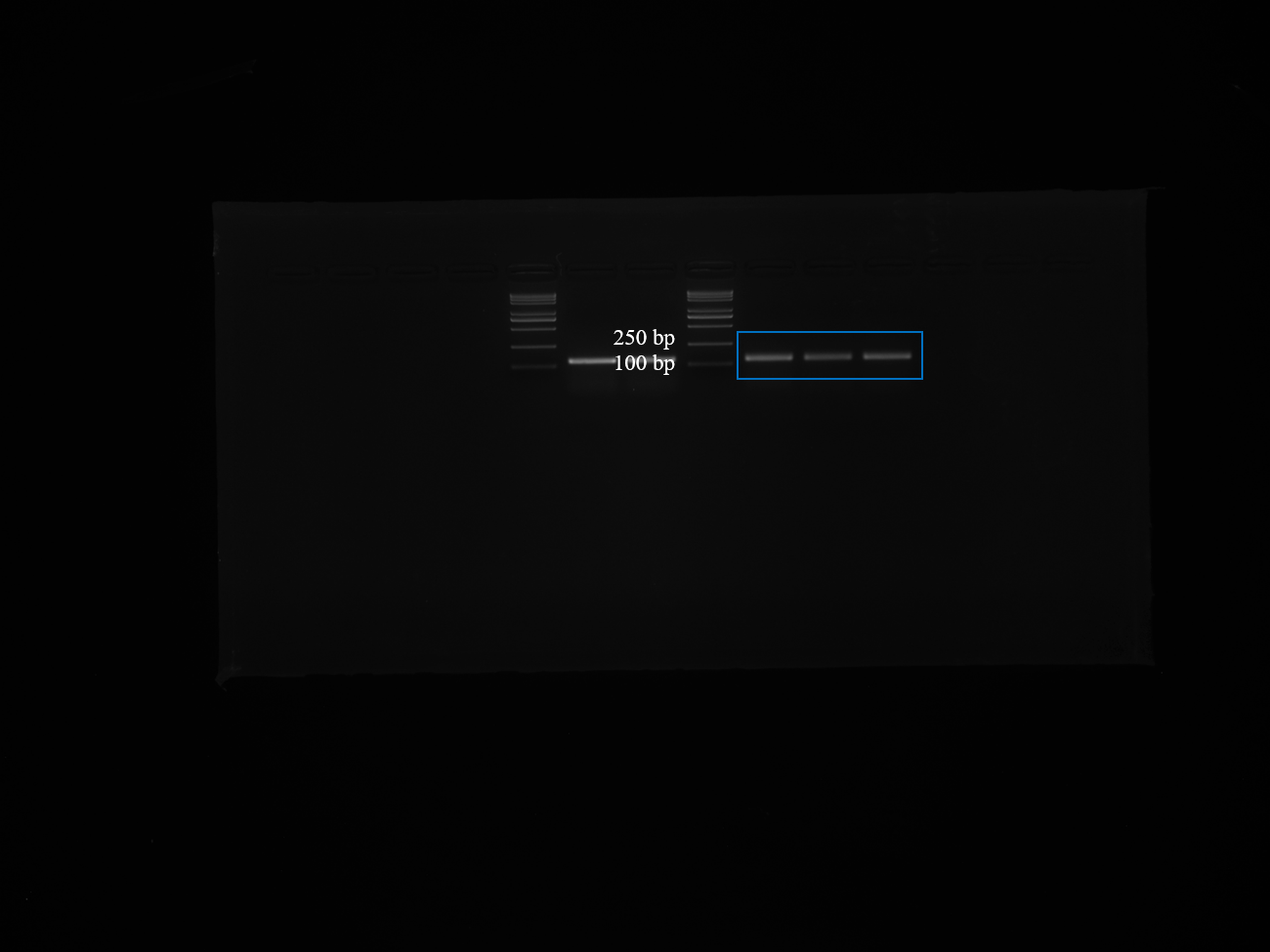

Supplement: Figure 2—figure supplement 1—source data 2. [file elife-89317-fig2-figsupp1-data2.zip › Figure 2-figure supplement 1-source data 2/Figure 2-Figure Supplement1B gapdh labelled.tif]

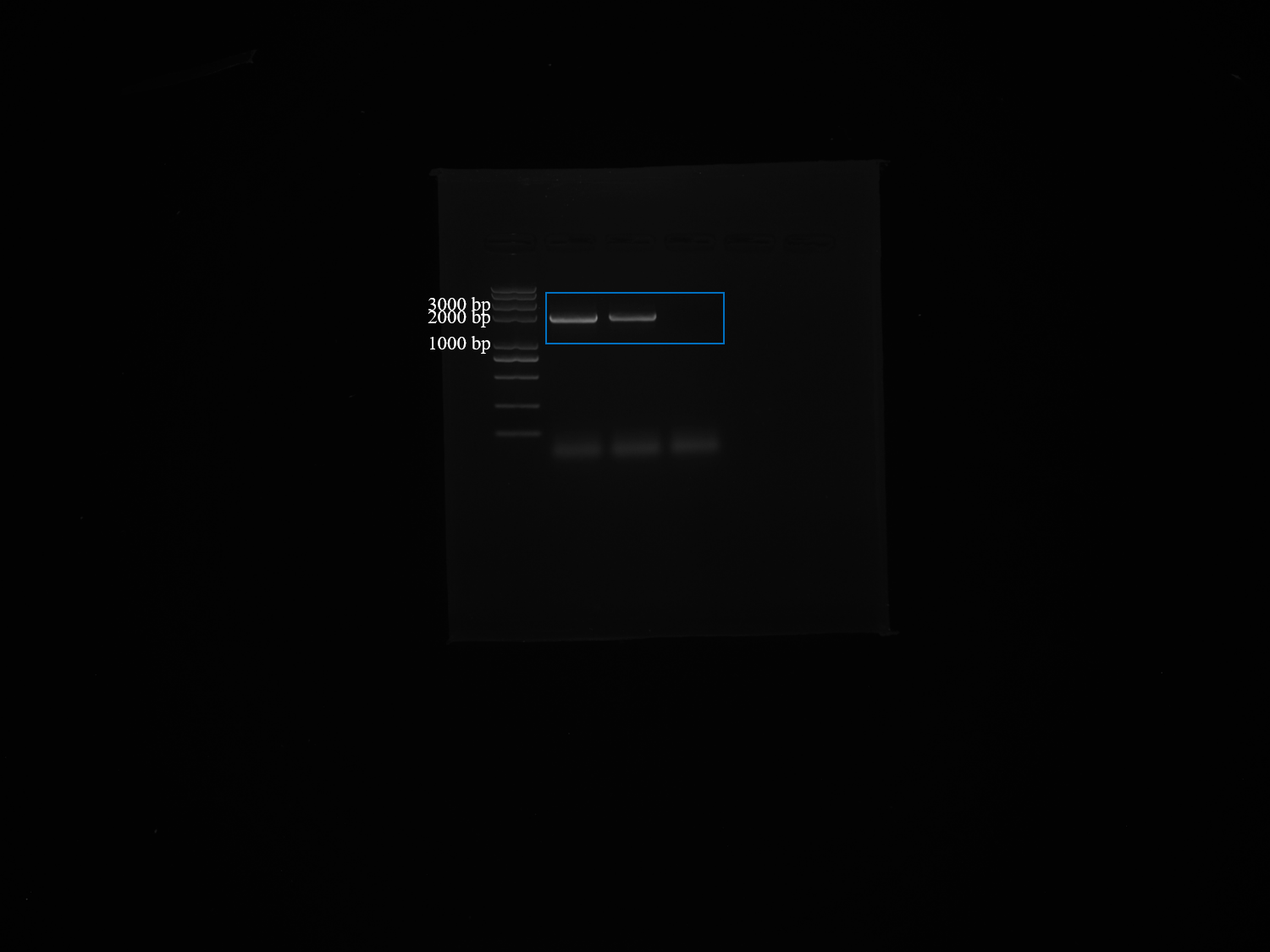

Supplement: Figure 2—figure supplement 1—source data 2. [file elife-89317-fig2-figsupp1-data2.zip › Figure 2-figure supplement 1-source data 2/Figure 2-Figure Supplement1B Slc6a8 labelled.tif]

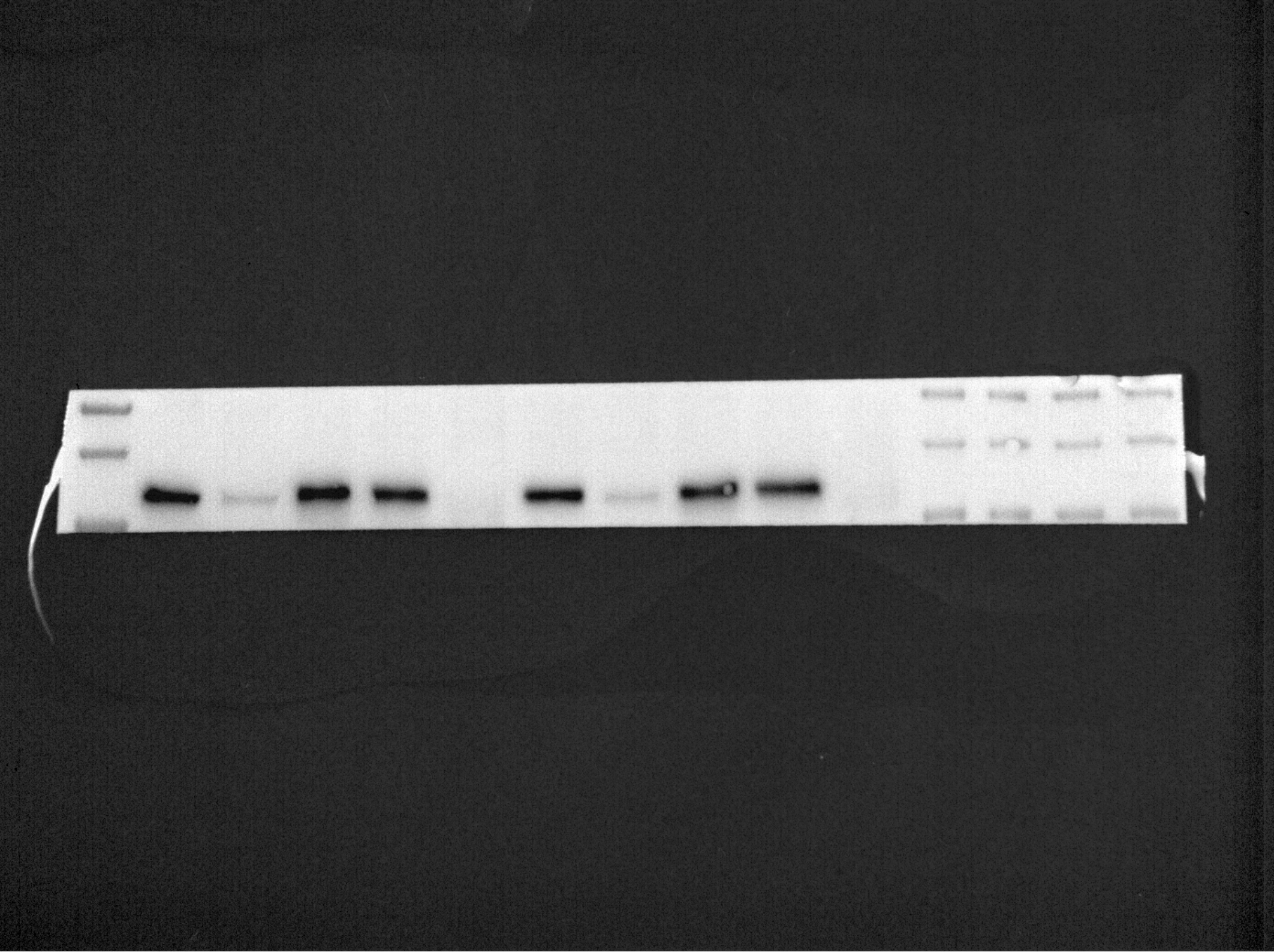

Supplement: Figure 2—figure supplement 4—source data 1. [file elife-89317-fig2-figsupp4-data1.zip › Figure 2-figure supplement 4-source data 1/Figure 2-Figure Supplement 4A-H-ATPase original.jpg]

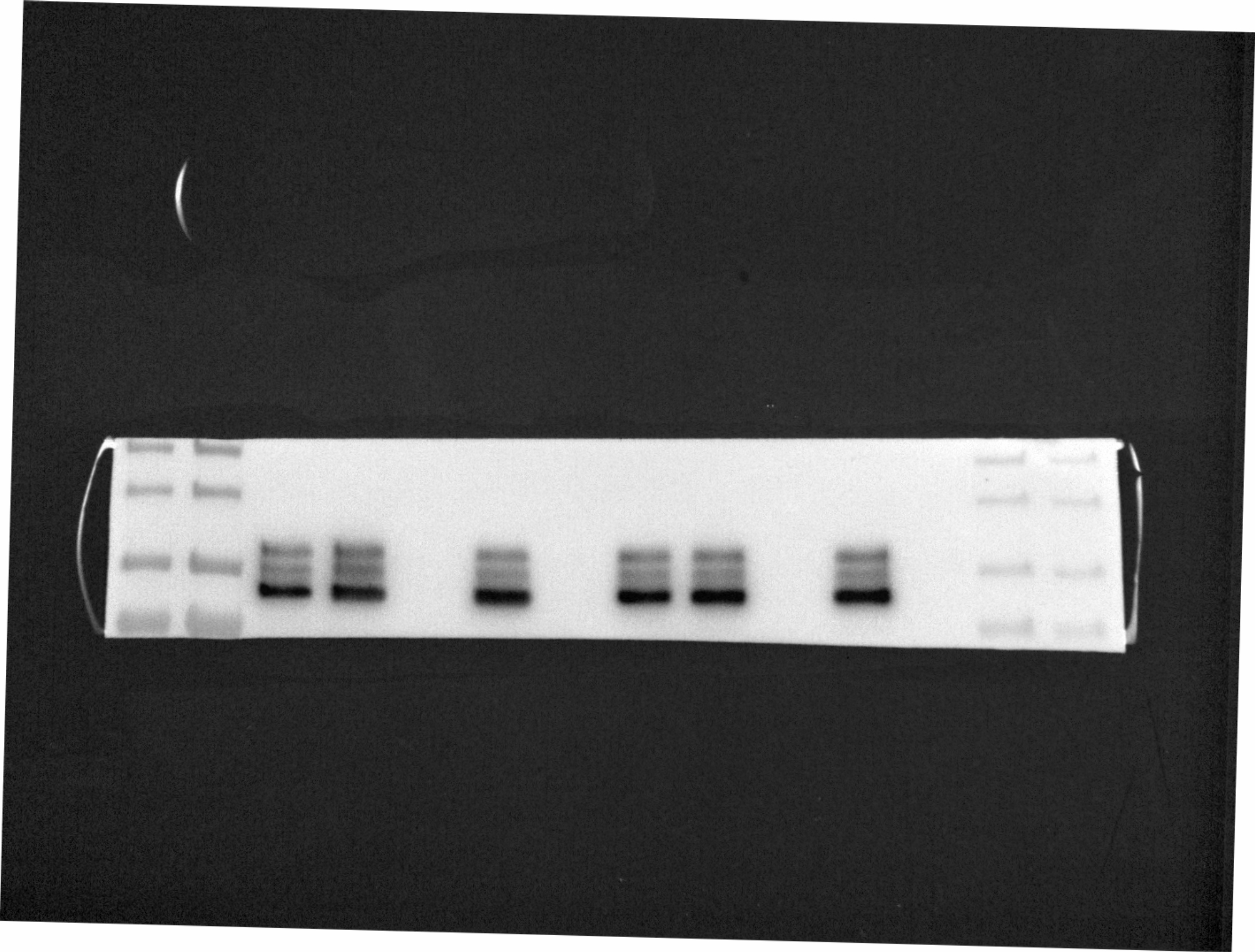

Supplement: Figure 2—figure supplement 4—source data 1. [file elife-89317-fig2-figsupp4-data1.zip › Figure 2-figure supplement 4-source data 1/Figure 2-Figure Supplement 4A-PSD95 original.jpg]

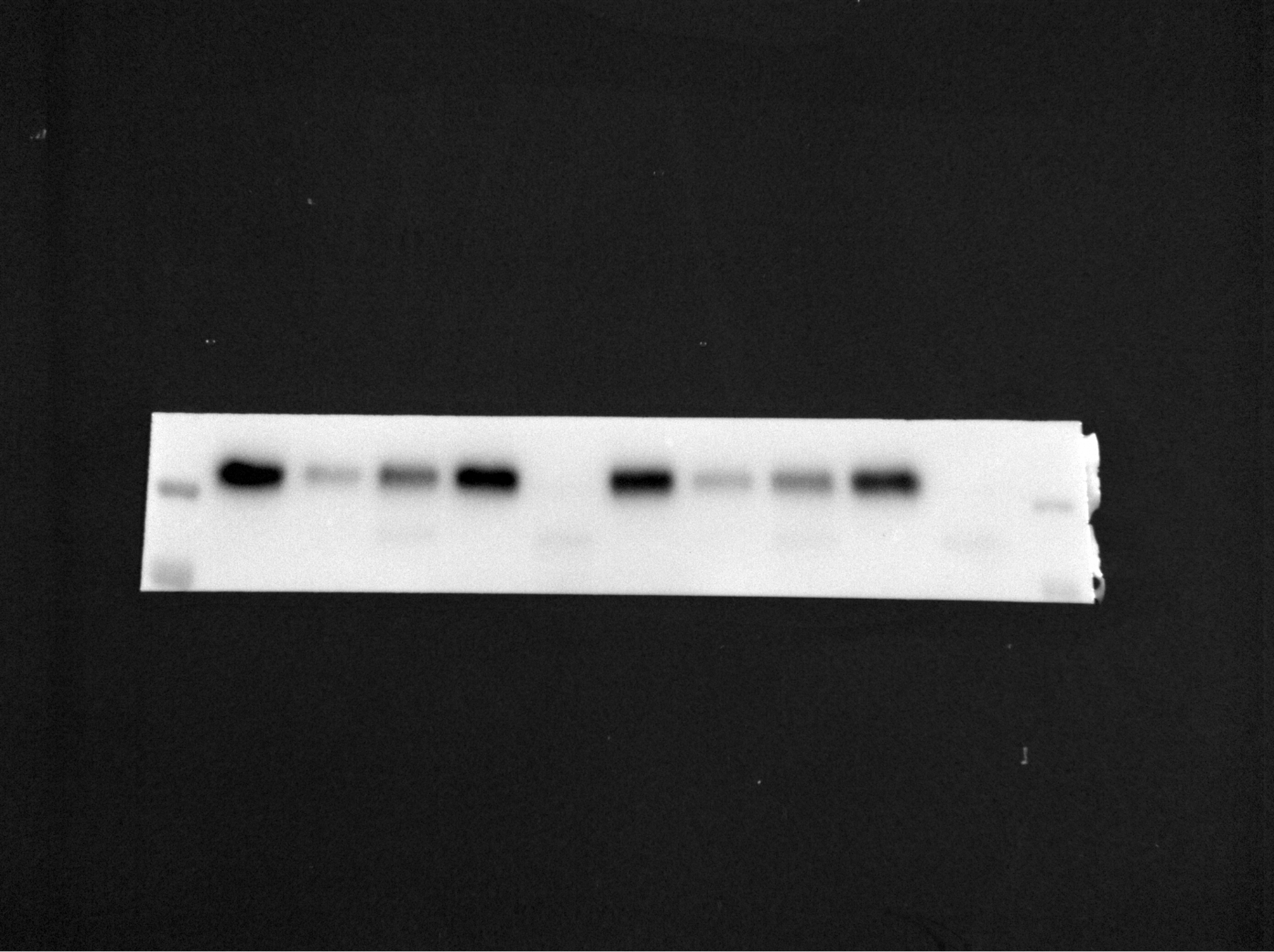

Supplement: Figure 2—figure supplement 4—source data 1. [file elife-89317-fig2-figsupp4-data1.zip › Figure 2-figure supplement 4-source data 1/Figure 2-Figure Supplement 4A-Syp original.jpg]

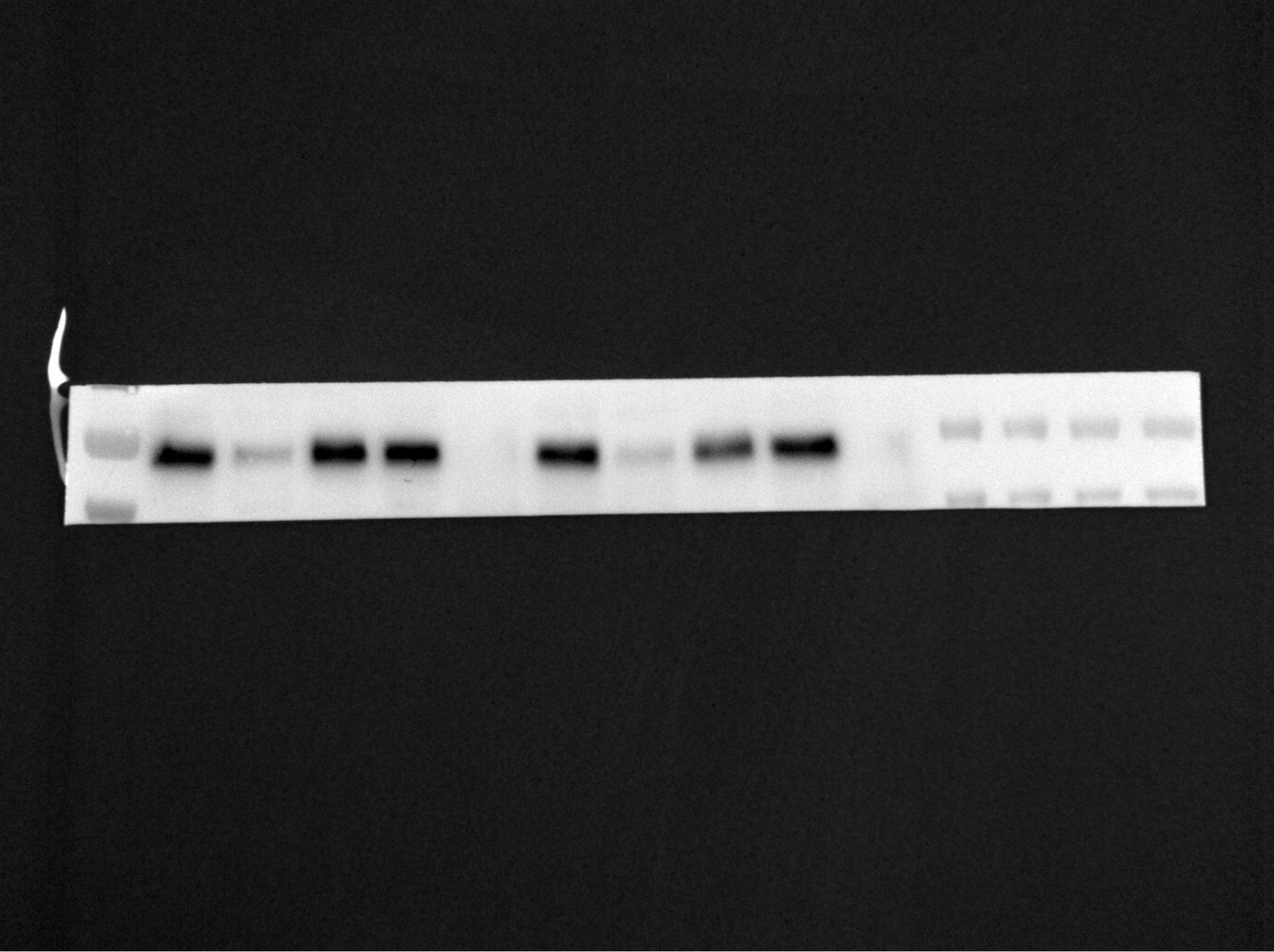

Supplement: Figure 2—figure supplement 4—source data 1. [file elife-89317-fig2-figsupp4-data1.zip › Figure 2-figure supplement 4-source data 1/Figure 2-Figure Supplement 4A-syt original.jpg]

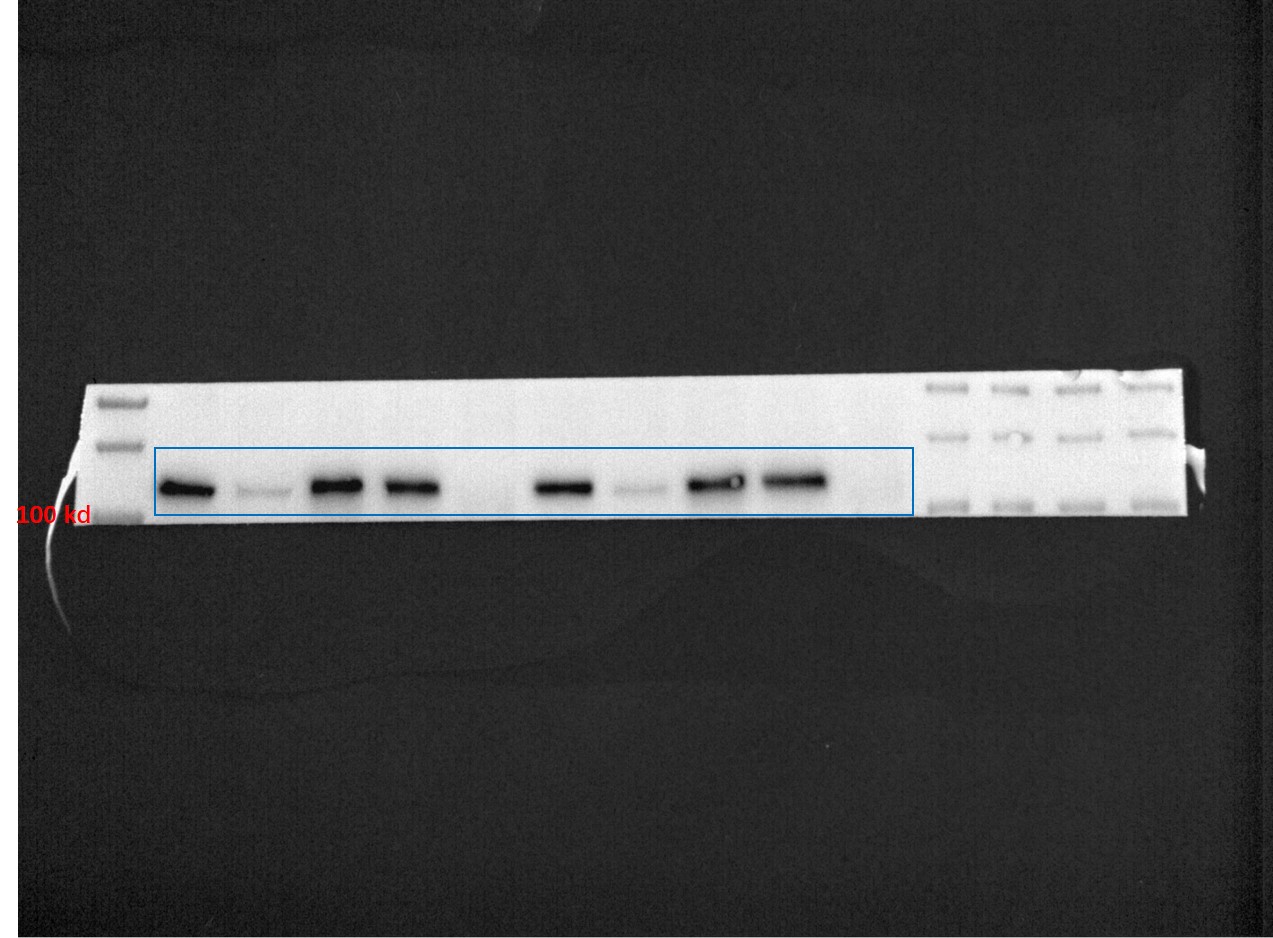

Supplement: Figure 2—figure supplement 4—source data 2. [file elife-89317-fig2-figsupp4-data2.zip › Figure 2-figure supplement 4-source data 2/Figure 2-Figure Supplement 4A-H-ATPase labelled.jpg]

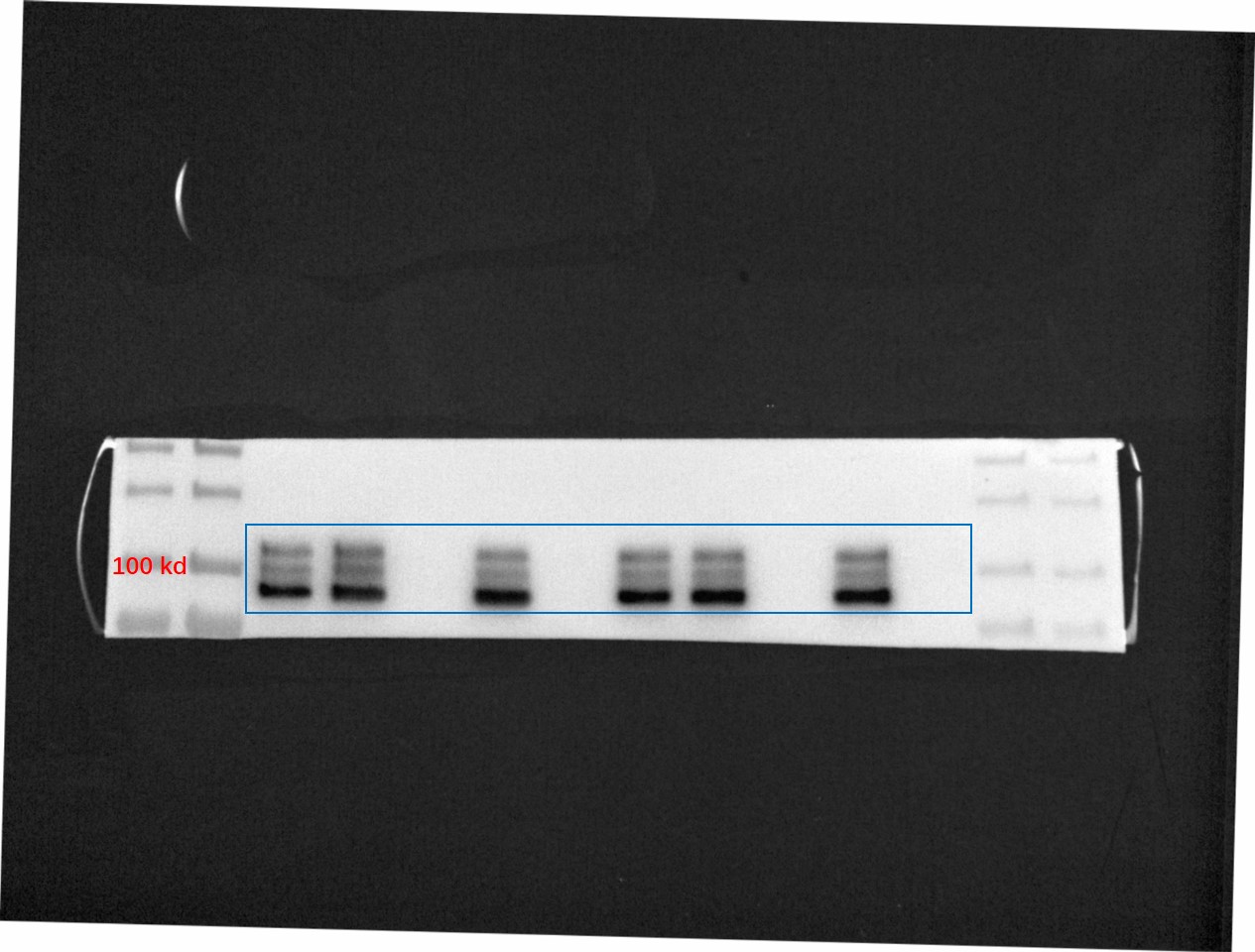

Supplement: Figure 2—figure supplement 4—source data 2. [file elife-89317-fig2-figsupp4-data2.zip › Figure 2-figure supplement 4-source data 2/Figure 2-Figure Supplement 4A-PSD95 labelled.jpg]

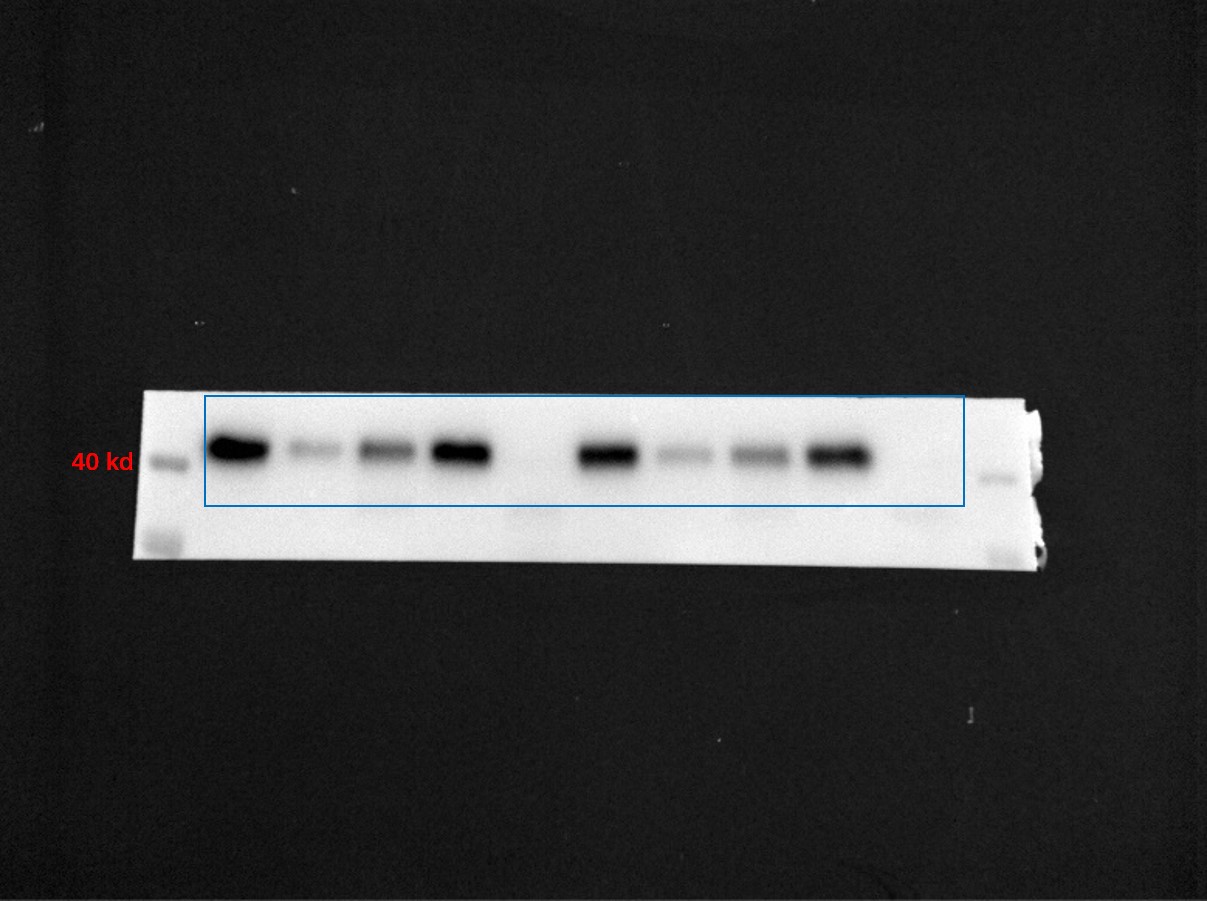

Supplement: Figure 2—figure supplement 4—source data 2. [file elife-89317-fig2-figsupp4-data2.zip › Figure 2-figure supplement 4-source data 2/Figure 2-Figure Supplement 4A-Syp labelled.jpg]

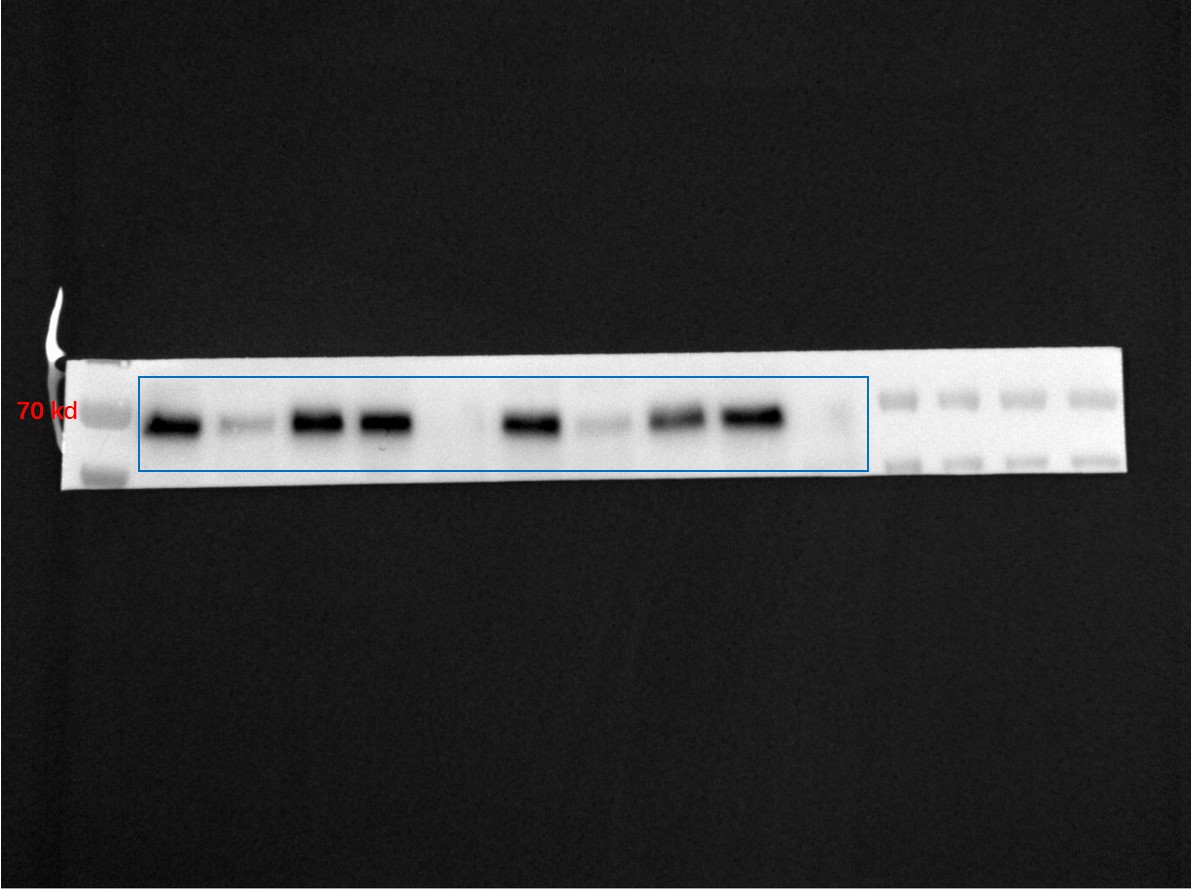

Supplement: Figure 2—figure supplement 4—source data 2. [file elife-89317-fig2-figsupp4-data2.zip › Figure 2-figure supplement 4-source data 2/Figure 2-Figure Supplement 4A-syt labelled.jpg]

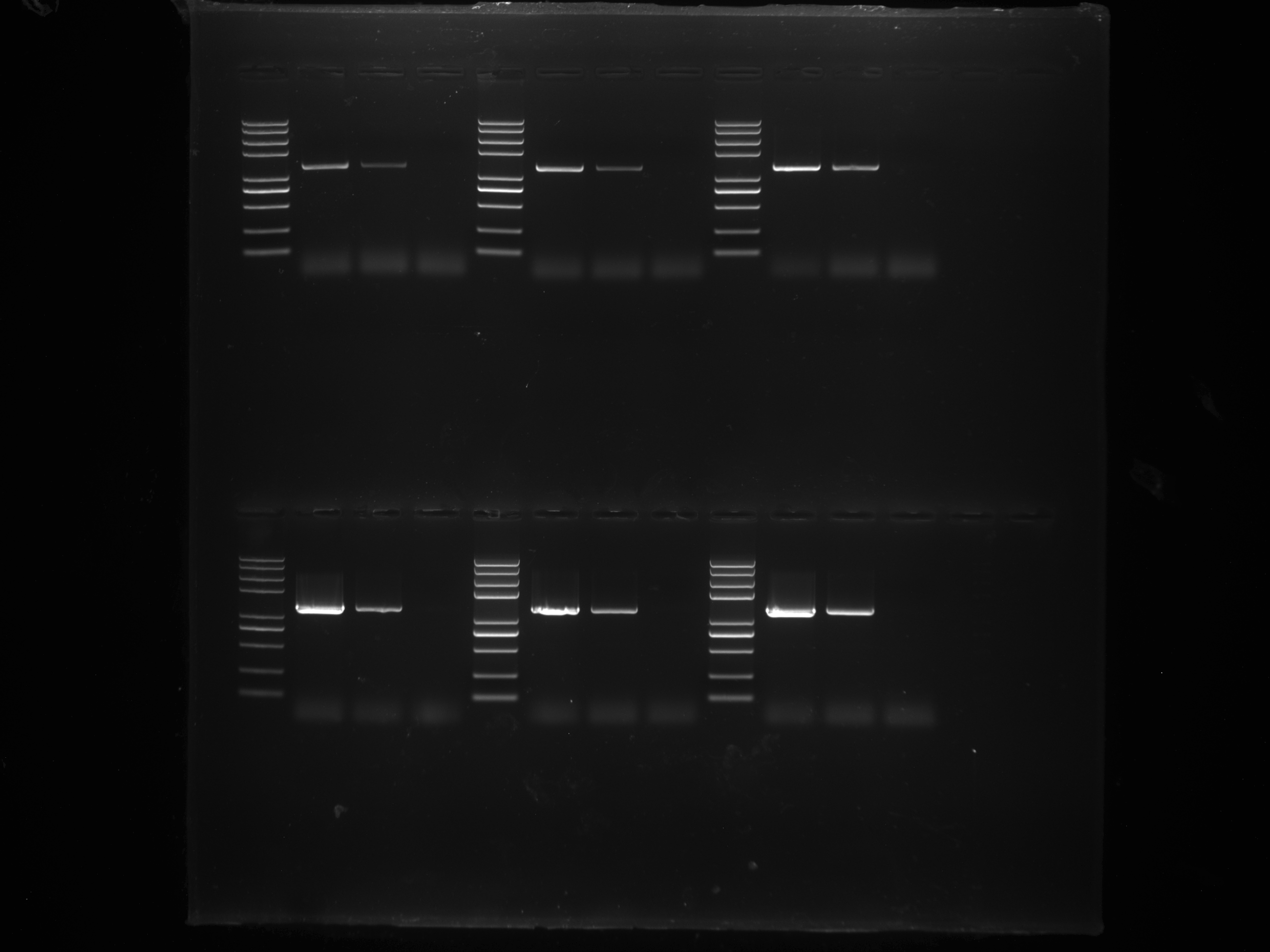

Supplement: Figure 3—figure supplement 1—source data 1. [file elife-89317-fig3-figsupp1-data1.zip › Figure 3-figure supplement 1-source data 1/Agat original.Tif]

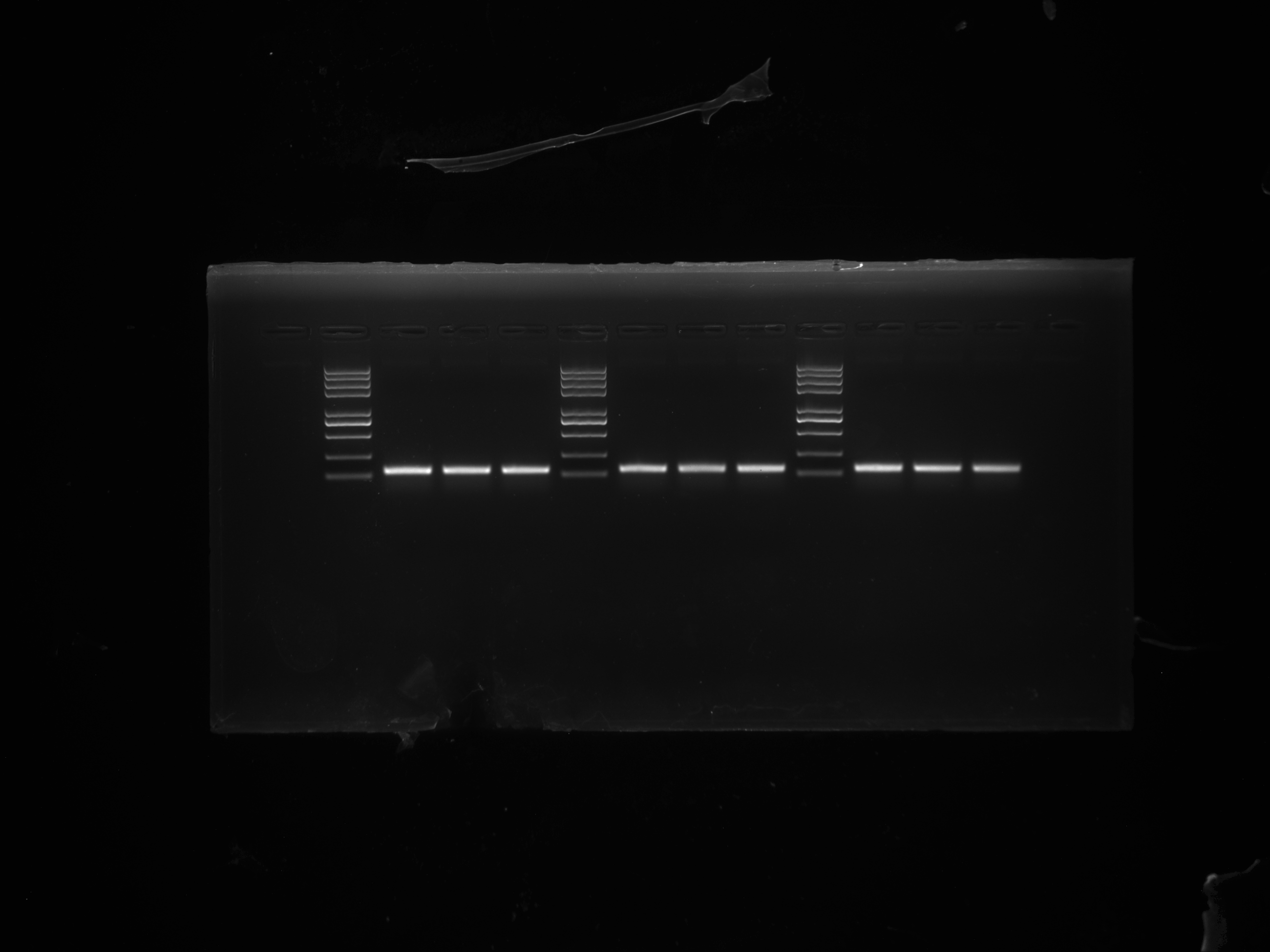

Supplement: Figure 3—figure supplement 1—source data 1. [file elife-89317-fig3-figsupp1-data1.zip › Figure 3-figure supplement 1-source data 1/Gapdh original.Tif]

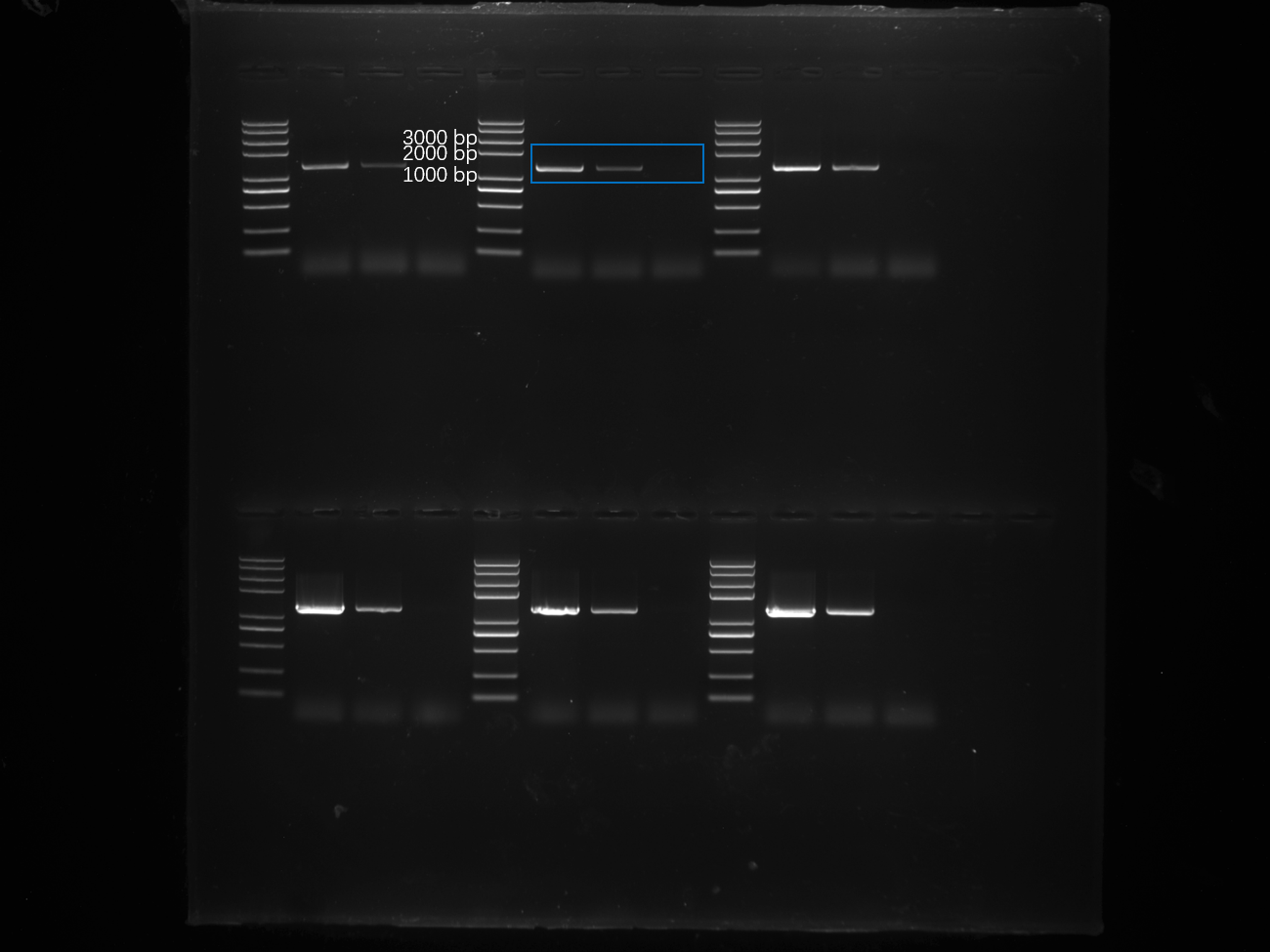

Supplement: Figure 3—figure supplement 1—source data 2. [file elife-89317-fig3-figsupp1-data2.zip › Figure 3-figure supplement 1-source data 2/Agat labelled.tif]

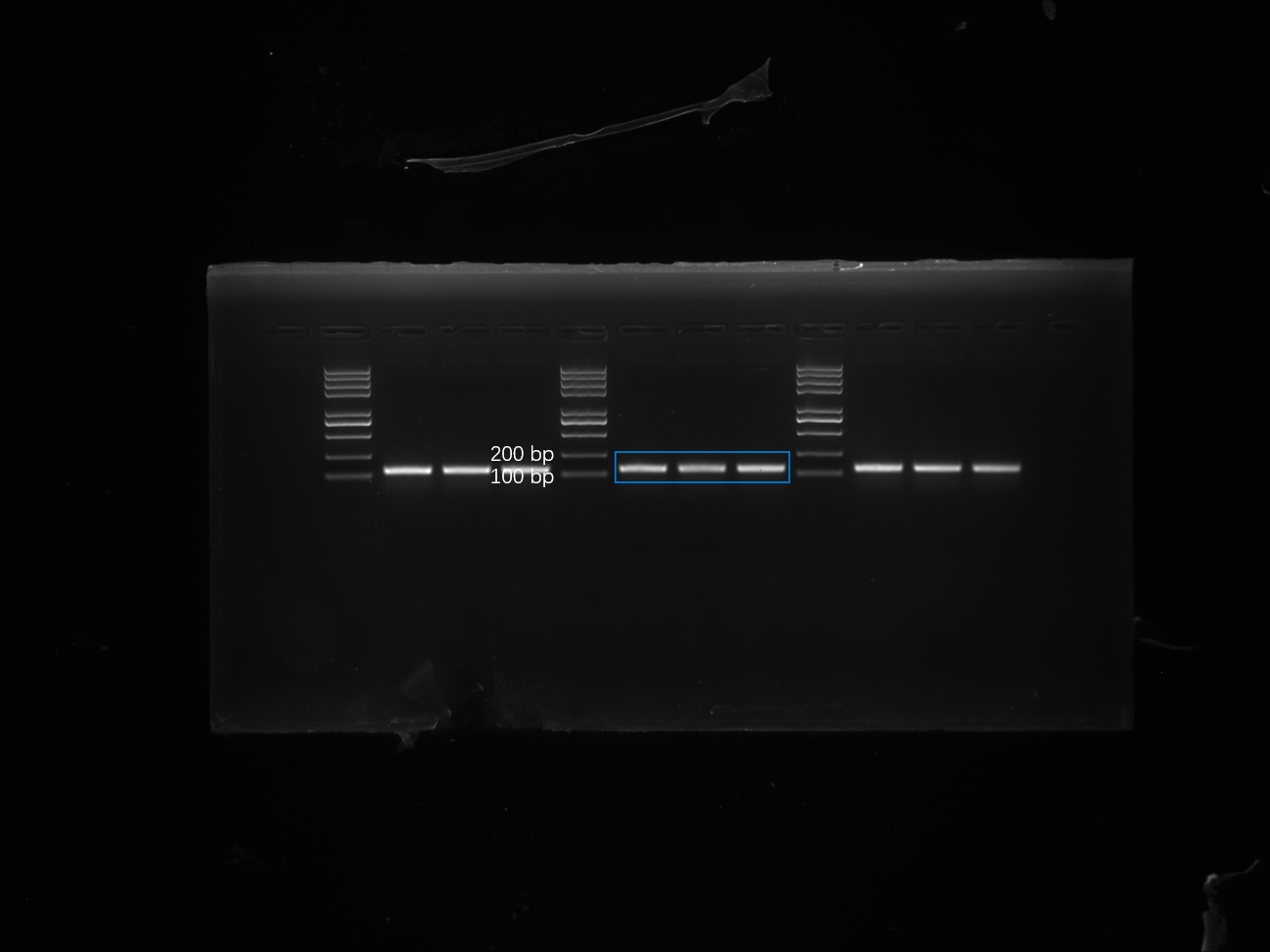

Supplement: Figure 3—figure supplement 1—source data 2. [file elife-89317-fig3-figsupp1-data2.zip › Figure 3-figure supplement 1-source data 2/Gapdh labelled.tif]

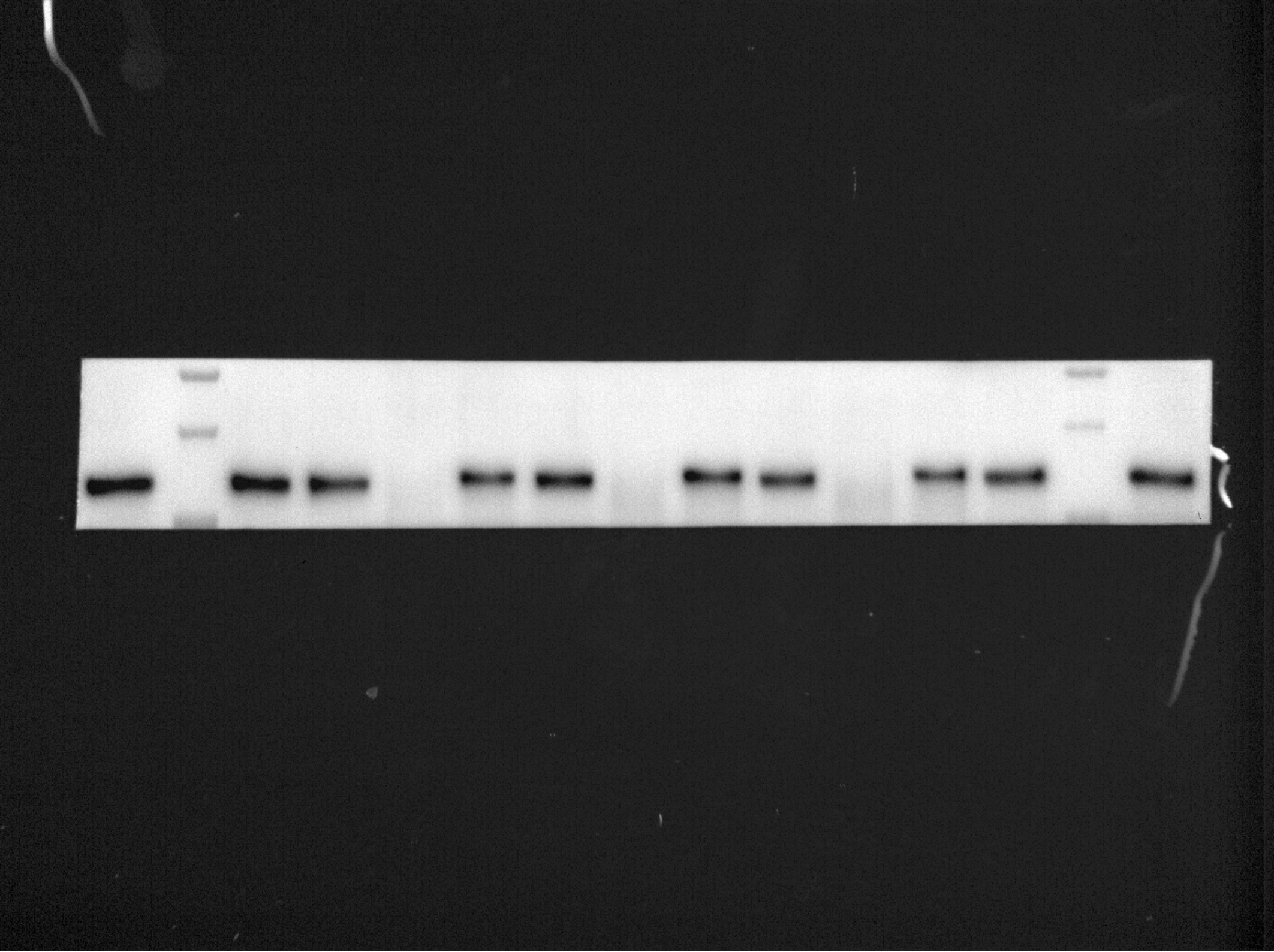

Supplement: Figure 3—figure supplement 3—source data 1. [file elife-89317-fig3-figsupp3-data1.zip › Figure 3-figure supplement 3-source data 1/H-ATPase original.jpg]

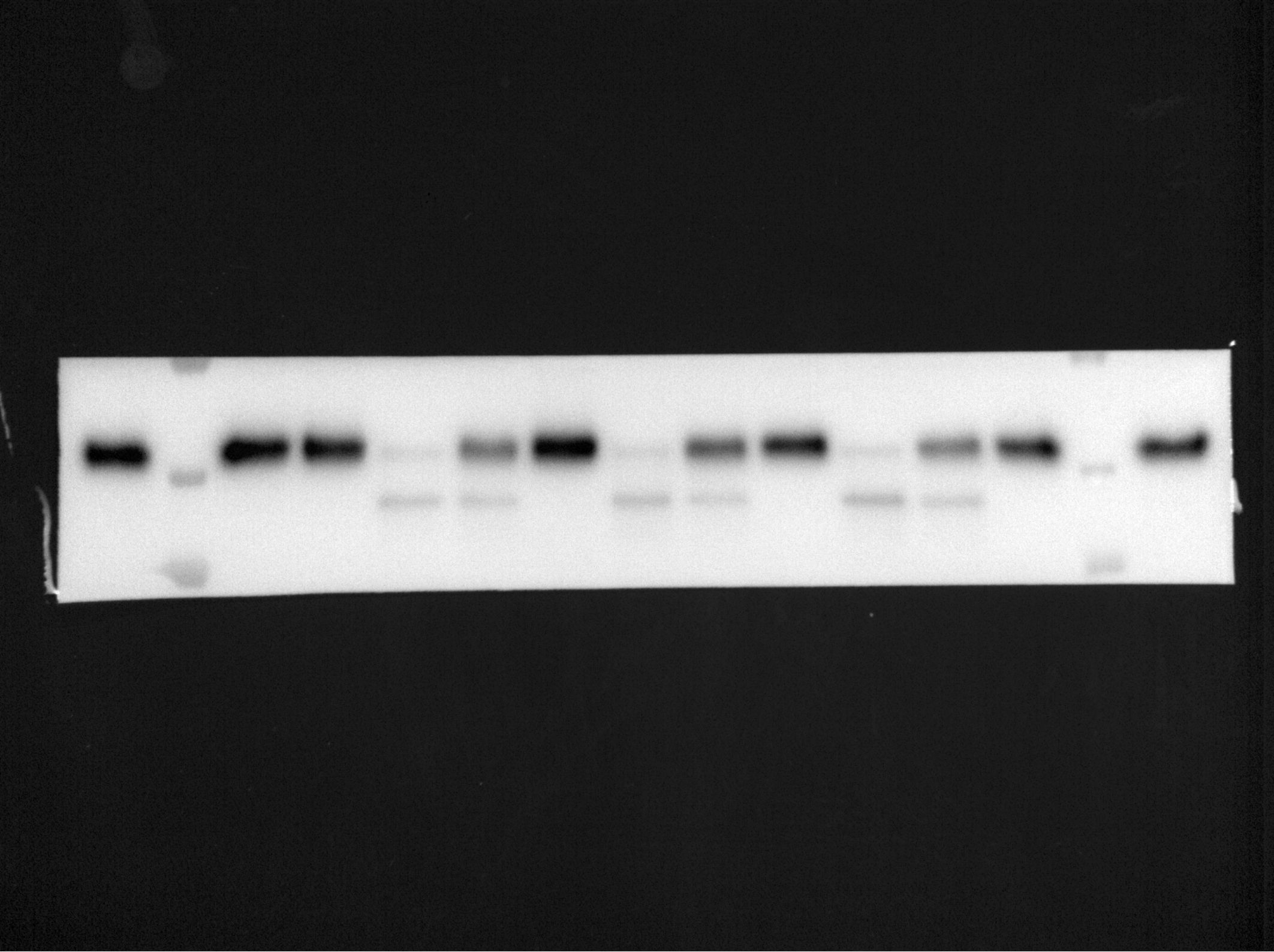

Supplement: Figure 3—figure supplement 3—source data 1. [file elife-89317-fig3-figsupp3-data1.zip › Figure 3-figure supplement 3-source data 1/Syp original.jpg]

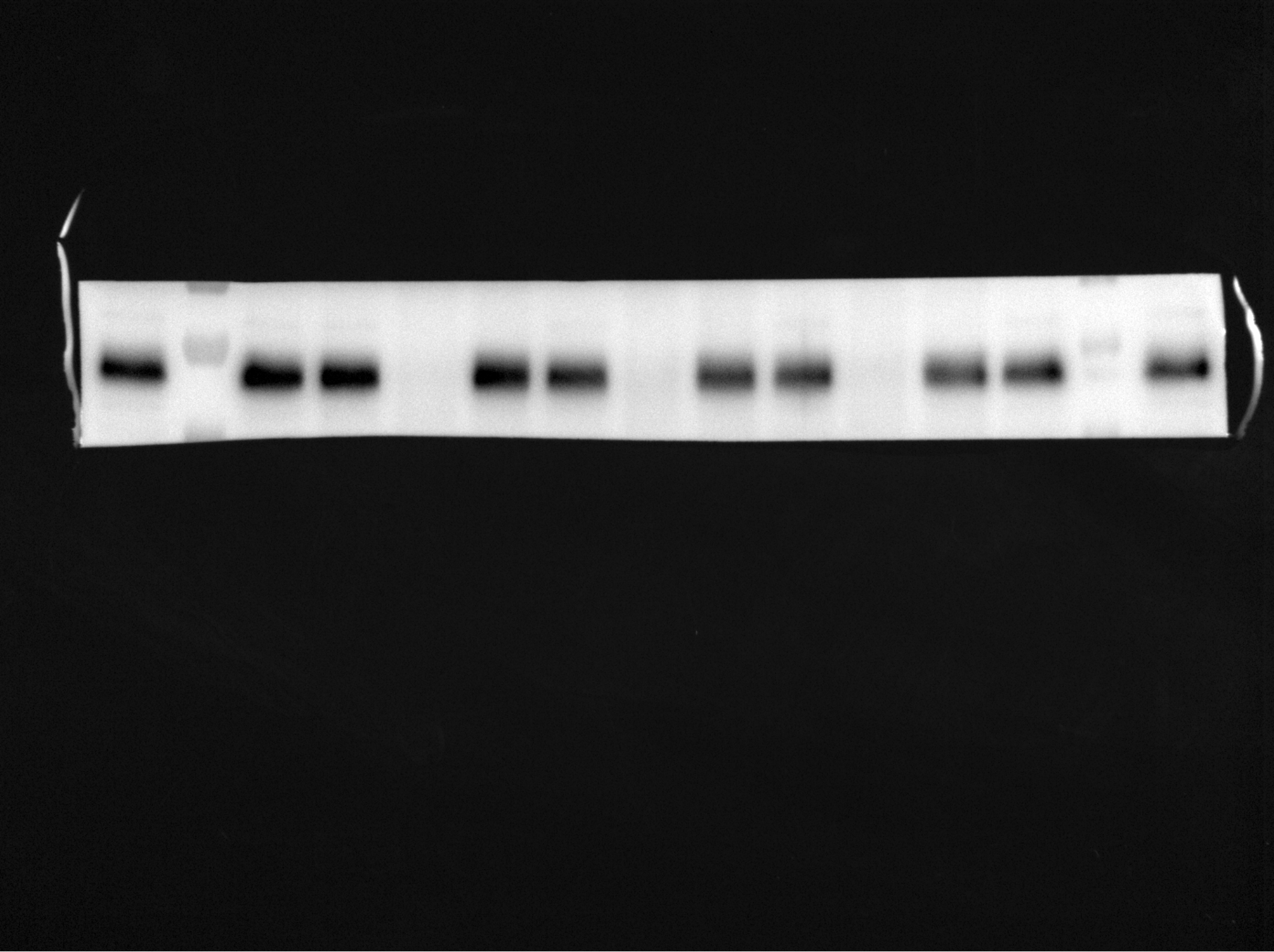

Supplement: Figure 3—figure supplement 3—source data 1. [file elife-89317-fig3-figsupp3-data1.zip › Figure 3-figure supplement 3-source data 1/syt original.jpg]

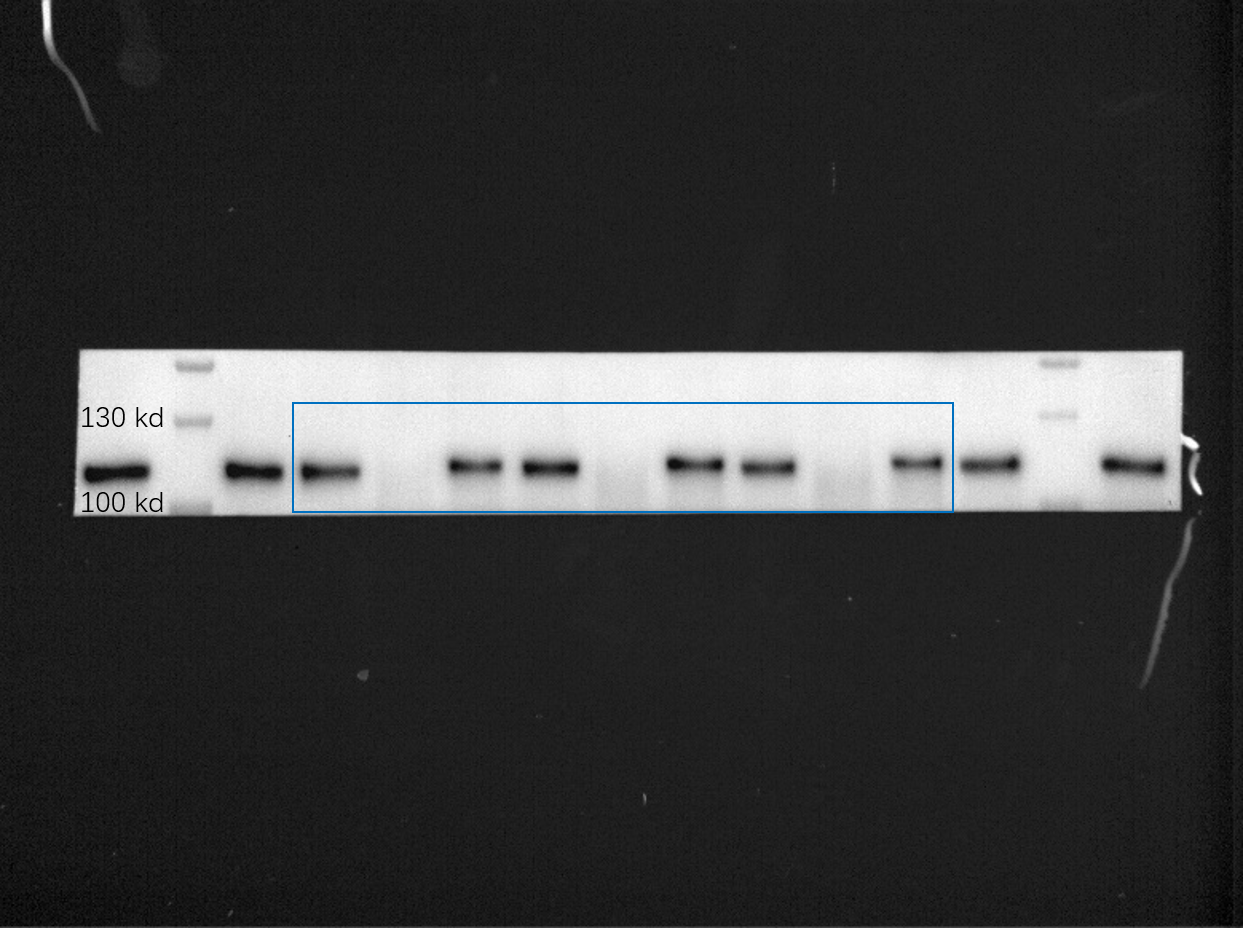

Supplement: Figure 3—figure supplement 3—source data 2. [file elife-89317-fig3-figsupp3-data2.zip › H-ATPase labelled.tif]

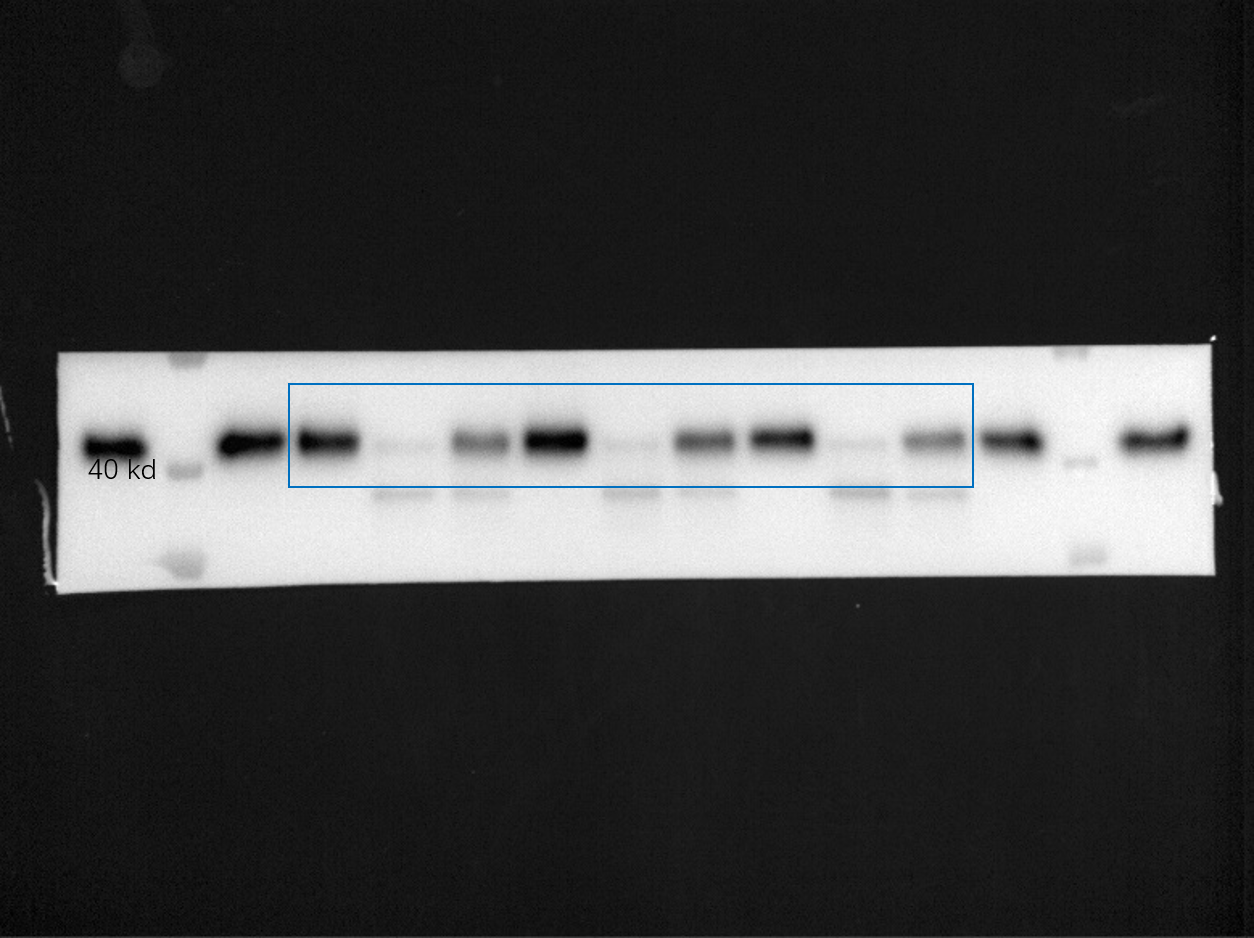

Supplement: Figure 3—figure supplement 3—source data 2. [file elife-89317-fig3-figsupp3-data2.zip › syp labelled.tif]

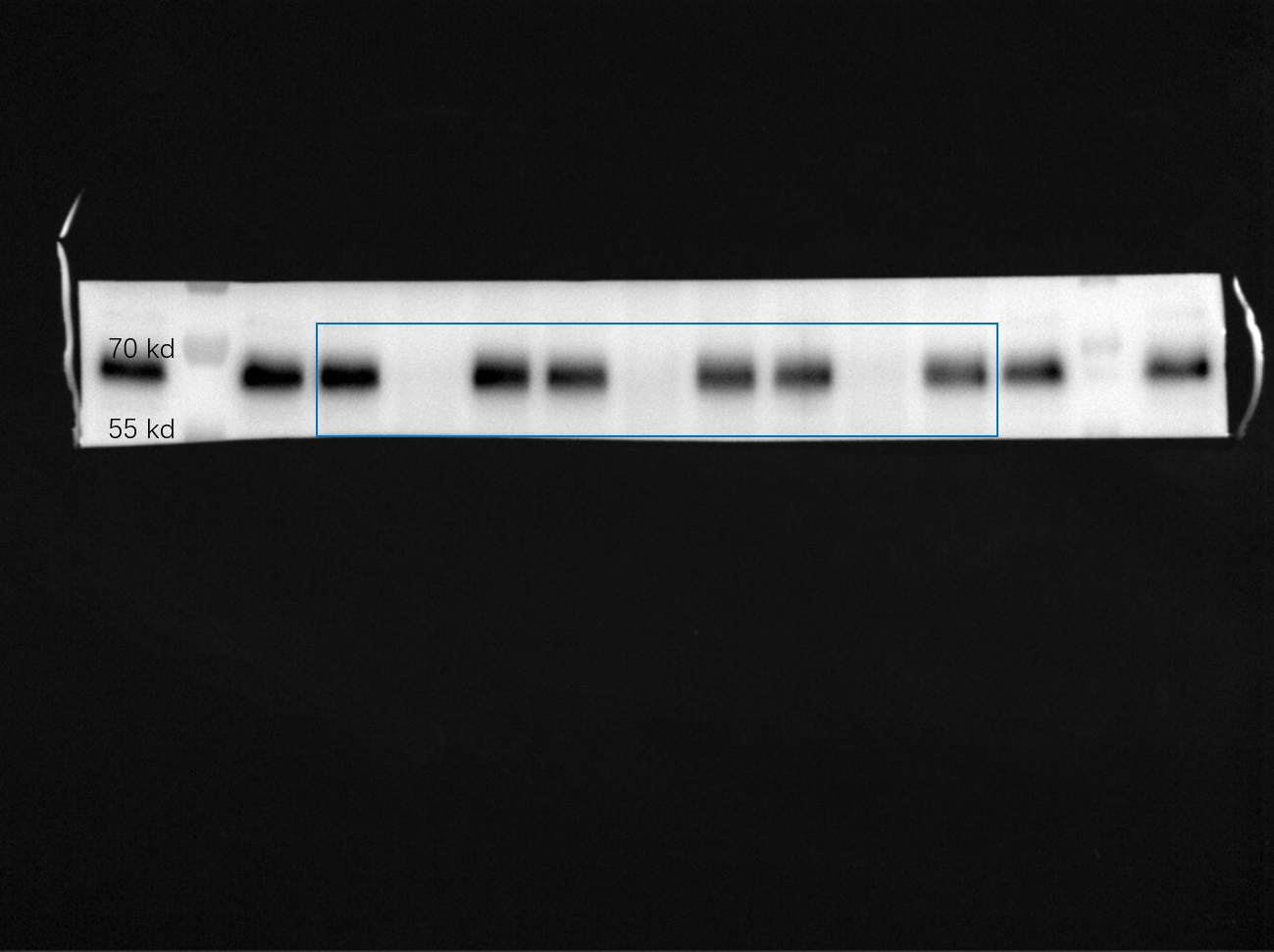

Supplement: Figure 3—figure supplement 3—source data 2. [file elife-89317-fig3-figsupp3-data2.zip › syt labelled.tif]

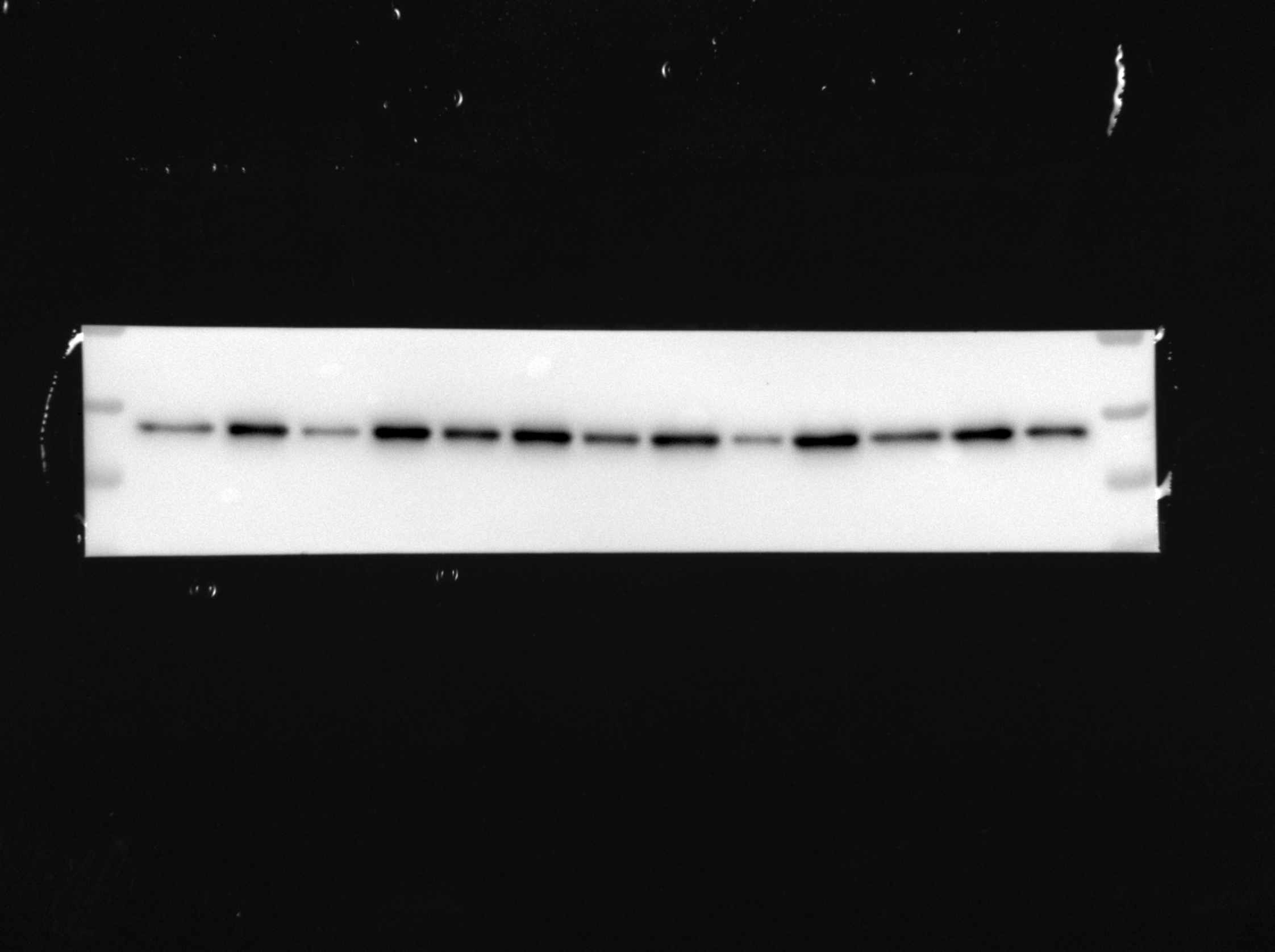

Supplement: Figure 8—source data 1. [file elife-89317-fig8-data1.zip › Figure 8-source data 1/GAPDH left.tif]

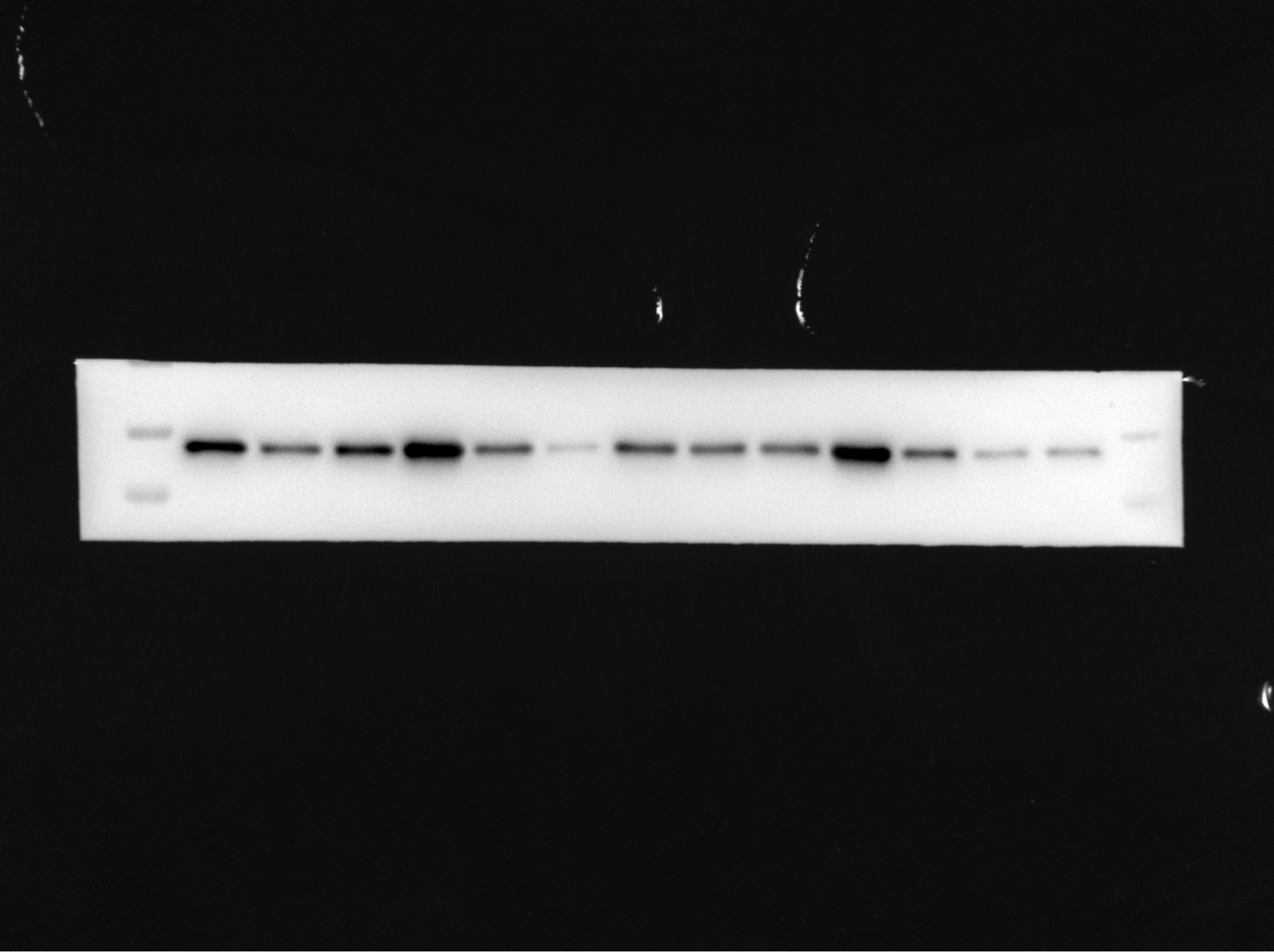

Supplement: Figure 8—source data 1. [file elife-89317-fig8-data1.zip › Figure 8-source data 1/GAPDH right.tif]

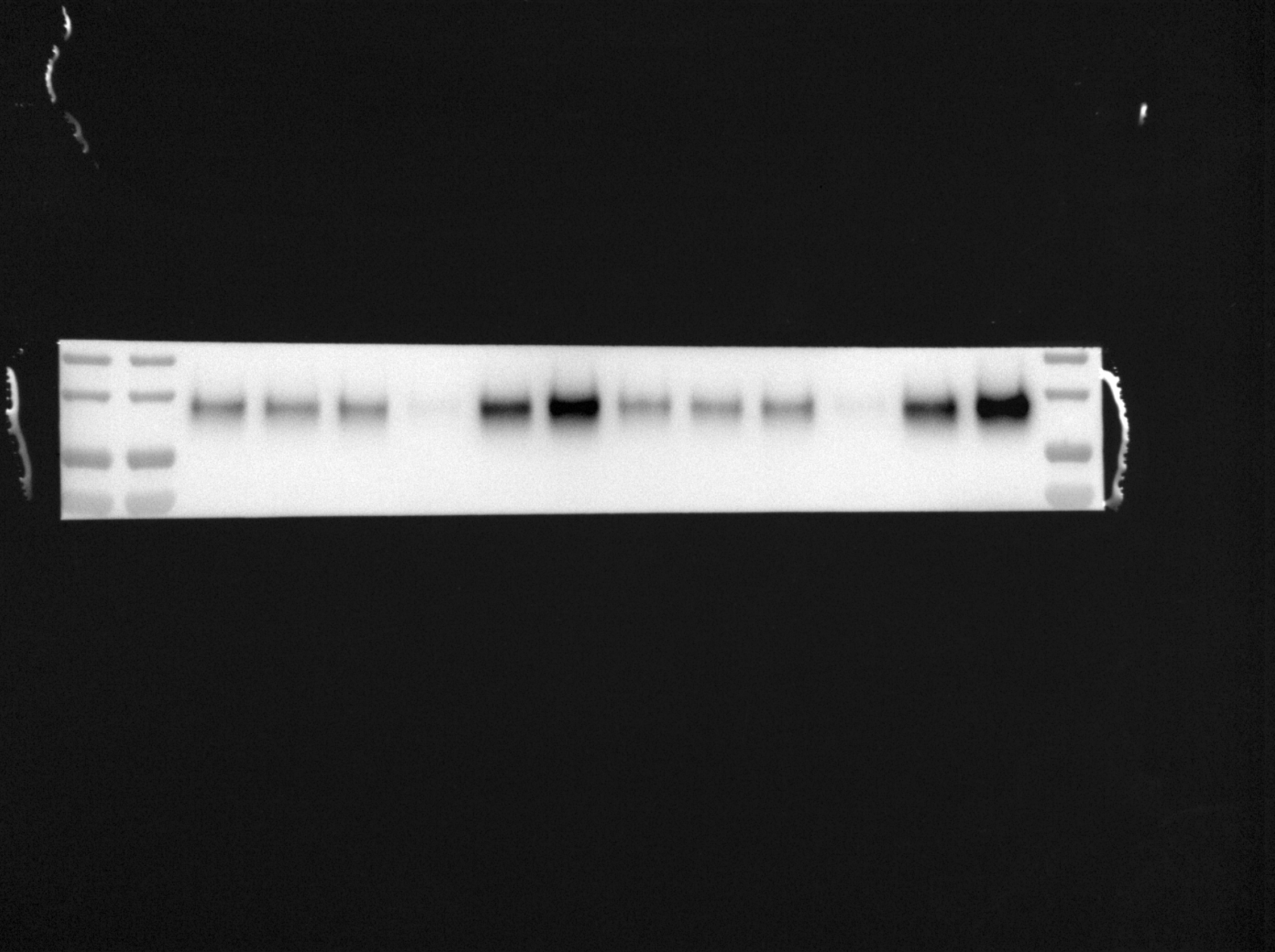

Supplement: Figure 8—source data 1. [file elife-89317-fig8-data1.zip › Figure 8-source data 1/GLUN1 left.tif]

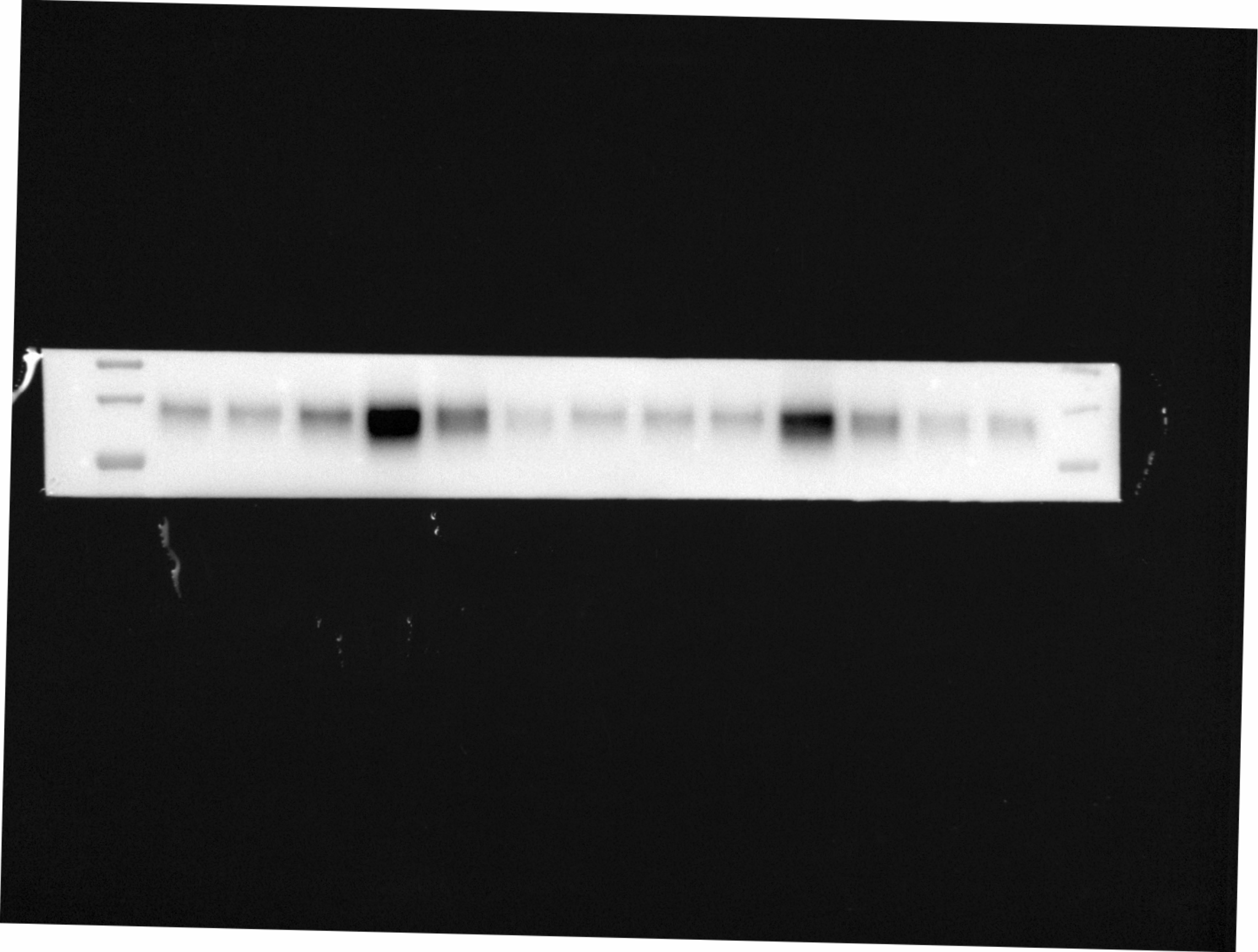

Supplement: Figure 8—source data 1. [file elife-89317-fig8-data1.zip › Figure 8-source data 1/GluN1 right original.tif]

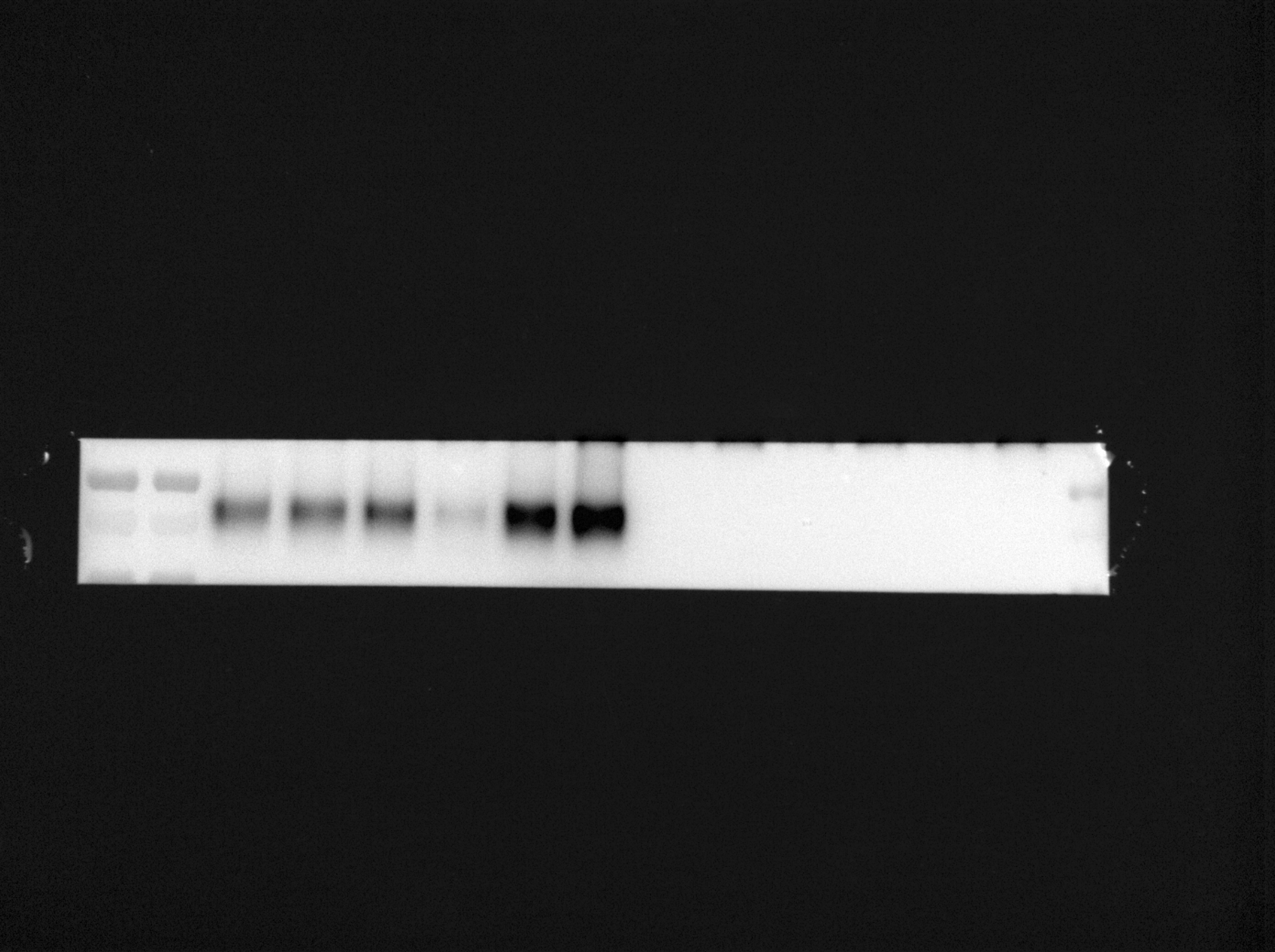

Supplement: Figure 8—source data 1. [file elife-89317-fig8-data1.zip › Figure 8-source data 1/HA left OG.tif]

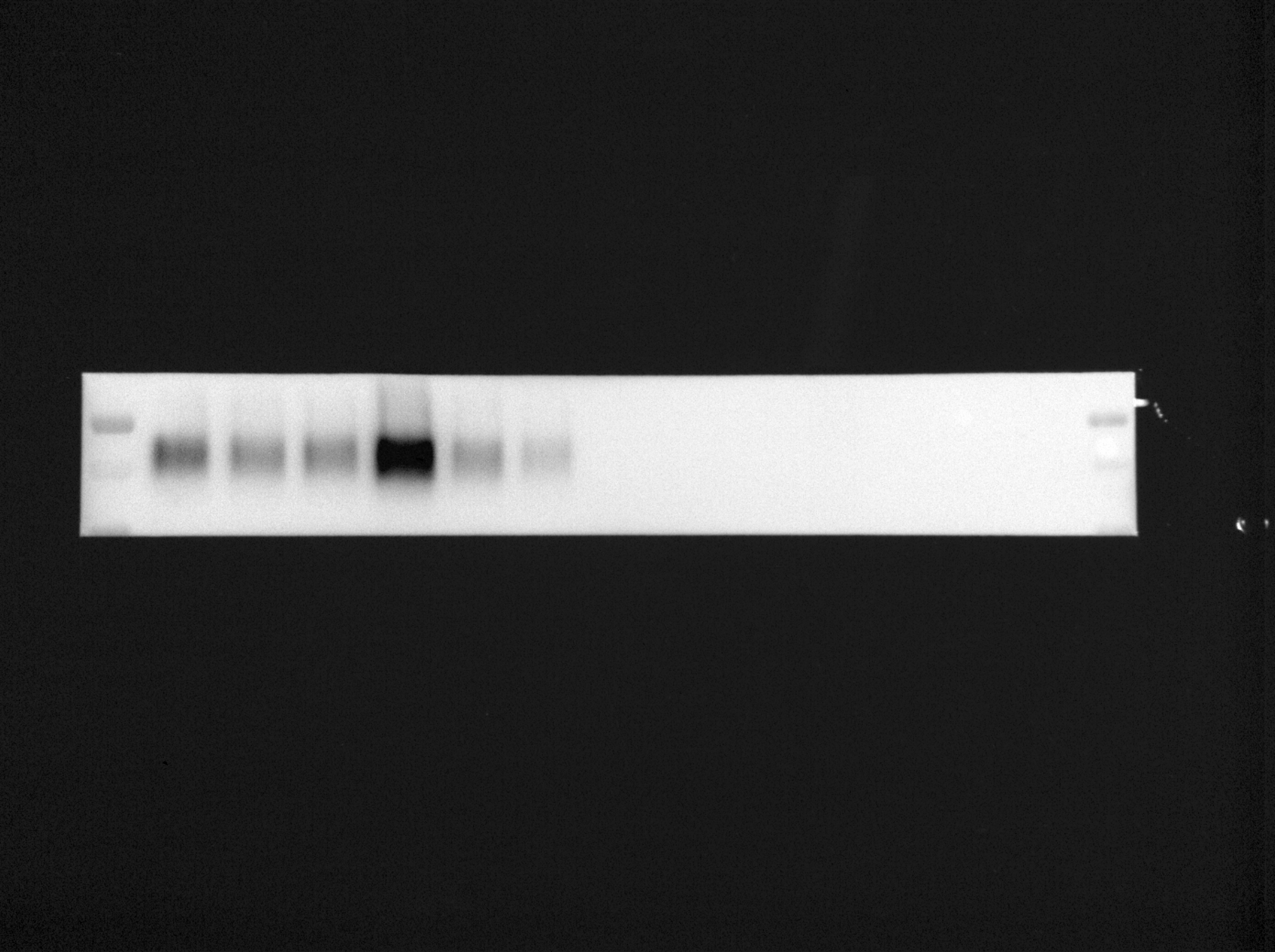

Supplement: Figure 8—source data 1. [file elife-89317-fig8-data1.zip › Figure 8-source data 1/HA right OG.tif]

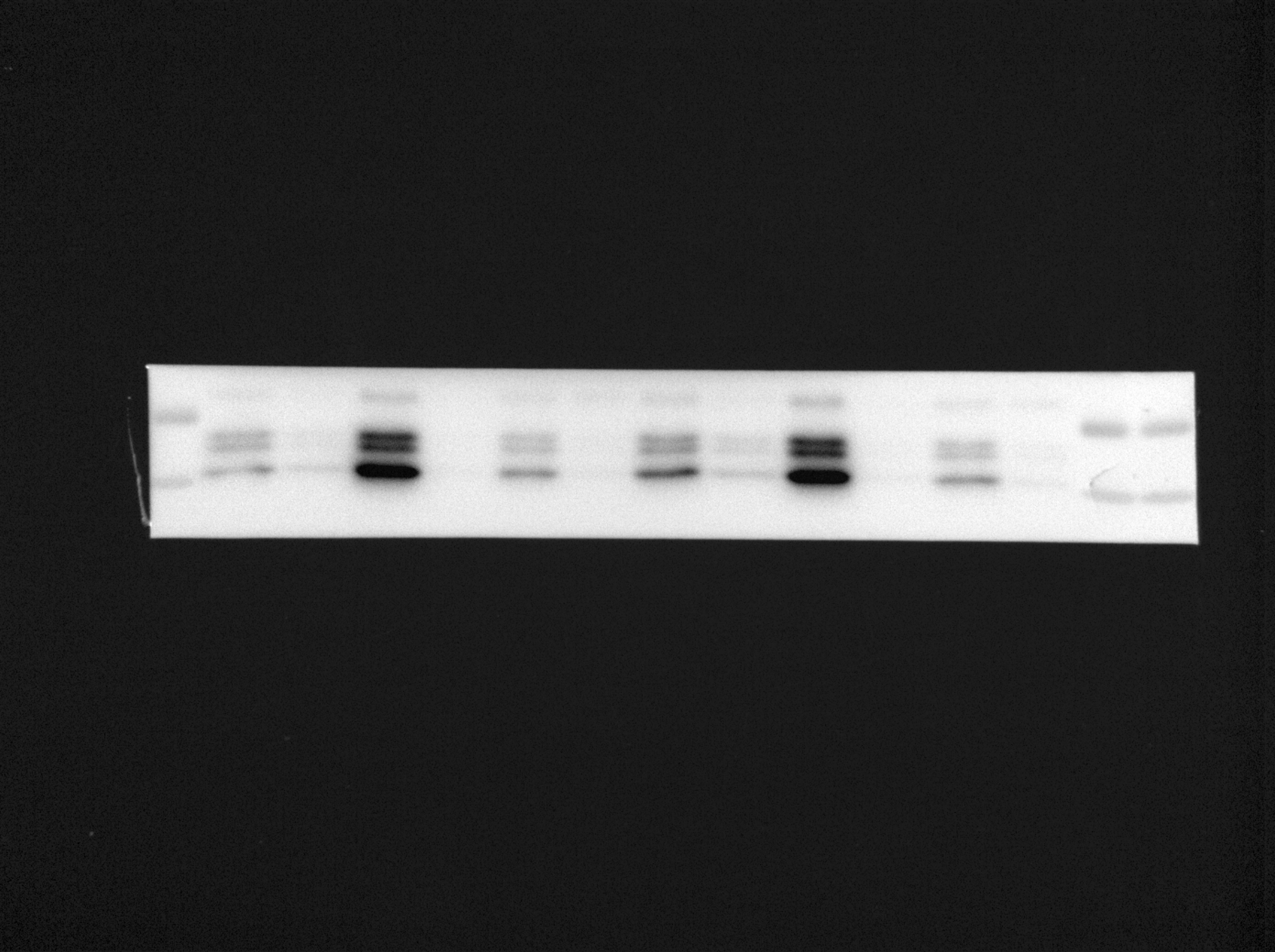

Supplement: Figure 8—source data 1. [file elife-89317-fig8-data1.zip › Figure 8-source data 1/MBP Left.tif]

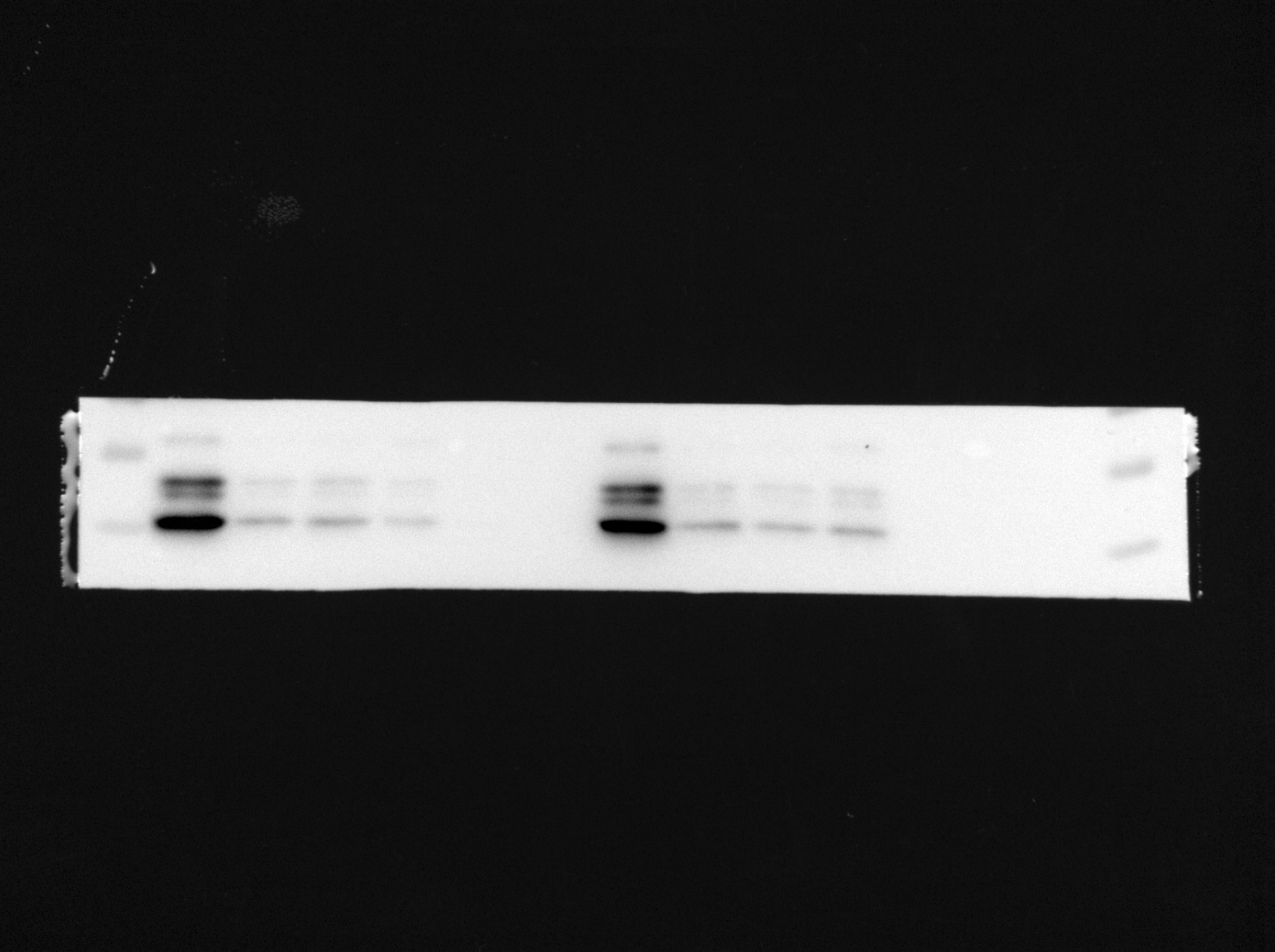

Supplement: Figure 8—source data 1. [file elife-89317-fig8-data1.zip › Figure 8-source data 1/MBP right.tif]

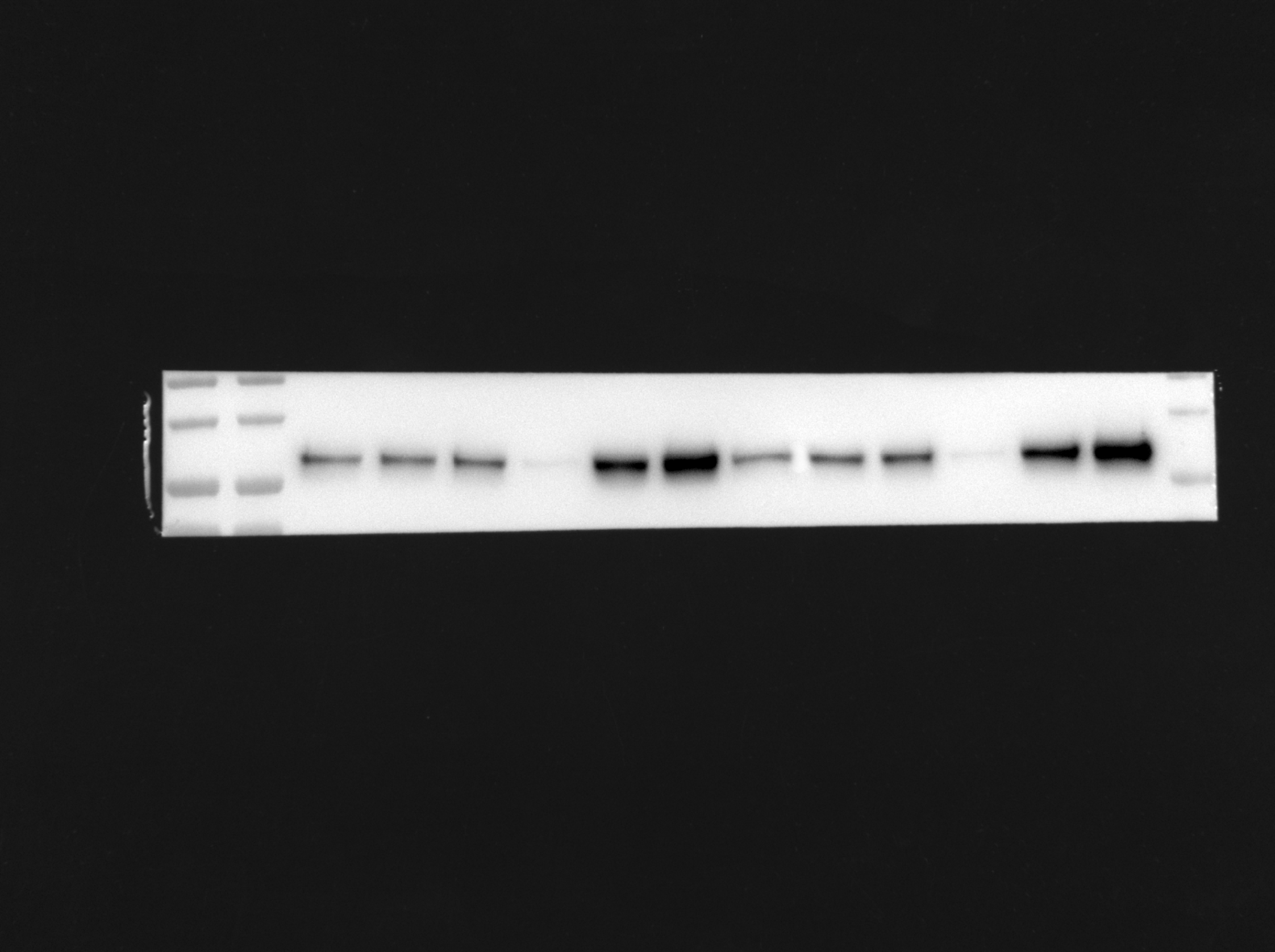

Supplement: Figure 8—source data 1. [file elife-89317-fig8-data1.zip › Figure 8-source data 1/NaK ATPase left original.tif]

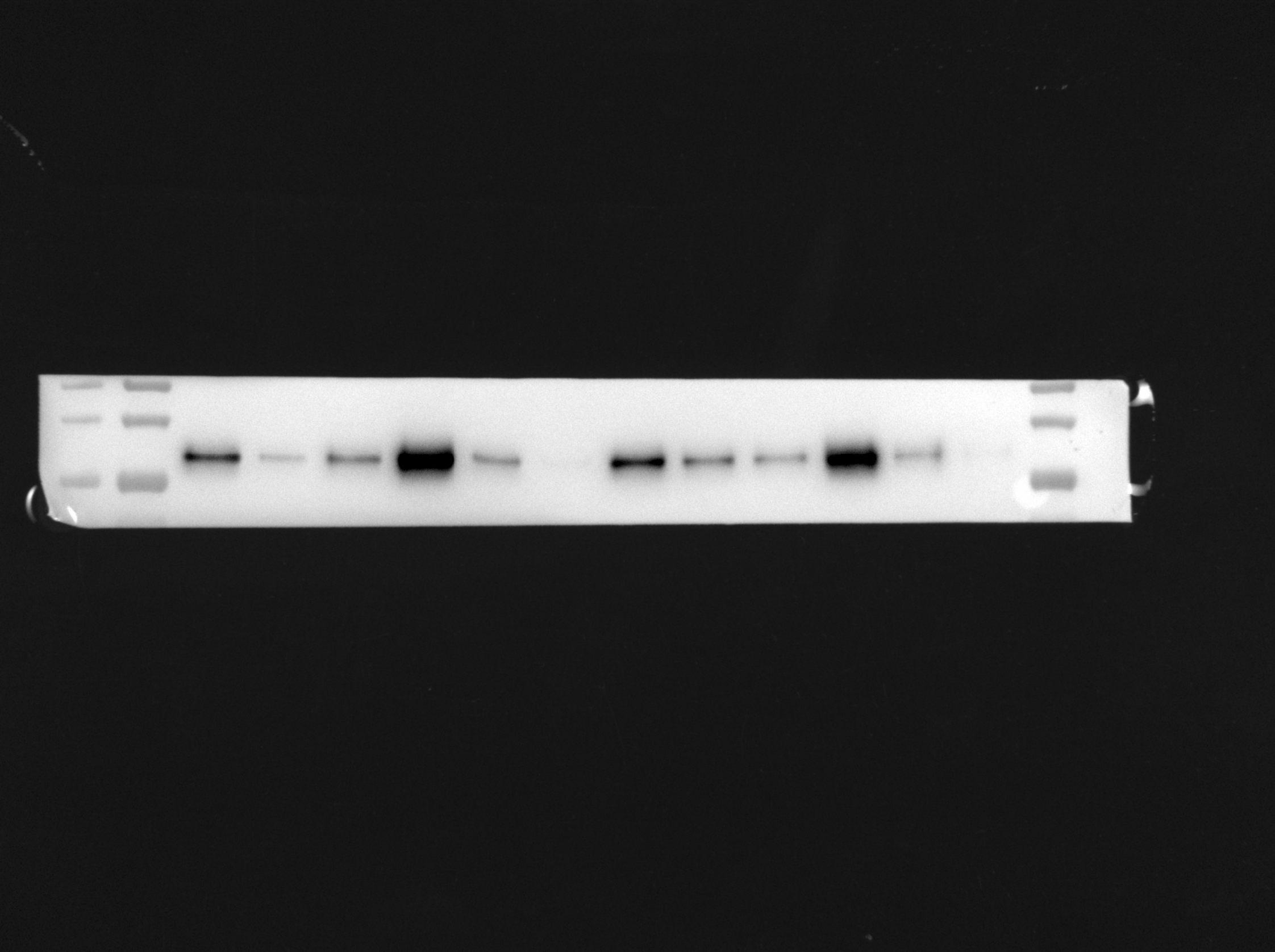

Supplement: Figure 8—source data 1. [file elife-89317-fig8-data1.zip › Figure 8-source data 1/NaK ATPase right original.tif]

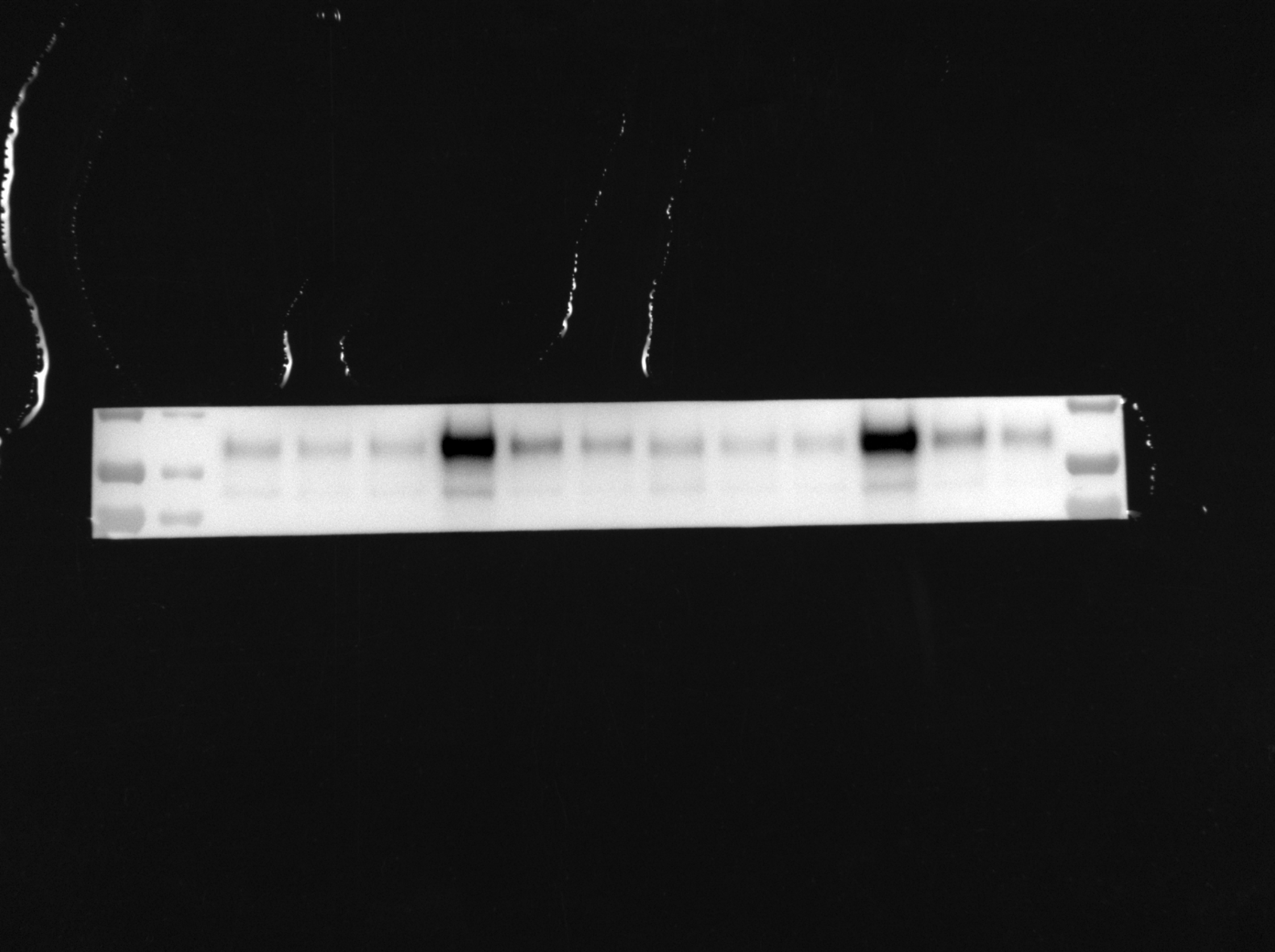

Supplement: Figure 8—source data 1. [file elife-89317-fig8-data1.zip › Figure 8-source data 1/PSD 95 right original.tif]

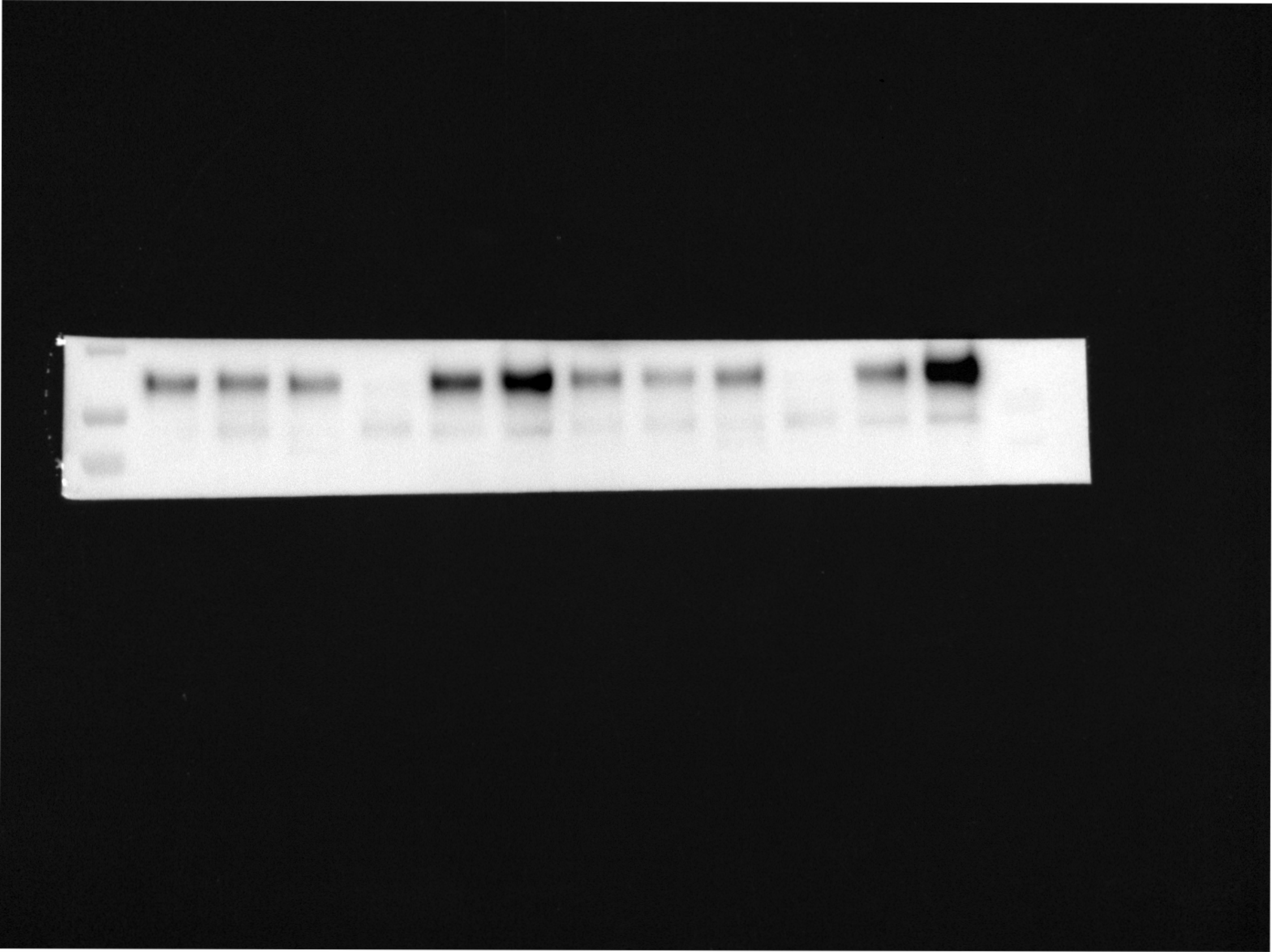

Supplement: Figure 8—source data 1. [file elife-89317-fig8-data1.zip › Figure 8-source data 1/PSD95 left original.tif]

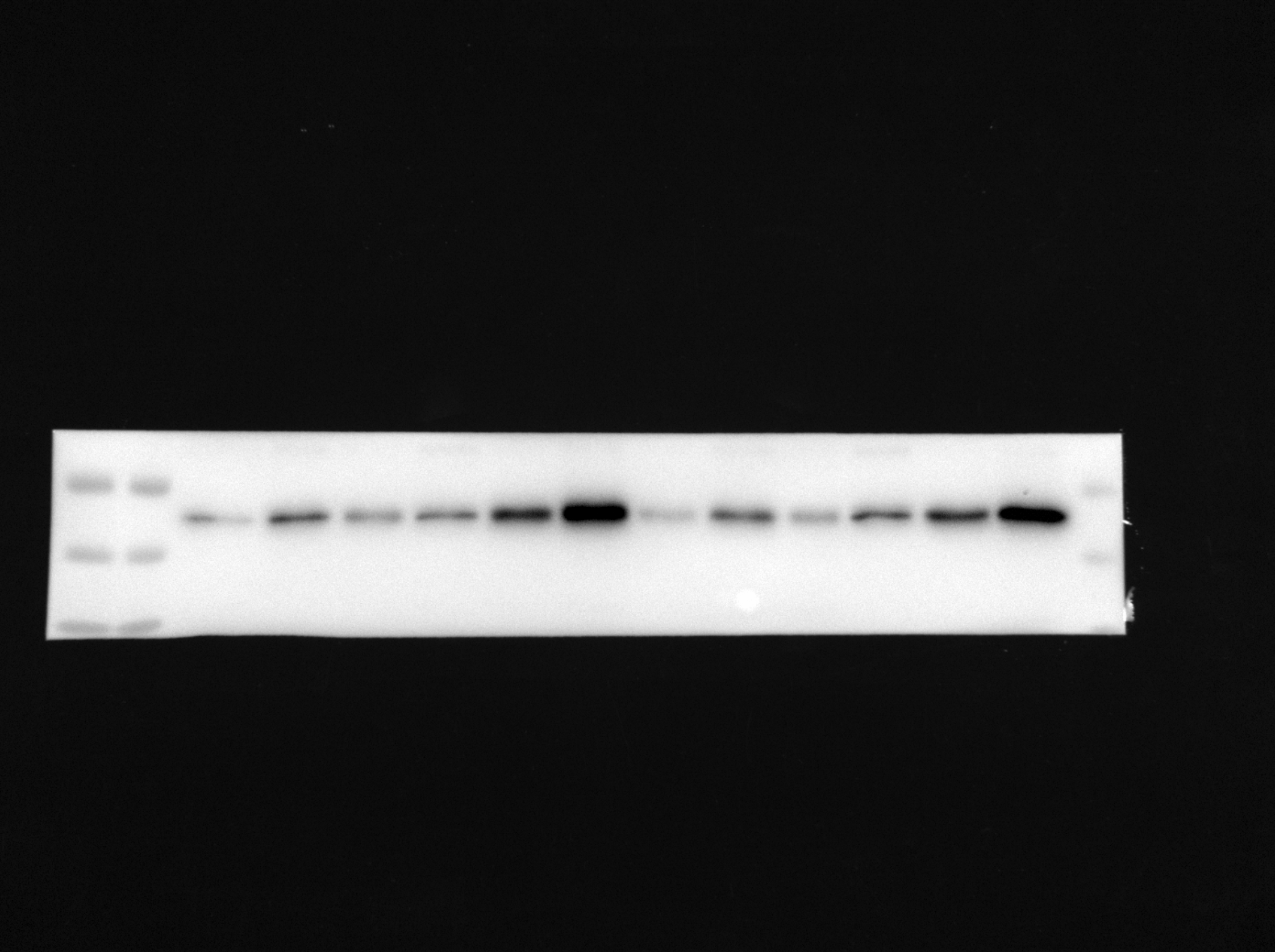

Supplement: Figure 8—source data 1. [file elife-89317-fig8-data1.zip › Figure 8-source data 1/SNAP23 left OG.tif]

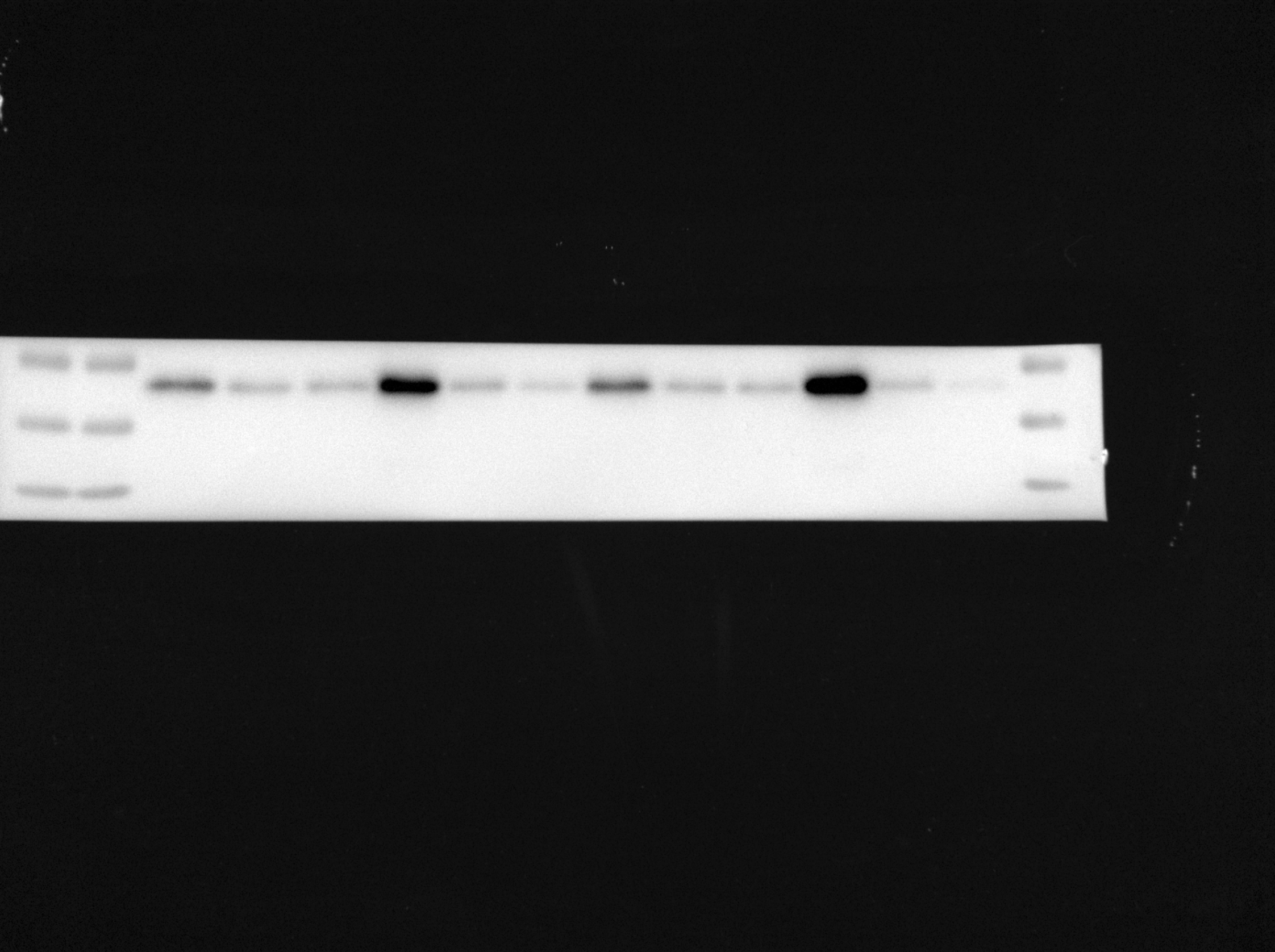

Supplement: Figure 8—source data 1. [file elife-89317-fig8-data1.zip › Figure 8-source data 1/SNAP23 right OG.tif]

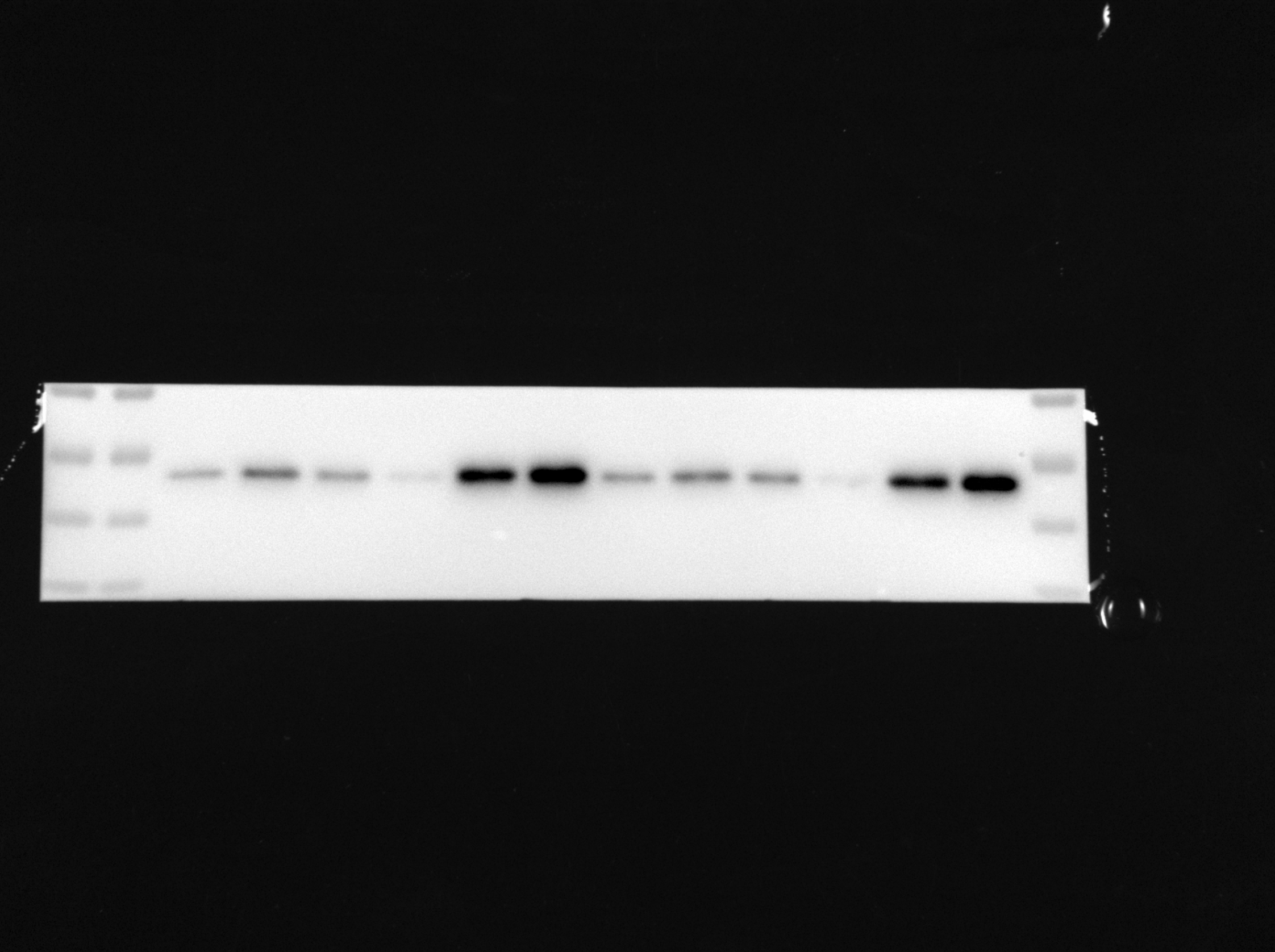

Supplement: Figure 8—source data 1. [file elife-89317-fig8-data1.zip › Figure 8-source data 1/SNAP25 left original.tif]

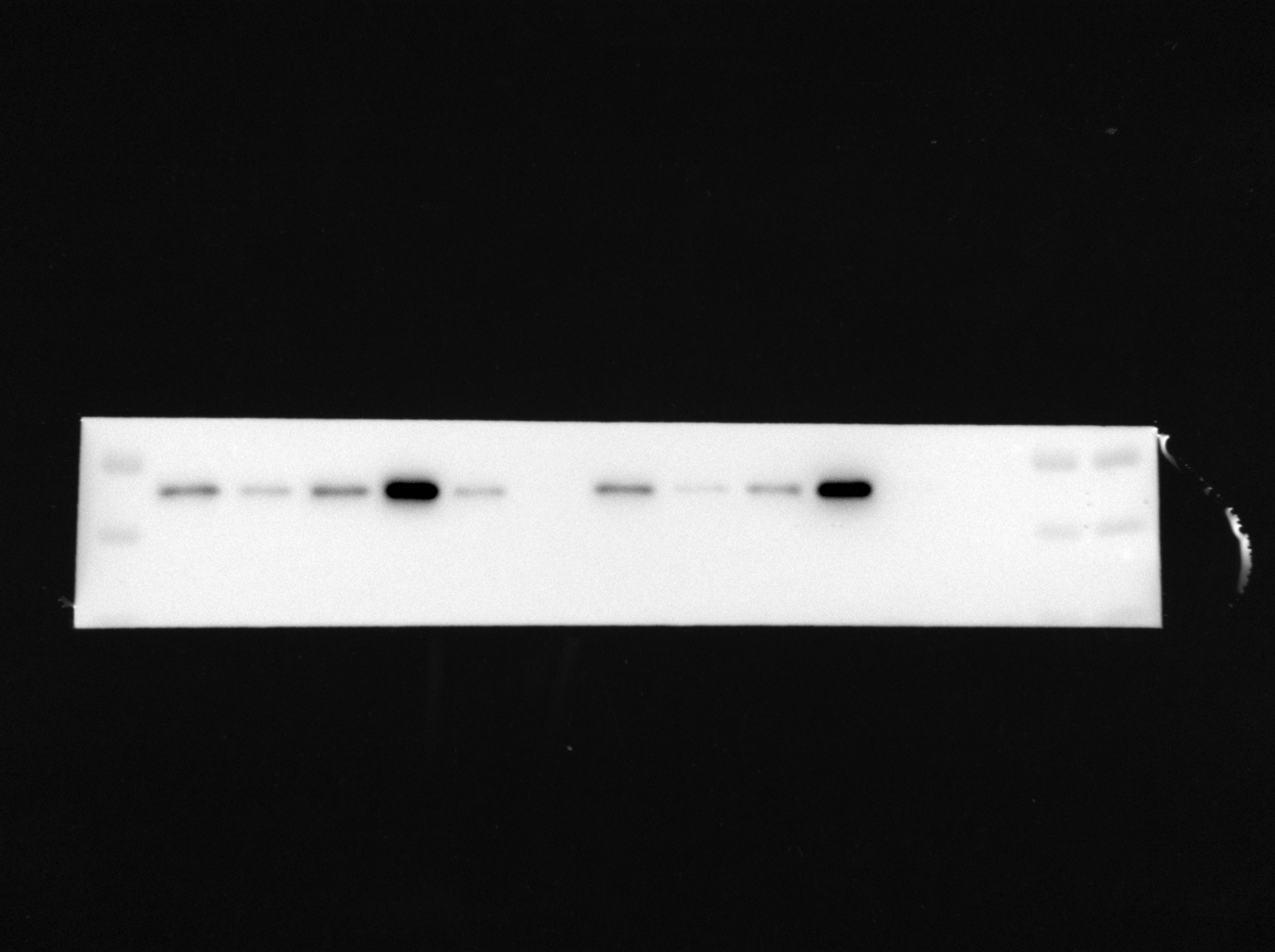

Supplement: Figure 8—source data 1. [file elife-89317-fig8-data1.zip › Figure 8-source data 1/SNAP25 right original.tif]

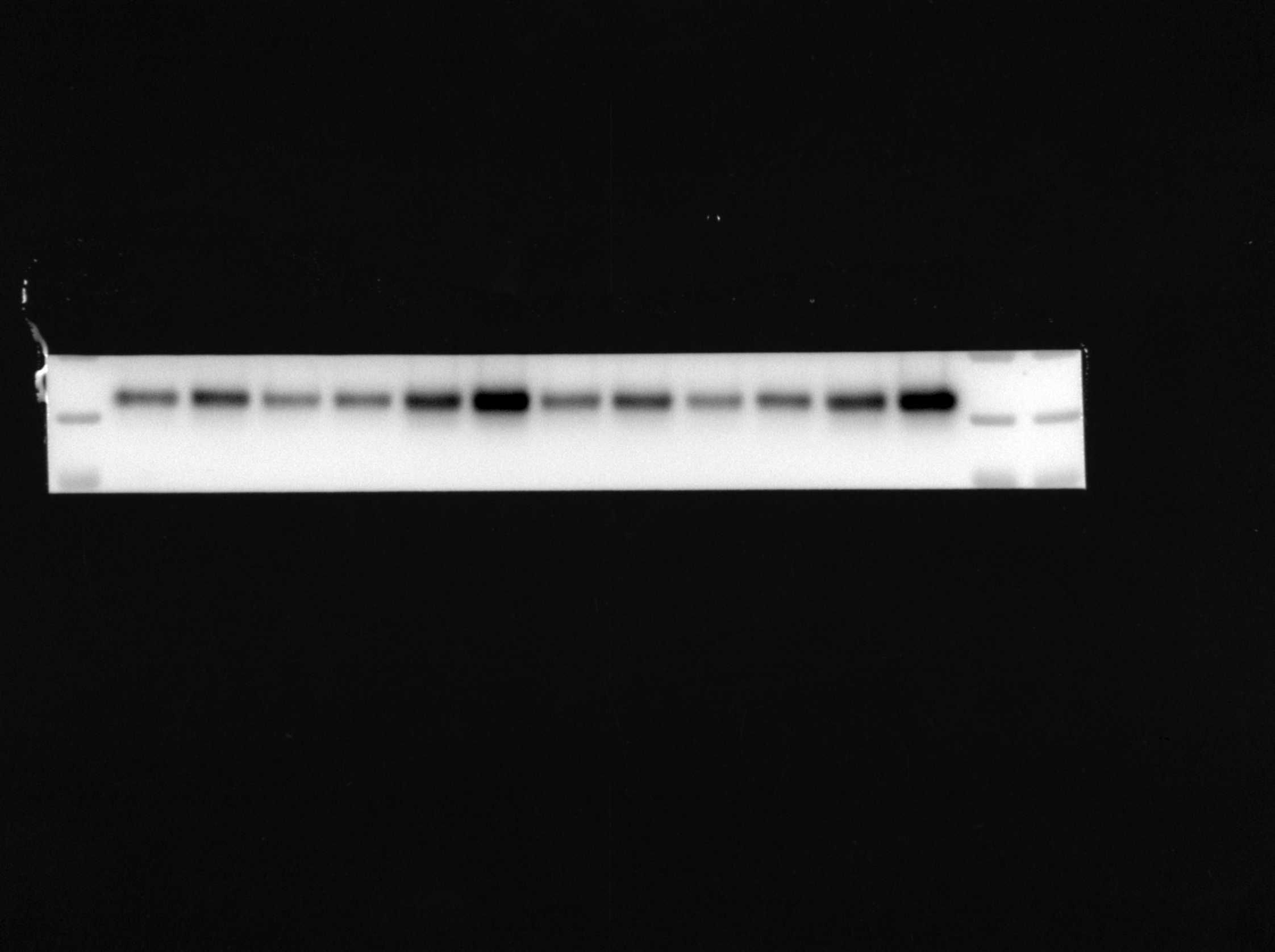

Supplement: Figure 8—source data 1. [file elife-89317-fig8-data1.zip › Figure 8-source data 1/syp left original.tif]

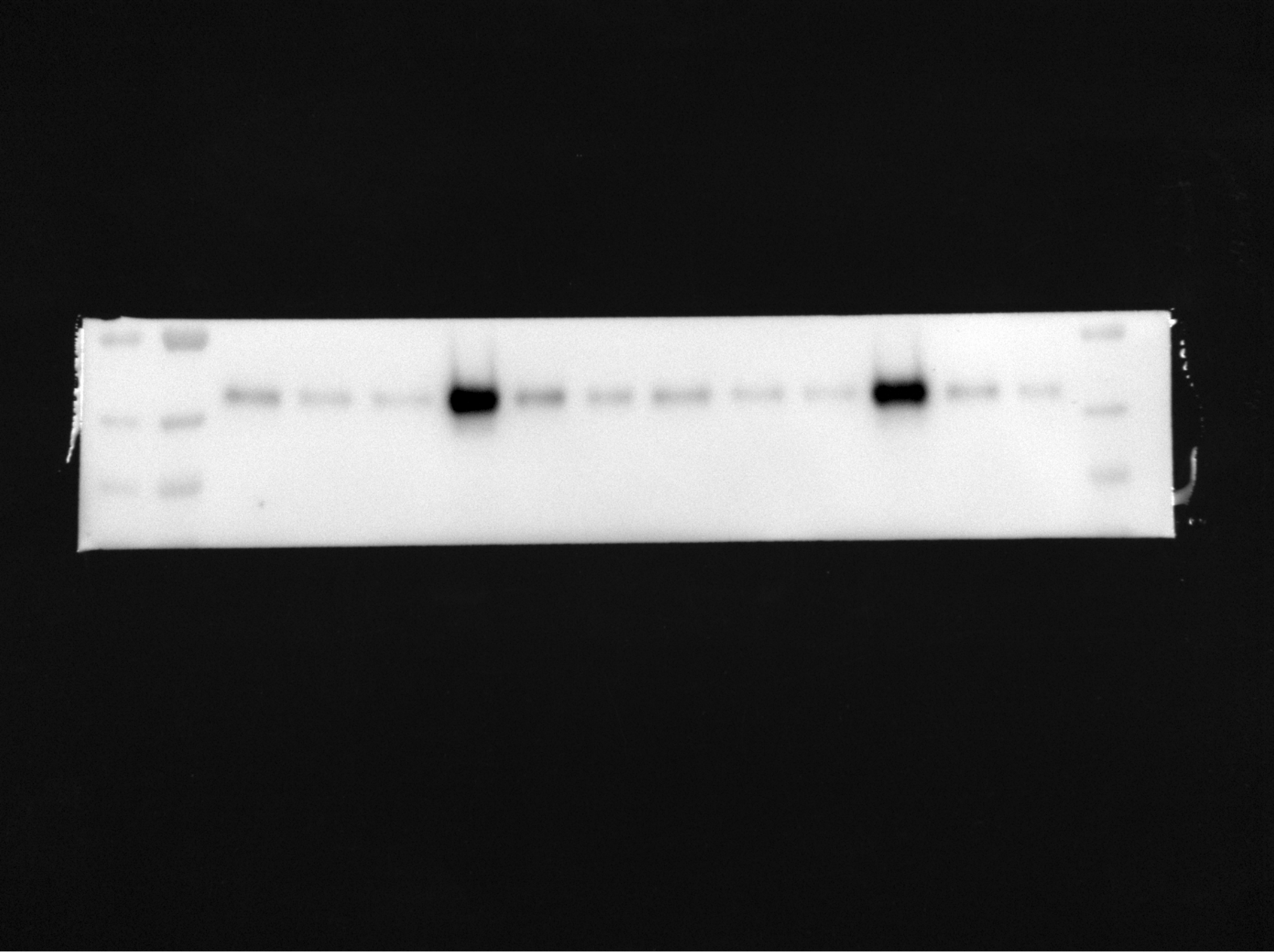

Supplement: Figure 8—source data 1. [file elife-89317-fig8-data1.zip › Figure 8-source data 1/syp right original.tif]

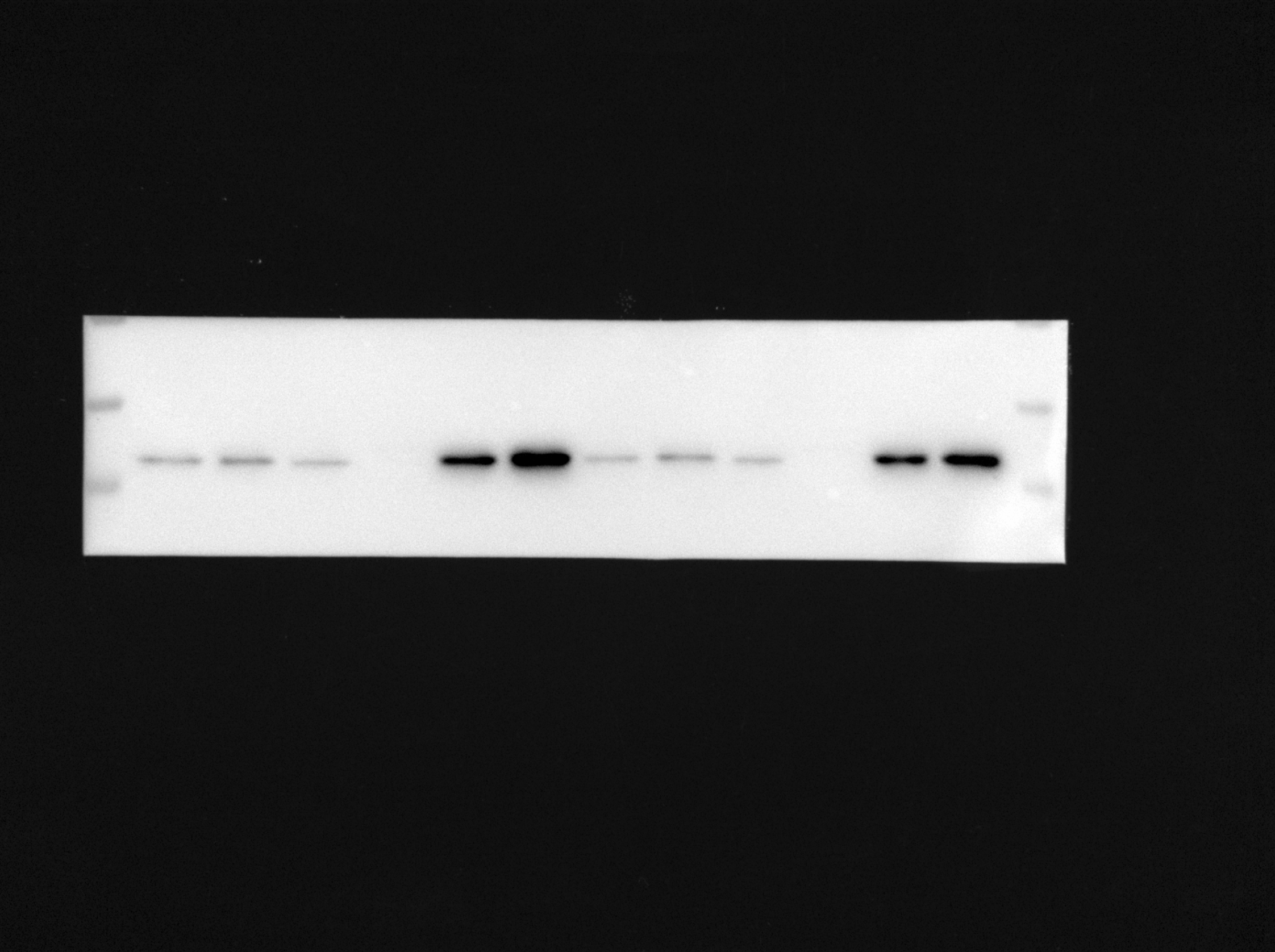

Supplement: Figure 8—source data 1. [file elife-89317-fig8-data1.zip › Figure 8-source data 1/VDAC left.tif]

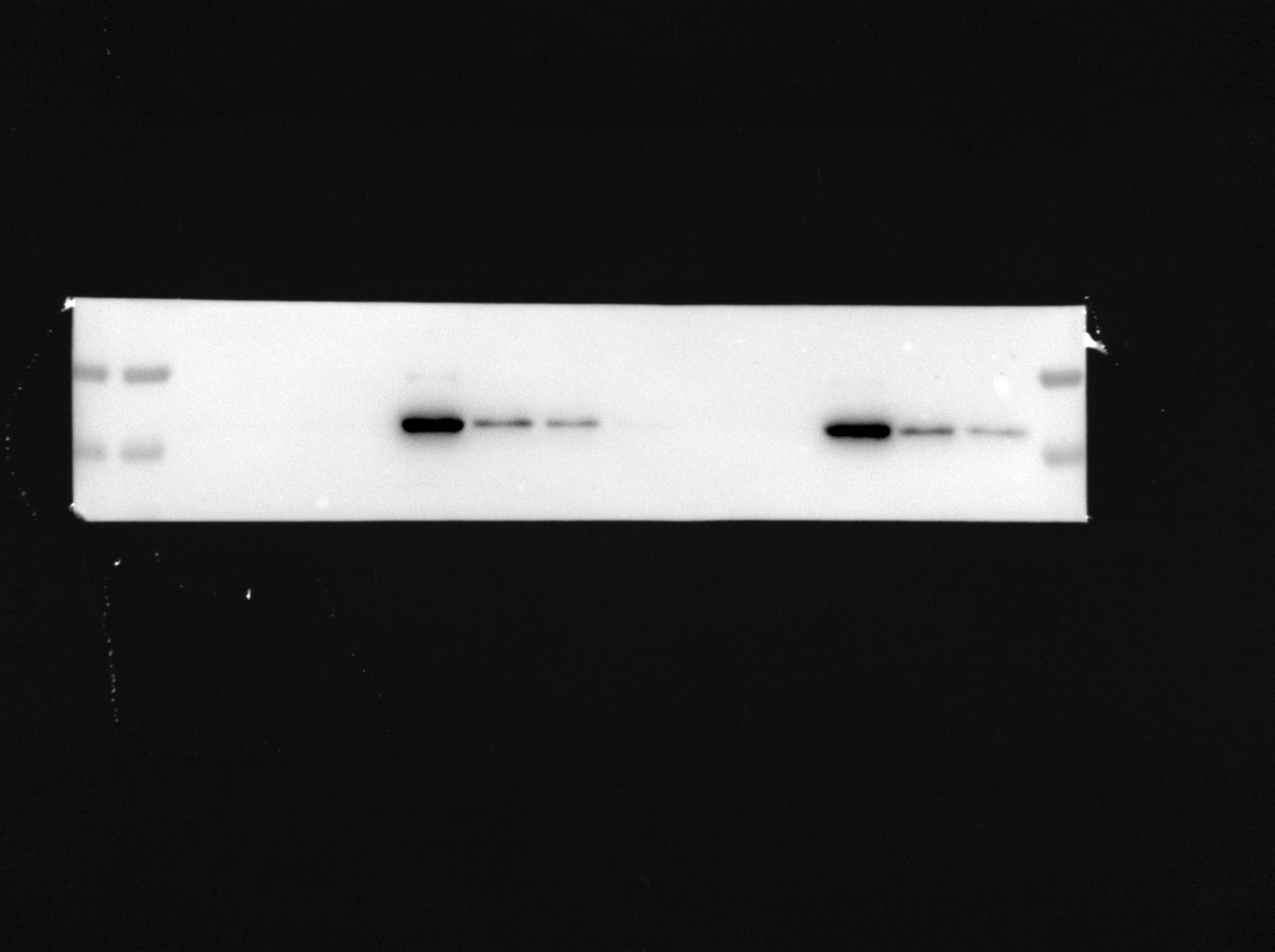

Supplement: Figure 8—source data 1. [file elife-89317-fig8-data1.zip › Figure 8-source data 1/VDAC right.tif]

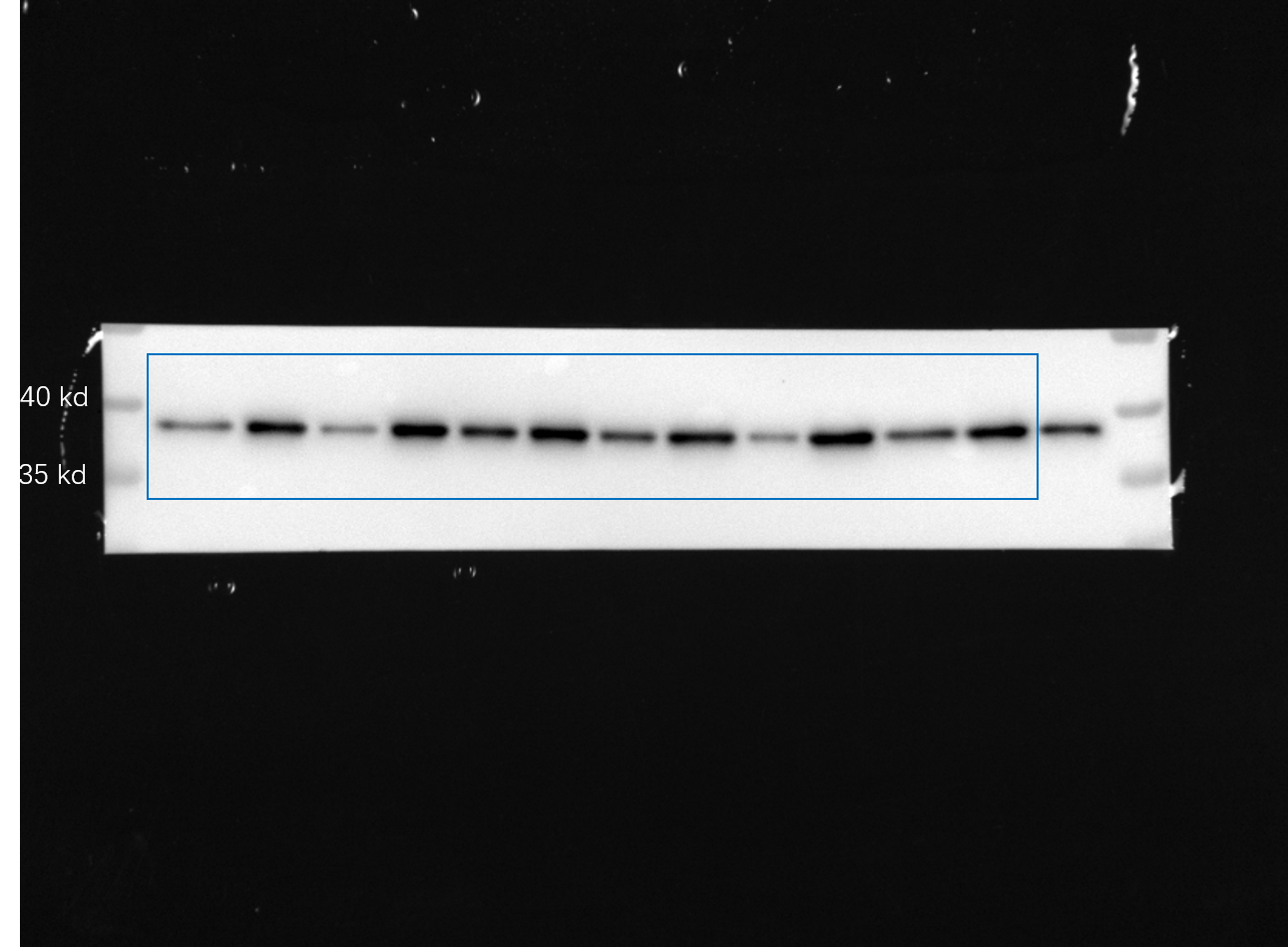

Supplement: Figure 8—source data 2. [file elife-89317-fig8-data2.zip › Figure 8-source data 2/GAPDH left labelled.tif]

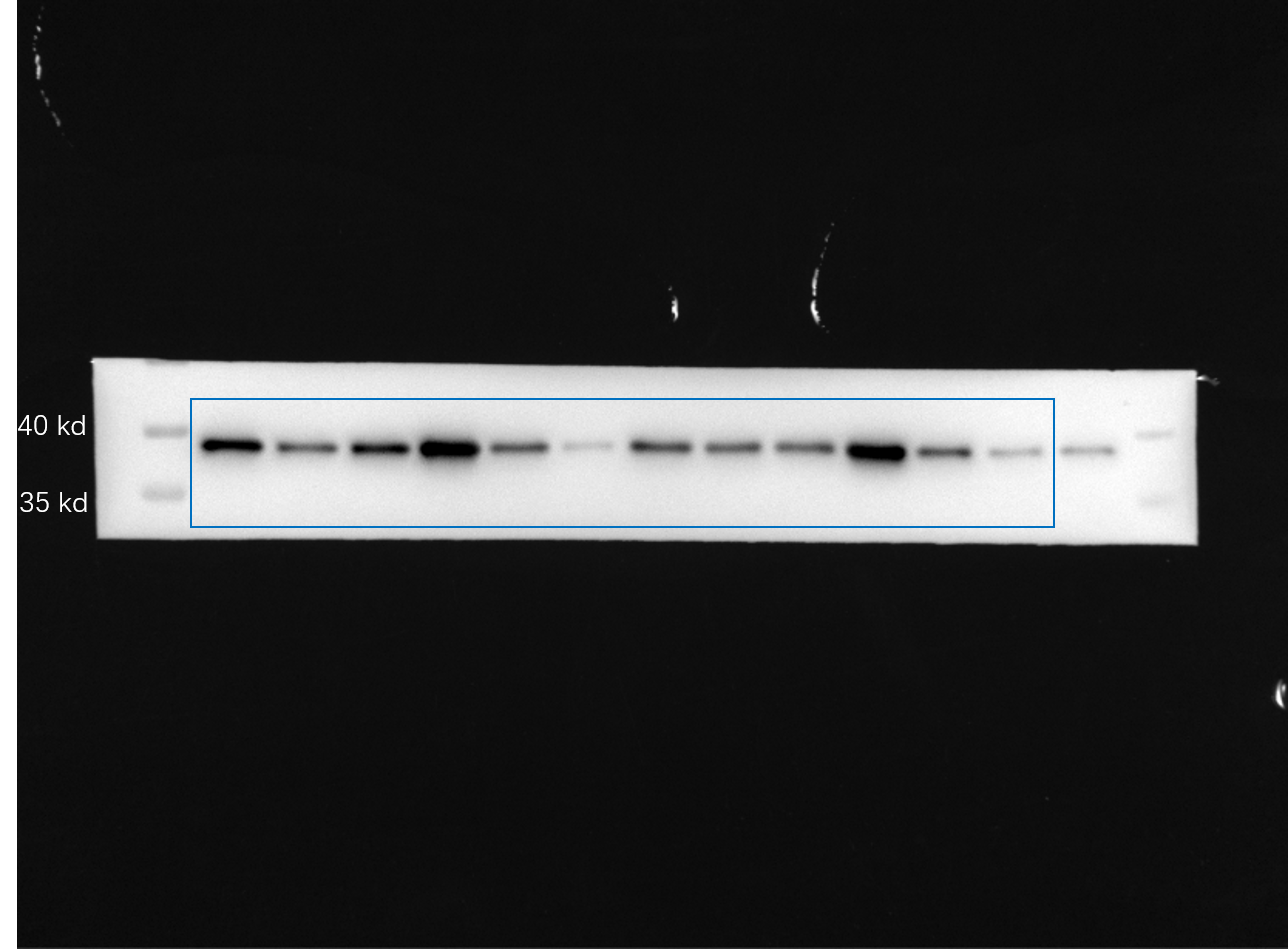

Supplement: Figure 8—source data 2. [file elife-89317-fig8-data2.zip › Figure 8-source data 2/GAPDH right labelled.tif]

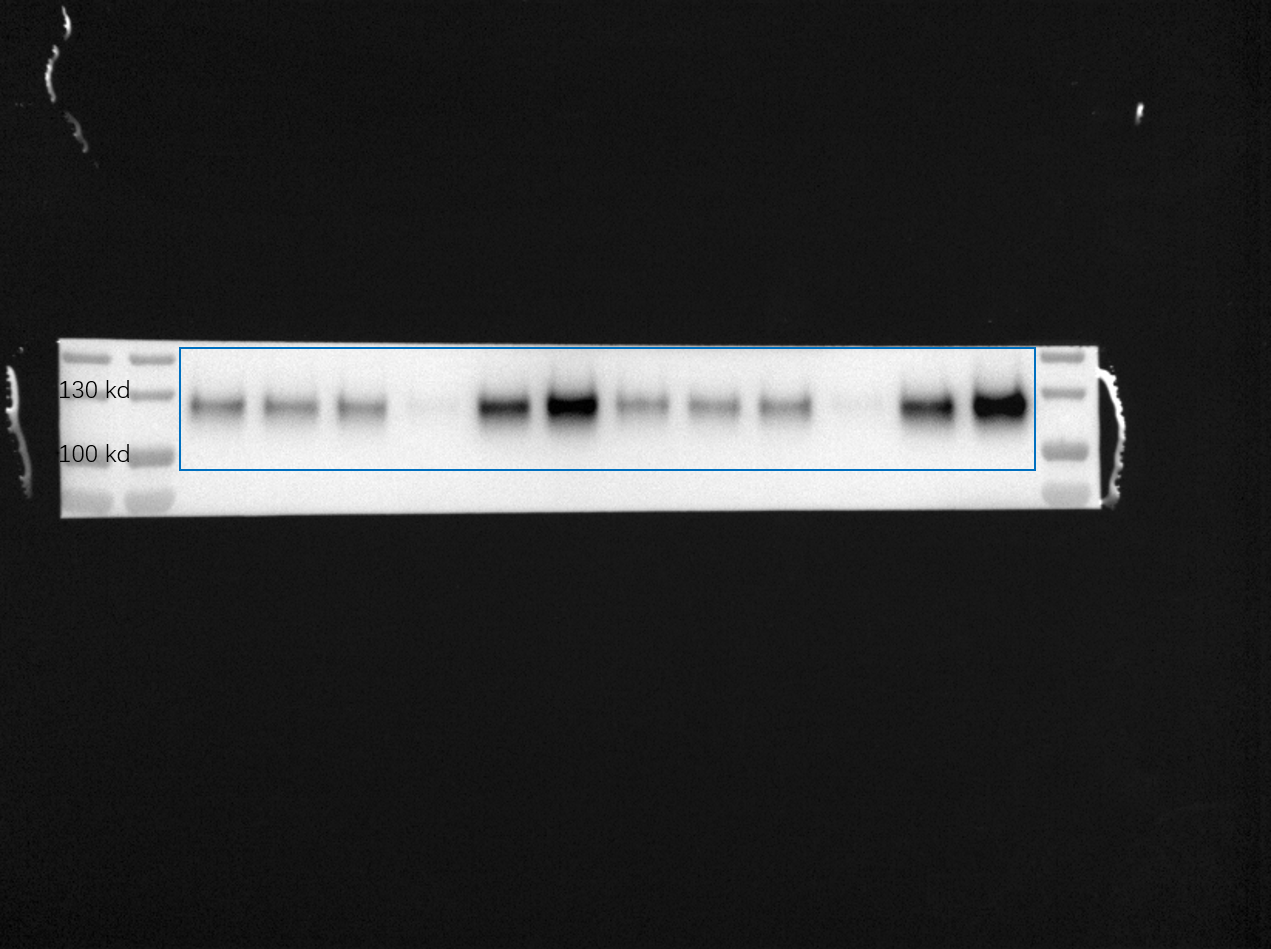

Supplement: Figure 8—source data 2. [file elife-89317-fig8-data2.zip › Figure 8-source data 2/GLUN1 left labelled.tif]

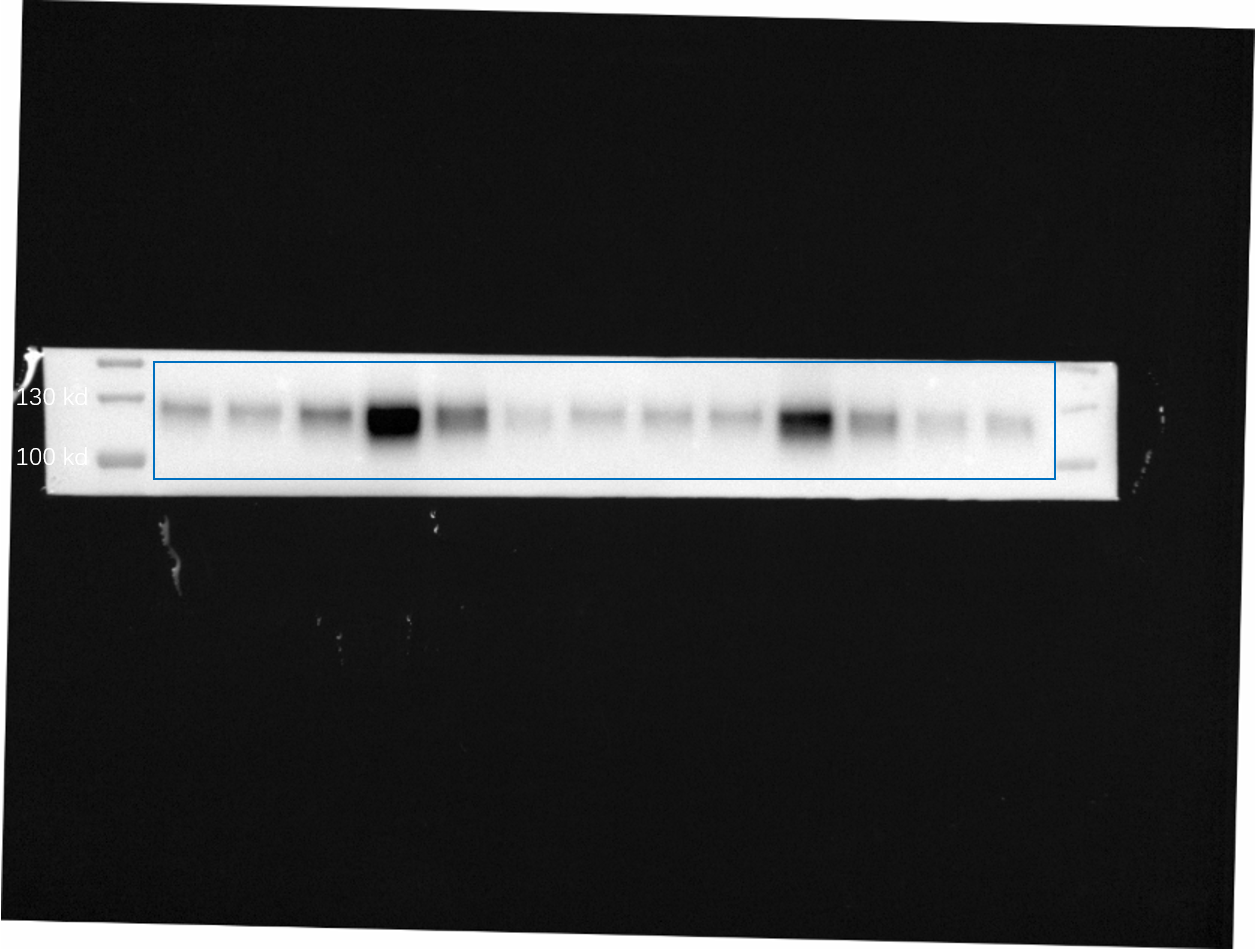

Supplement: Figure 8—source data 2. [file elife-89317-fig8-data2.zip › Figure 8-source data 2/GluN1 right labelled.tif]

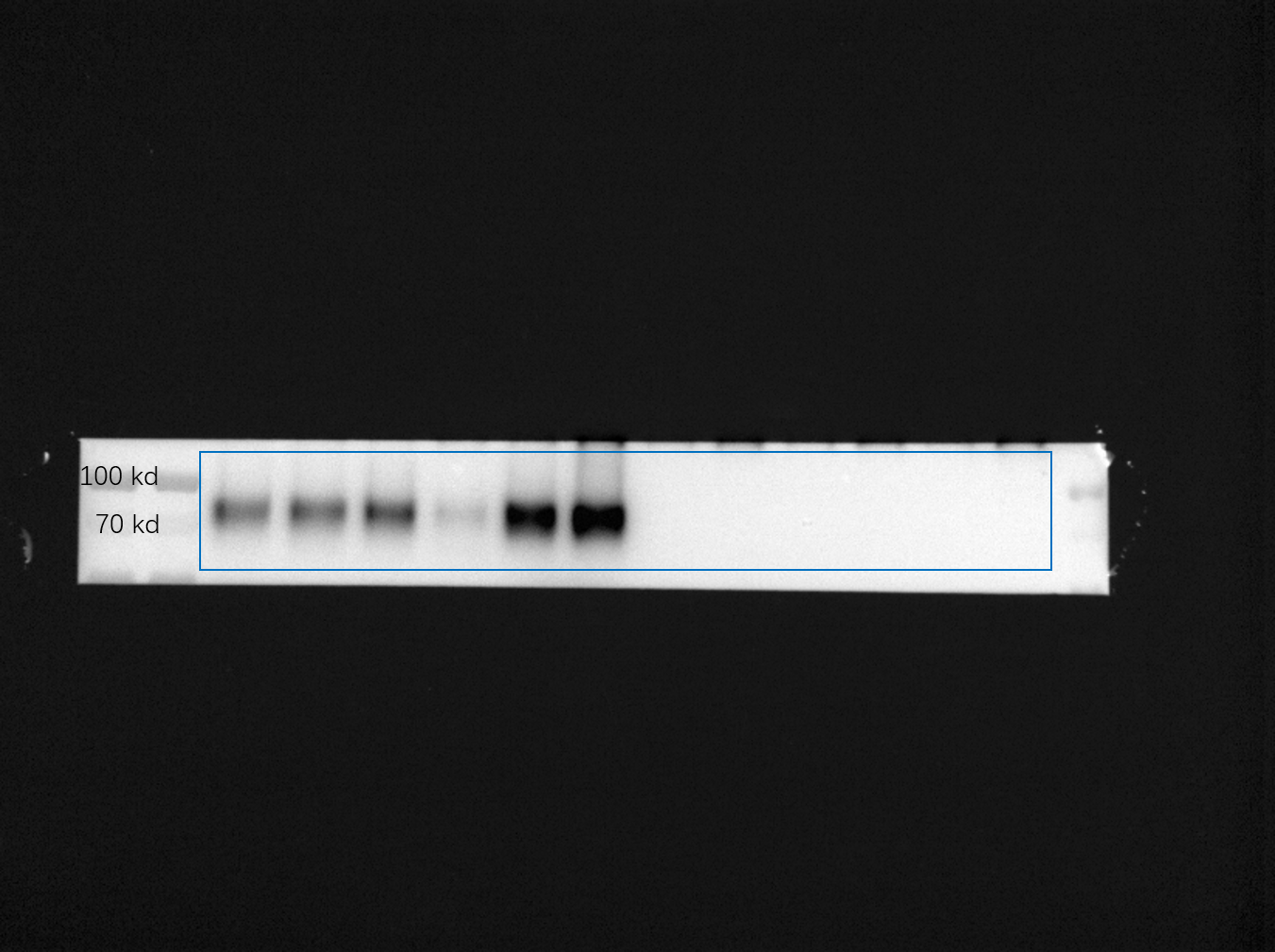

Supplement: Figure 8—source data 2. [file elife-89317-fig8-data2.zip › Figure 8-source data 2/HA left labelled.tif]

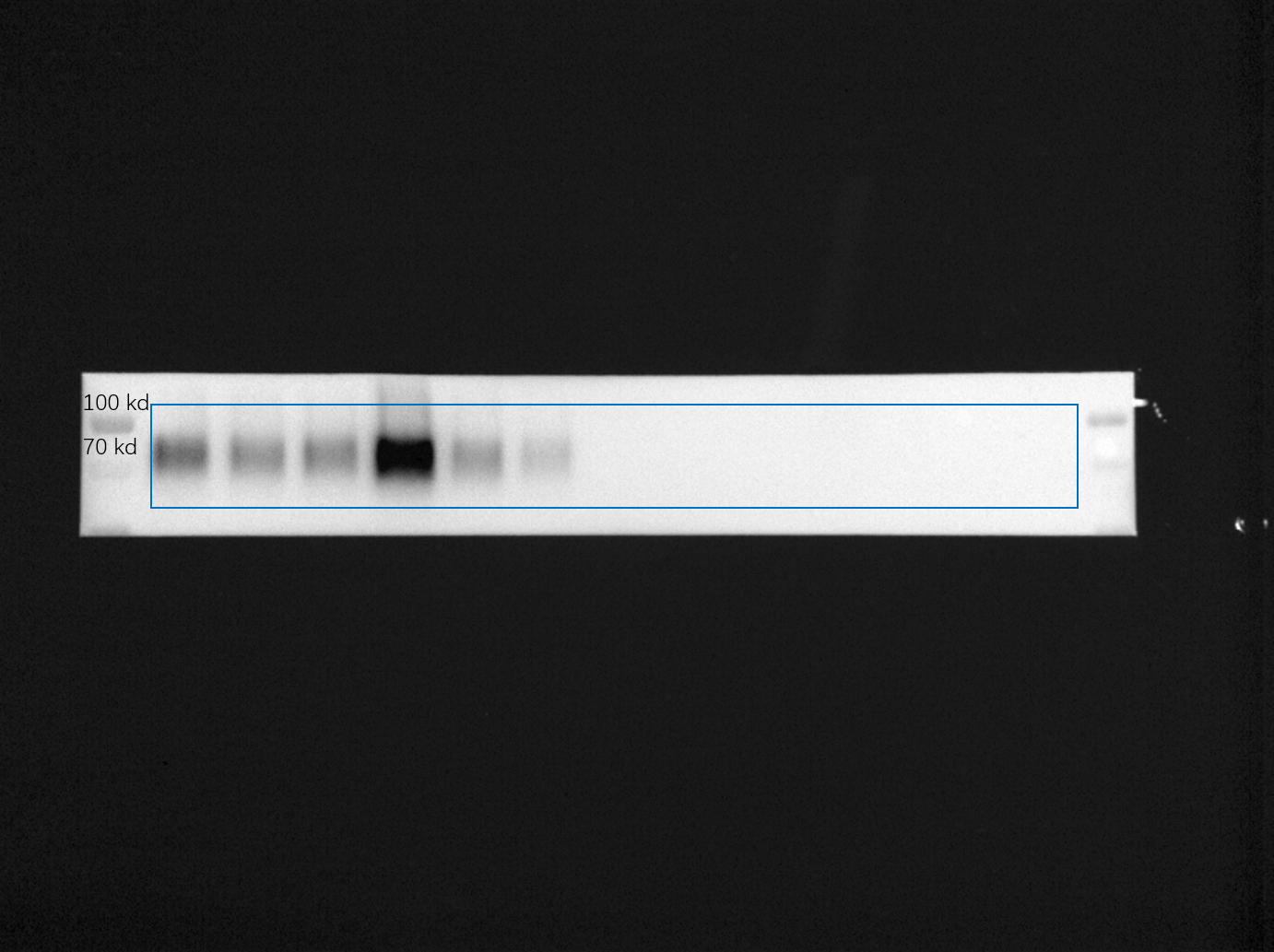

Supplement: Figure 8—source data 2. [file elife-89317-fig8-data2.zip › Figure 8-source data 2/HA right labelled.tif]

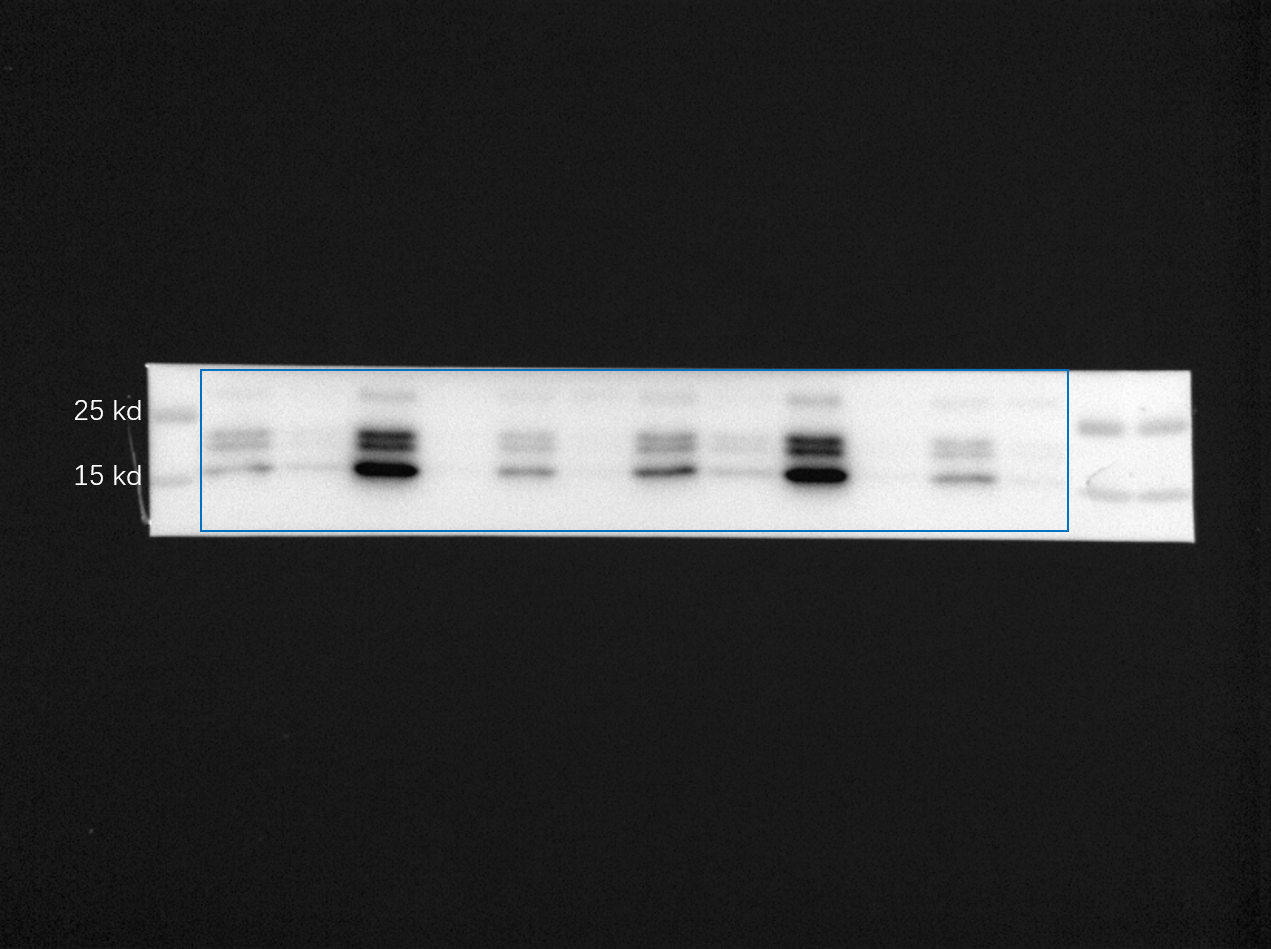

Supplement: Figure 8—source data 2. [file elife-89317-fig8-data2.zip › Figure 8-source data 2/MBP Left labelled.tif]

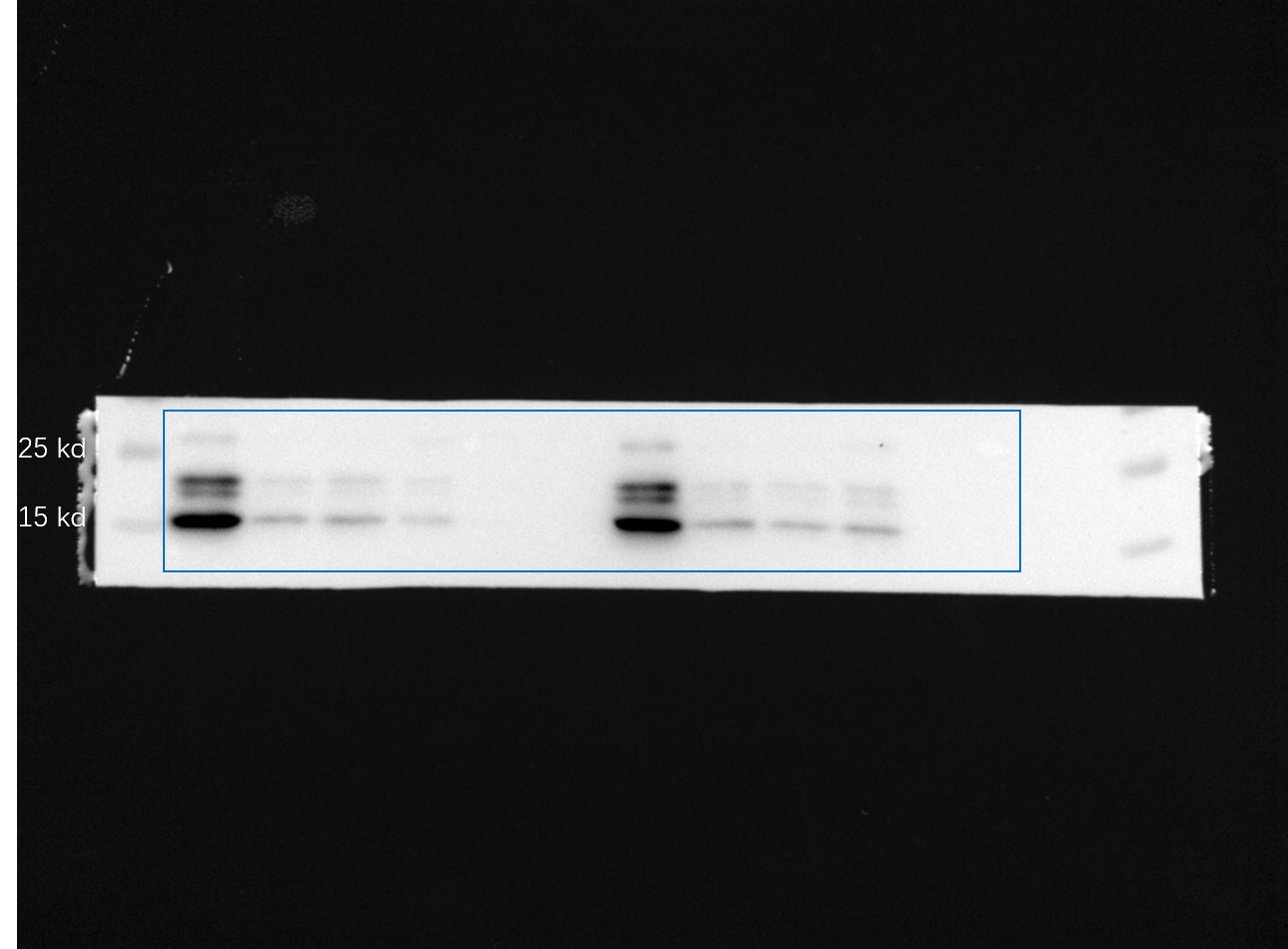

Supplement: Figure 8—source data 2. [file elife-89317-fig8-data2.zip › Figure 8-source data 2/MBP right labelled.tif]

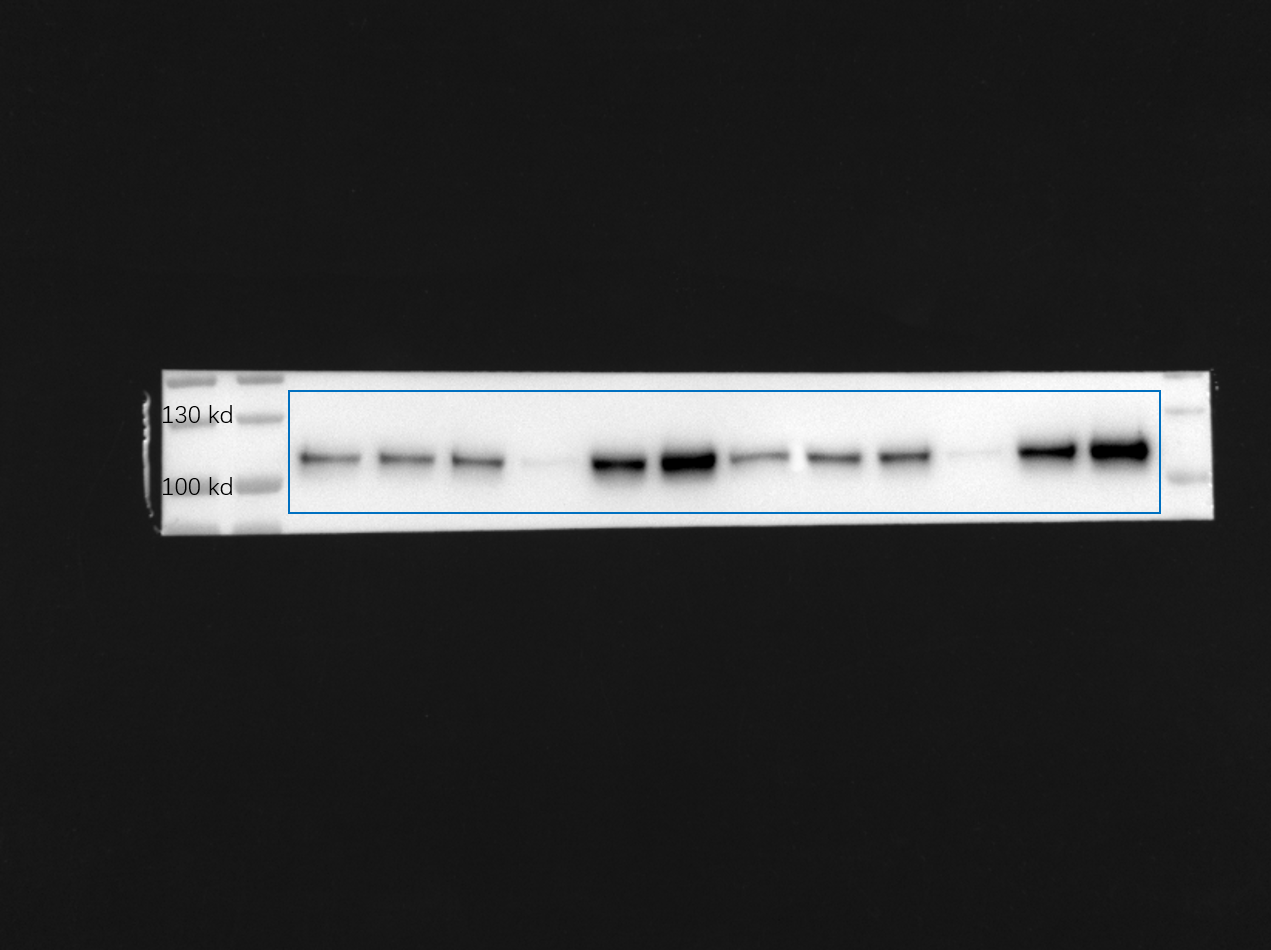

Supplement: Figure 8—source data 2. [file elife-89317-fig8-data2.zip › Figure 8-source data 2/NaK ATPase left labelled.tif]

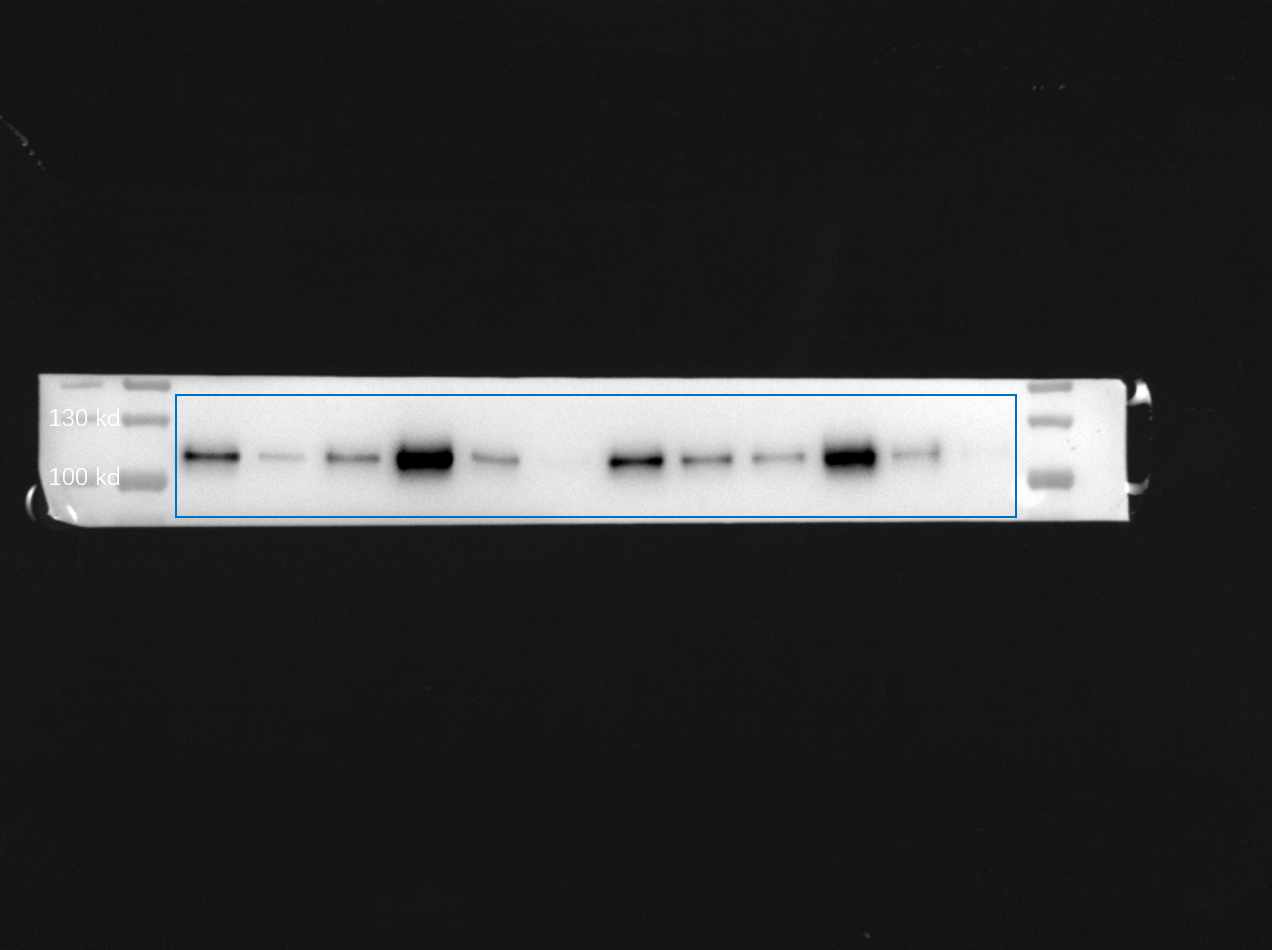

Supplement: Figure 8—source data 2. [file elife-89317-fig8-data2.zip › Figure 8-source data 2/NaK ATPase right labelled.tif]

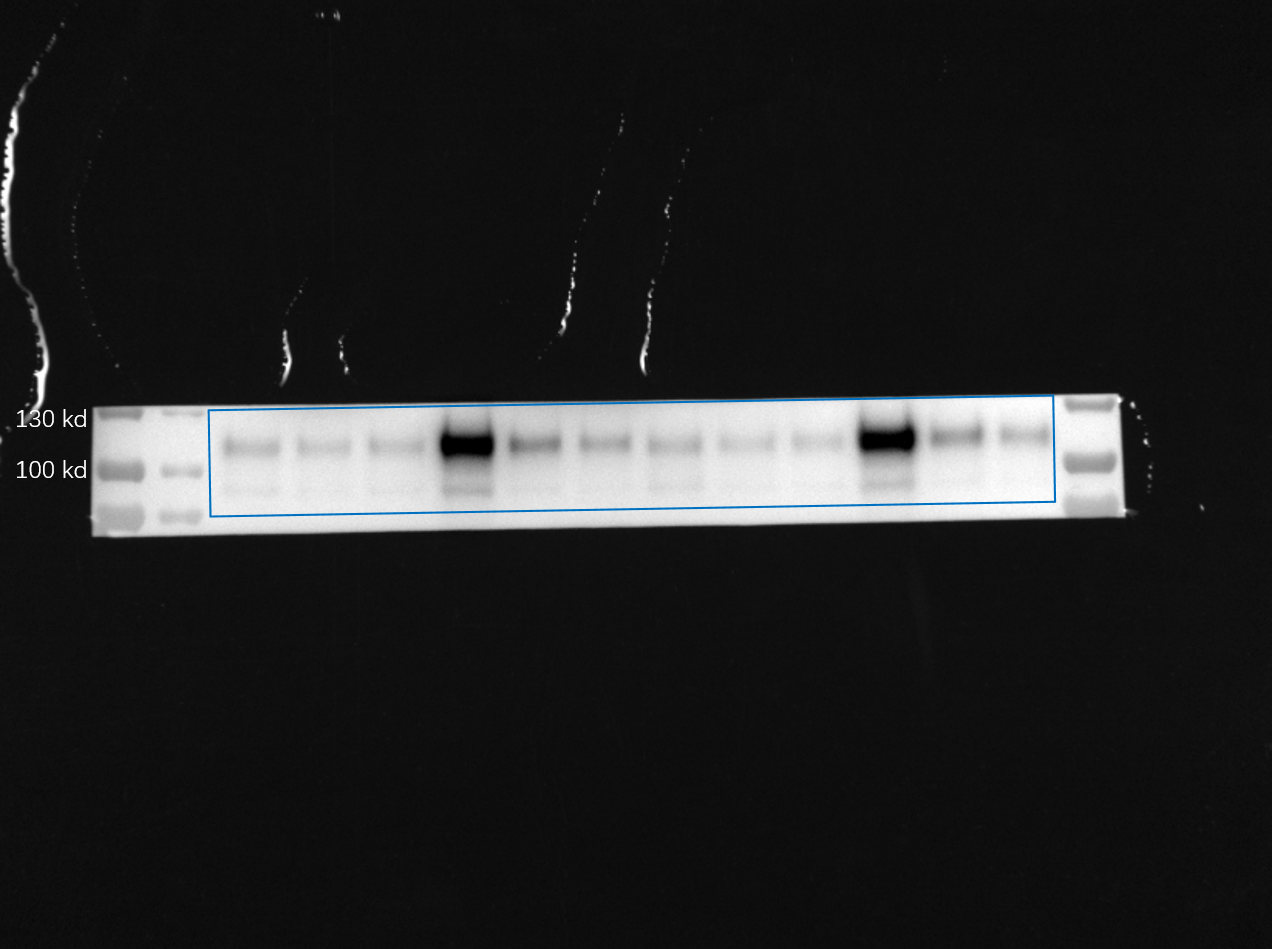

Supplement: Figure 8—source data 2. [file elife-89317-fig8-data2.zip › Figure 8-source data 2/PSD 95 right labelled.tif]

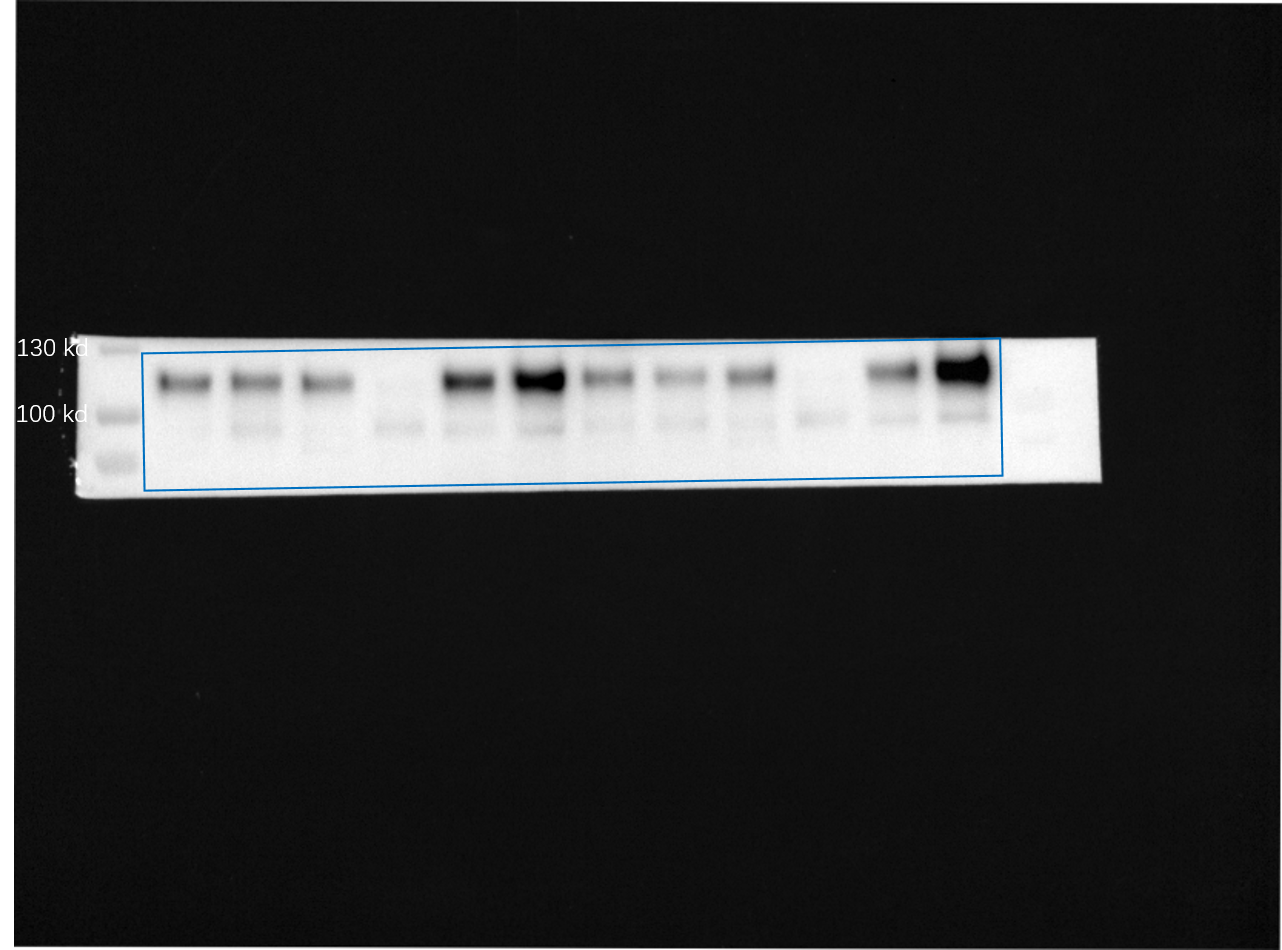

Supplement: Figure 8—source data 2. [file elife-89317-fig8-data2.zip › Figure 8-source data 2/PSD95 left labelled.tif]

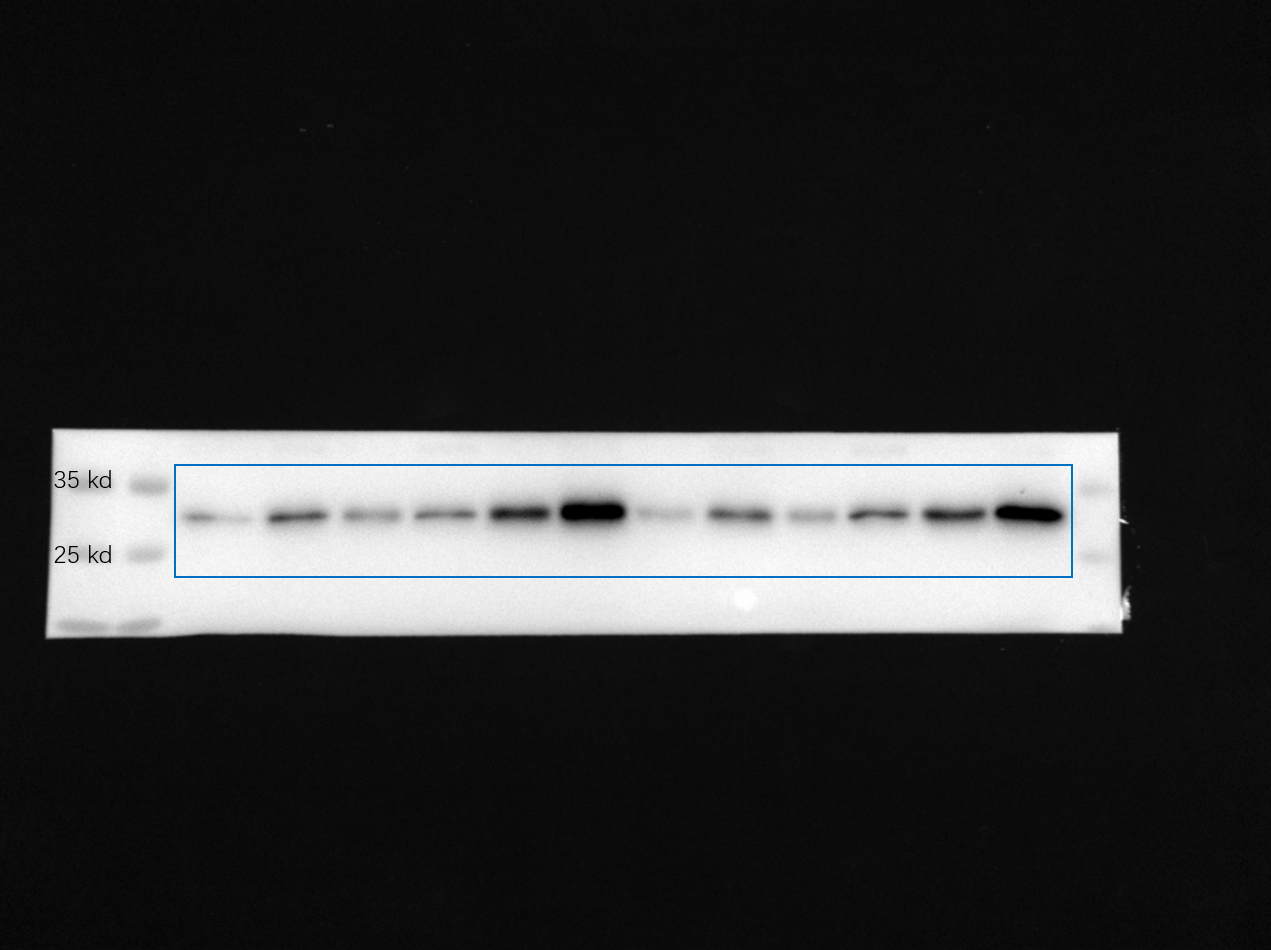

Supplement: Figure 8—source data 2. [file elife-89317-fig8-data2.zip › Figure 8-source data 2/SNAP23 left labelled.tif]
