# Supplementary figures and images for: Suggestion of creatine as a new neurotransmitter by approaches ranging from chemical analysis and biochemistry to electrophysiology (part 2 of 2)
Source: eLife. 2023 Dec 21;12:RP89317. doi: 10.7554/eLife.89317 (PMC10735228; doi:10.7554/eLife.89317)

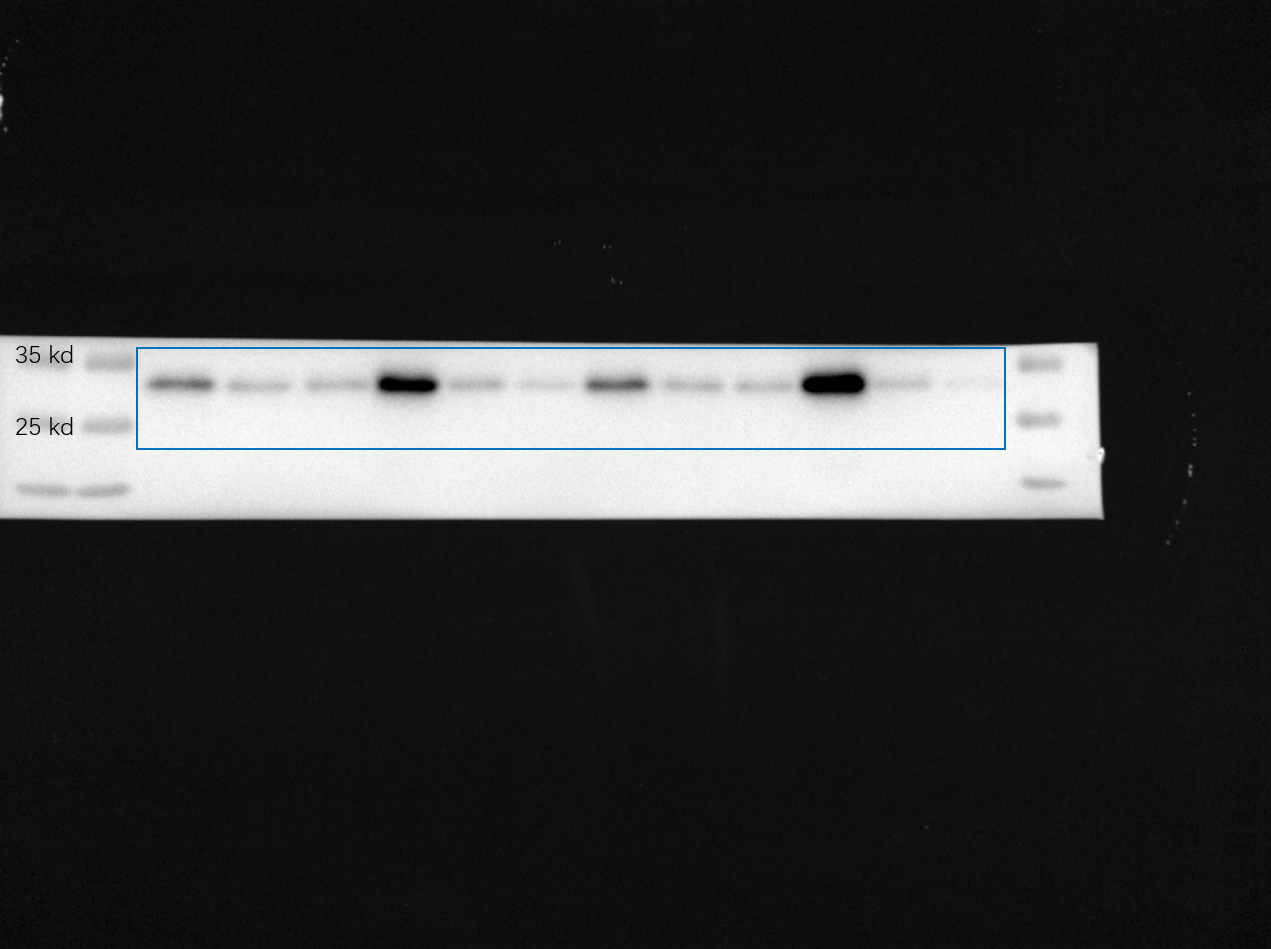

Supplement: Figure 8—source data 2. [file elife-89317-fig8-data2.zip › Figure 8-source data 2/SNAP23 right labelled.tif]

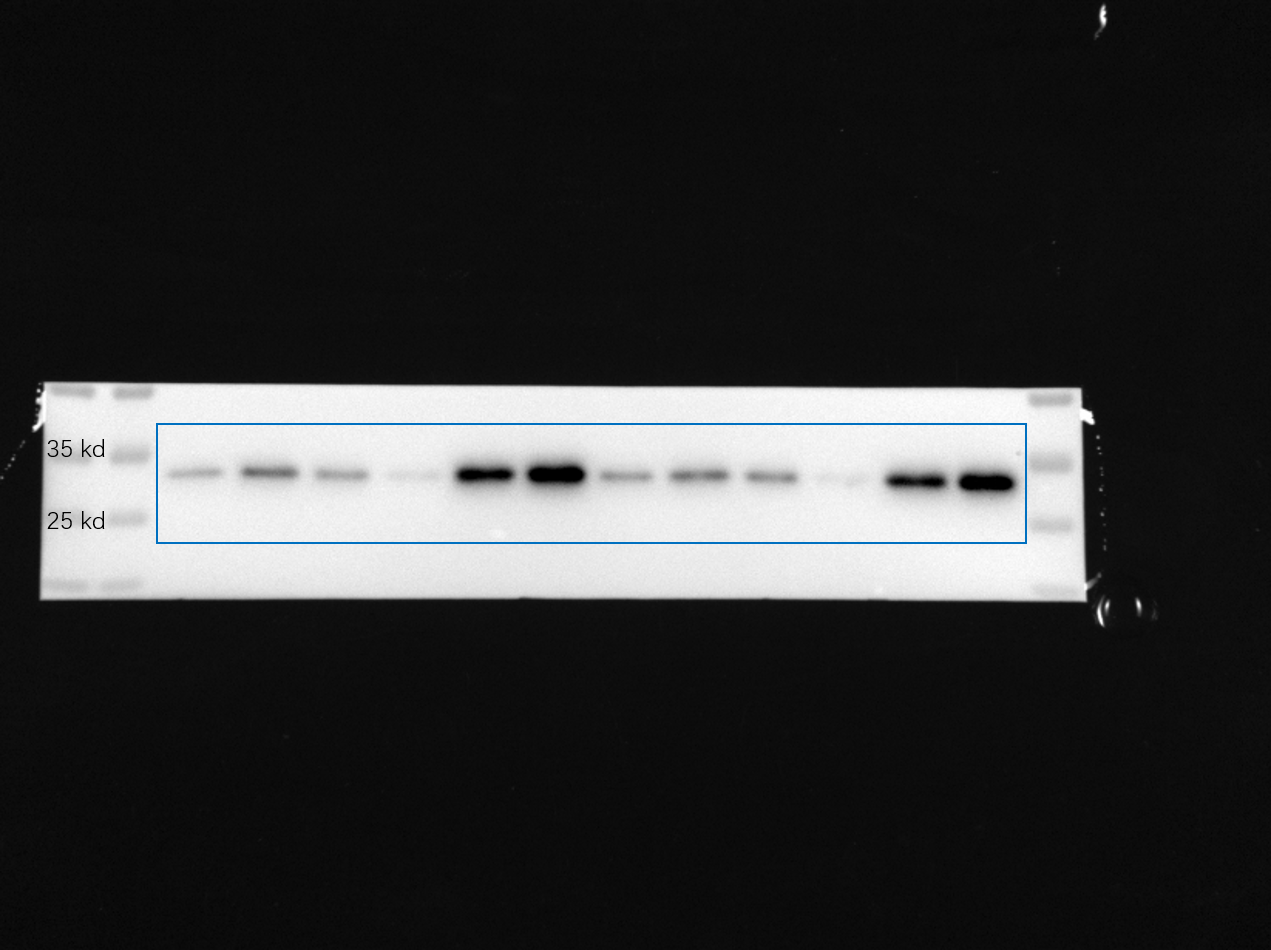

Supplement: Figure 8—source data 2. [file elife-89317-fig8-data2.zip › Figure 8-source data 2/SNAP25 left labelled.tif]

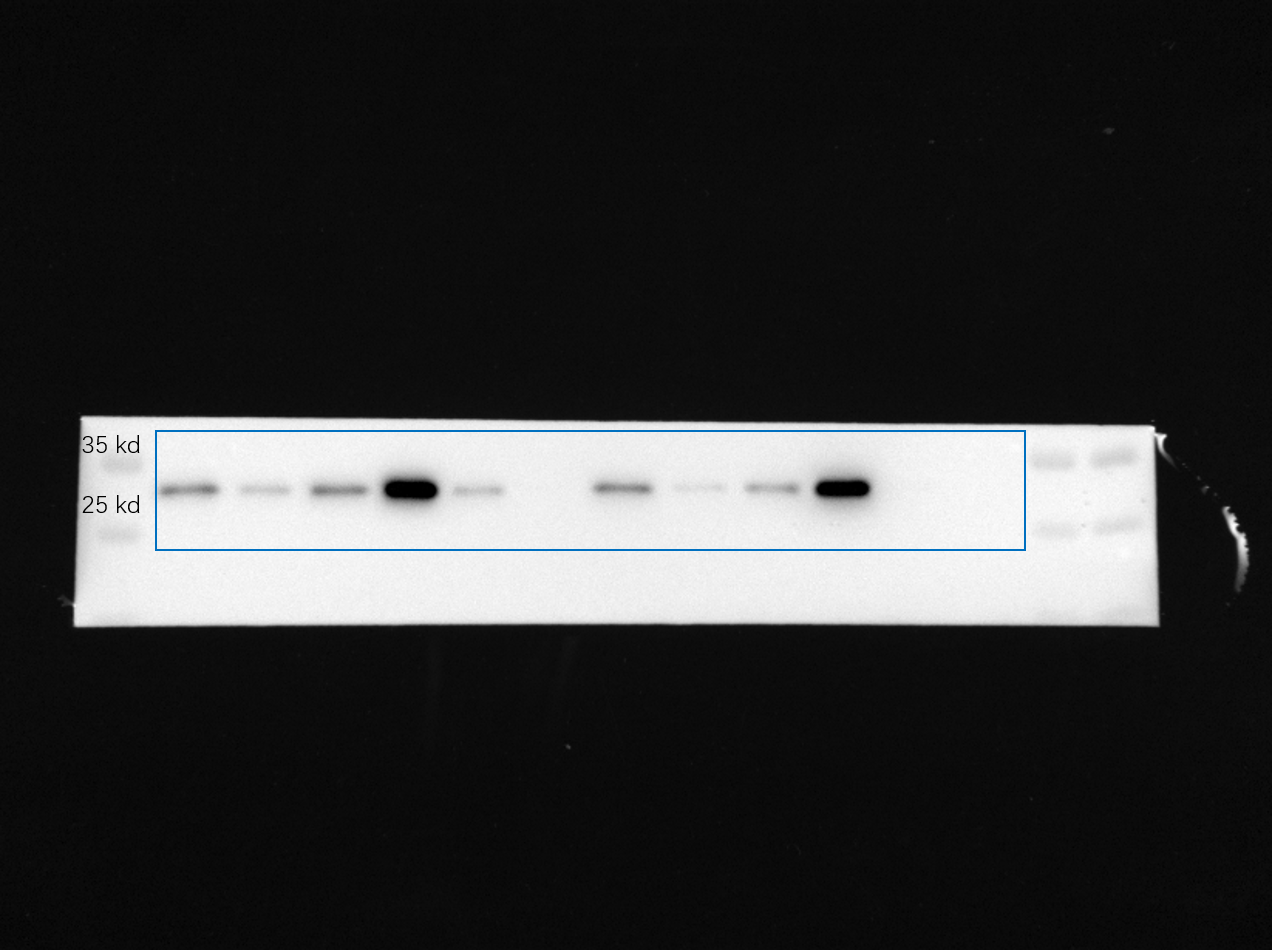

Supplement: Figure 8—source data 2. [file elife-89317-fig8-data2.zip › Figure 8-source data 2/SNAP25 right labelled.tif]

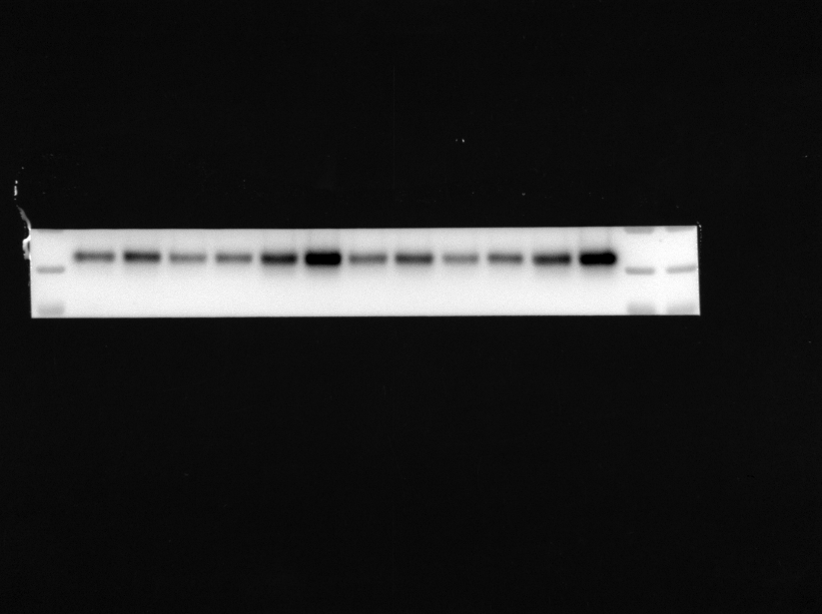

Supplement: Figure 8—source data 2. [file elife-89317-fig8-data2.zip › Figure 8-source data 2/syp left labelled.tif]

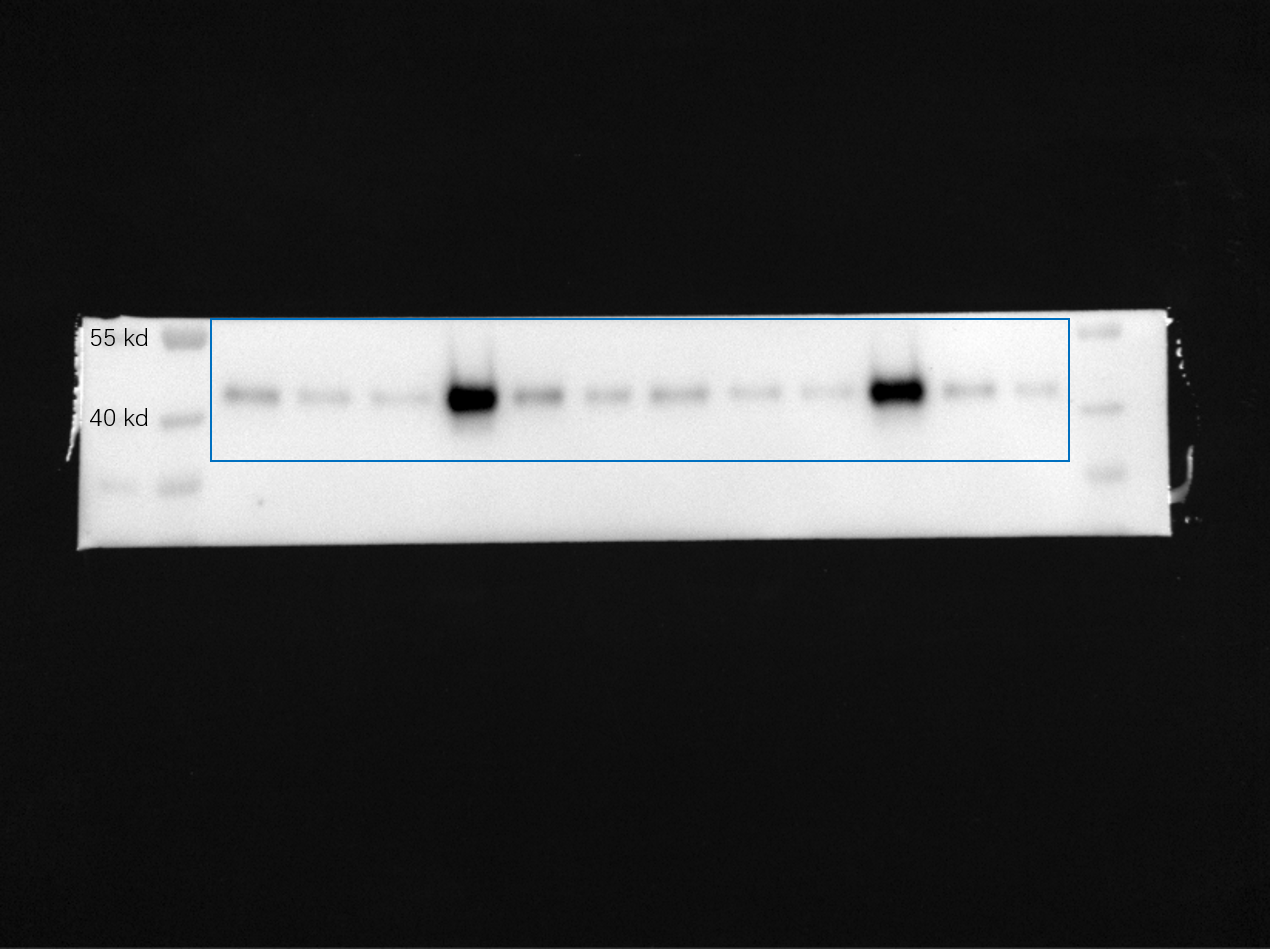

Supplement: Figure 8—source data 2. [file elife-89317-fig8-data2.zip › Figure 8-source data 2/syp right labelled.tif]

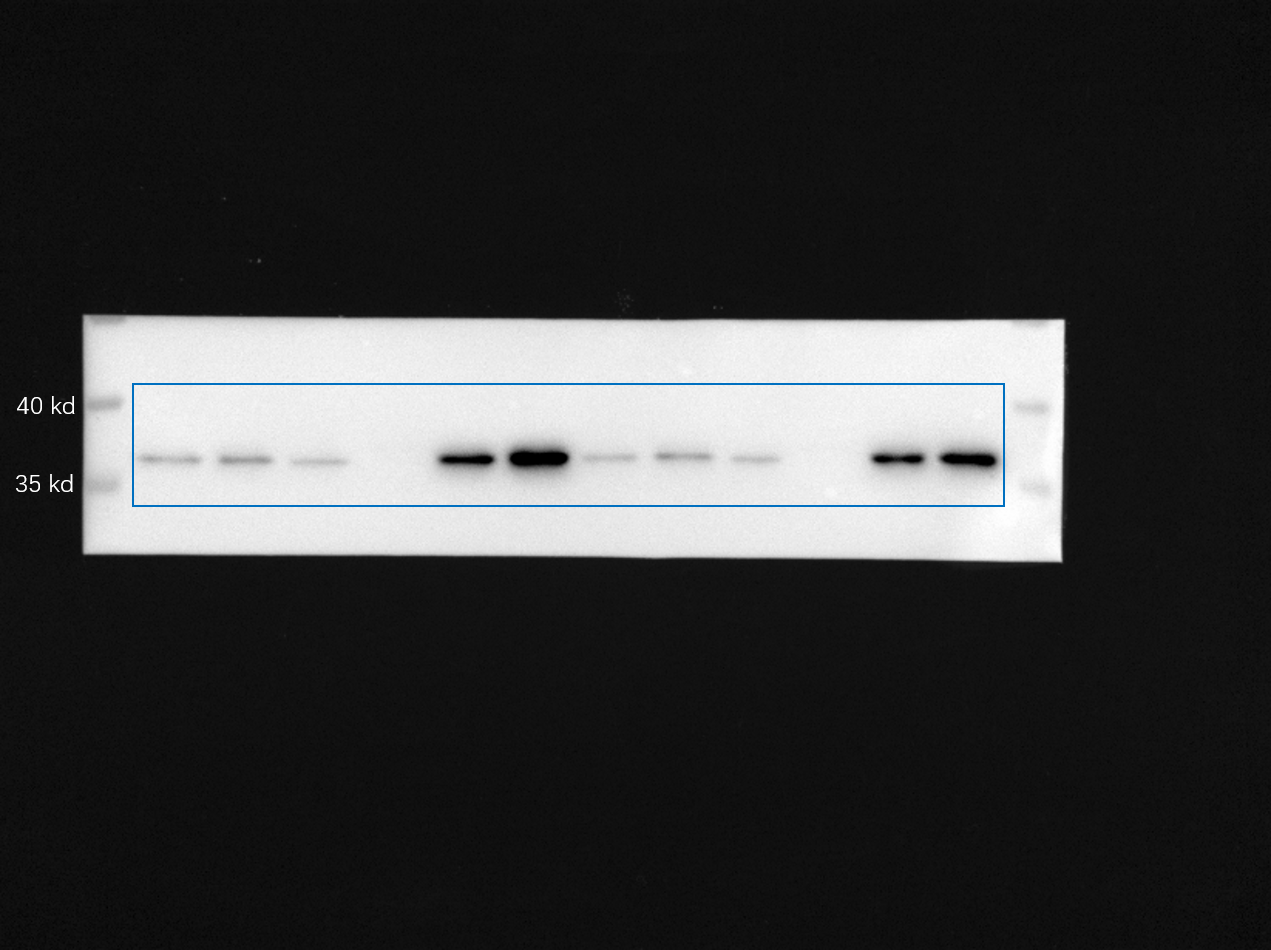

Supplement: Figure 8—source data 2. [file elife-89317-fig8-data2.zip › Figure 8-source data 2/VDAC left labelled.tif]

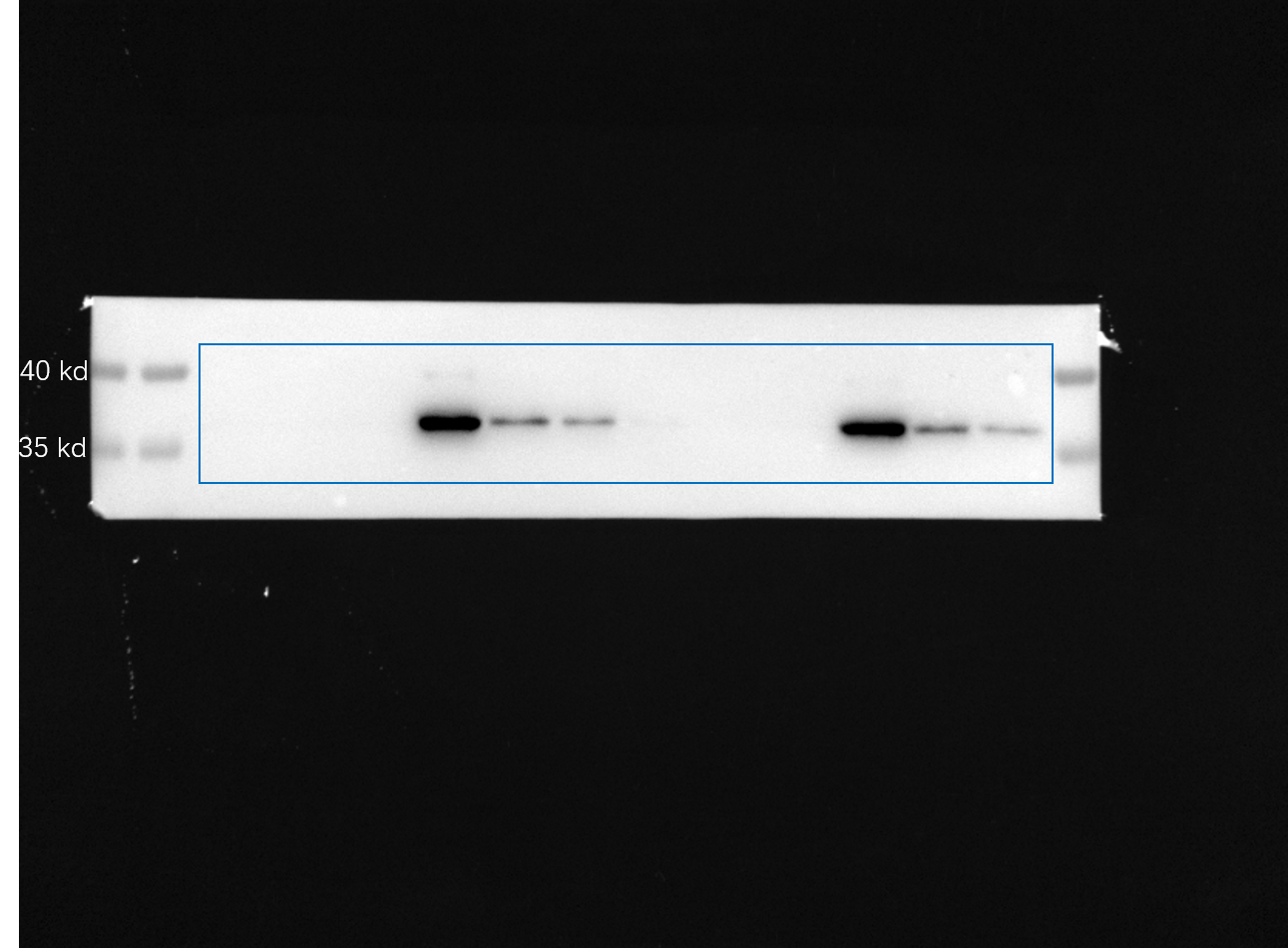

Supplement: Figure 8—source data 2. [file elife-89317-fig8-data2.zip › Figure 8-source data 2/VDAC right labelled.tif]
